# European public perceptions of homelessness: A knowledge, attitudes and practices survey

**DOI:** 10.1371/journal.pone.0221896

**Published:** 2019-09-25

**Authors:** Junie Petit, Sandrine Loubiere, Aurlie Tinland, Maria Vargas-Moniz, Freek Spinnewijn, Rachel Manning, Massimo Santinello, Judith Wolf, Anna Bokszczanin, Roberto Bernad, Hakan Kallmen, Jose Ornelas, Pascal Auquier, HOME-EU consortium study group

**Affiliations:** 1 Aix-Marseille University, School of Medicine—La Timone Medical Campus, CEReSS—Health Service Research and Quality of Life Center, Boulevard Jean Moulin, Marseille, France; 2 Department of Research and Innovation, Support Unit for Clinical Research and Economic Evaluation, Assistance Publique—Hôpitaux de Marseille, Boulevard Jean Moulin, Marseille, France; 3 APPsyCI (Applied Psychology Research Center: Capabilities and Inclusion), ISPA-Instituto Universitário, Rua Jardim do Tabaco, Lisbon, Portugal; 4 FEANTSA, European Federation of National Organisations Working with the Homeless, Chaussée de Louvain, Brussels, Belgium; 5 Department of Psychology, University of Limerick, Limerick, Ireland; 6 Department of Developmental and Social Psychology, University of Padova, Via Venezia, Padova, Italy; 7 Radboud University Medical Center, Radboud Institute for Health Sciences, Impuls—Netherlands Center for Social Care Research, Geert Grooteplein, EZ Nijmegen, The Netherlands; 8 Institute of Psychology, Opole University, Pl. Staszica, Opole, Poland; 9 Rais Fundación, C/ Ardemans, Madrid, Spain; 10 STAD, Stockholm Center for Psychiatry Research and Education, Karolinska Institutet, Norra Stati Onsgatan, Stockholm, Sweden; Jagiellonian University Medical College, POLAND

## Abstract

**Background:**

Addressing Citizen’s perspectives on homelessness is crucial for the design of effective and durable policy responses, and available research in Europe is not yet substantive. We aim to explore citizens’ opinions about homelessness and to explain the differences in attitudes within the general population of eight European countries: France, Ireland, Italy, the Netherlands, Poland, Portugal, Spain, and Sweden.

**Methods:**

A nationally representative telephone survey of European citizens was conducted in 2017. Three domains were investigated: Knowledge, Attitudes, and Practices about homelessness. Based on a multiple correspondence analysis (MCA), a generalized linear model for clustered and weighted samples was used to probe the associations between groups with opposing attitudes.

**Results:**

Response rates ranged from 30.4% to 33.5% (N = 5,295). Most respondents (57%) had poor knowledge about homelessness. Respondents who thought the government spent too much on homelessness, people who are homeless should be responsible for housing, people remain homeless by choice, or homelessness keeps capabilities/empowerment intact (regarding meals, family contact, and access to work) clustered together (negative attitudes, 30%). Respondents who were willing to pay taxes, welcomed a shelter, or acknowledged people who are homeless may lack some capabilities (i.e. agreed on discrimination in hiring) made another cluster (positive attitudes, 58%). Respondents living in semi-urban or urban areas (ORs 1.33 and 1.34) and those engaged in practices to support people who are homeless (ORs > 1.4; p<0.005) were more likely to report positive attitudes, whereas those from France and Poland (p<0.001) were less likely to report positive attitudes.

**Conclusion:**

The majority of European citizens hold positive attitudes towards people who are homeless, however there remain significant differences between and within countries. Although it is clear that there is strong support for increased government action and more effective solutions for Europe’s growing homelessness crisis, there also remain public opinion barriers rooted in enduring negative perceptions.

## Introduction

Available data on homelessness across the currently twenty-eight states of the European Union suggests a steady rise over recent decades, with an increased number of women, youth, families and migrants experiencing homelessness.[1] There is a continuing lack of recent, European-wide quantitative data,[2] but expert estimates from 2009 suggested that, each year, about 4.1 million people in the European Union were unsheltered, or in emergency or temporary accommodation.[3]

Homelessness impacts on both the individual and society. Homeless individuals experience greater physical and mental health risks than the general population, resulting in shorter lifespans, and encounter more barriers to primary healthcare, which leads to higher utilization of more costly healthcare services such as emergency room visits.[4–6] The additional costs of social, healthcare, and housing services for homeless individuals are also high.[4,7–10] Despite this, many European countries have pursued policies aimed at reducing the visibility of homelessness in public spaces,[11,12] whilst cutting spending on services as part of broader austerity programmes.[13] However, changes in the sociopolitical environment, most notably the European migrant crisis, and the ongoing effects of the latest global financial crisis, have given a new urgency for innovative social policies to alleviate homelessness in Europe. In line with this, many European countries are moving towards models which prioritize housing-led approaches and programs combining medical and social support for long-term homeless people with chronic conditions.[14–18]

There is some evidence that public opinion influences policy formation,[19,20] therefore it is important to gain a better understanding of public attitudes towards homelessness in Europe to see whether or not public support could help to shape new homelessness policies. The few studies on public opinion about homelessness were conducted mainly in the United States of America (USA) and provided concurring results.[21–25] In particular, a nationwide survey conducted in 1990 in the USA showed that respondents favored increased government support for a variety of programs addressing homelessness; they also favored a personal tax increase to reduce homelessness in their country.[22] In spite of this, the same survey stressed that people experiencing homelessness continued to be stigmatized as an undesirable marginal group thought to have become homeless due to personal failures and whose presence was believed to have negative effects on neighborhood quality. These results confirmed the findings of earlier surveys [21,24] and were corroborated by a recent assessment of the evolution of public opinion between 1990 and 2017 in the USA.[25] Only one opinion survey was conducted in Europe [26]. This study compared the opinions of respondents from four European countries to those of respondents from the USA. Differences were found, especially with regards to beliefs about whom should be responsible for funding programs addressing homelessness. However, this study had limitations: few countries were investigated, sample sizes were small, and the data collection was spread over 10 years producing different waves, all of which undermined confidence in the findings.

In light of the scarcity of data on the public’s attitudes about homelessness in Europe, key stakeholders would benefit from an updated evaluation of the citizen’s perceptions to better understand public support for programs addressing homelessness[19]. We therefore conducted a study with the objective of: 1) exploring the public’s knowledge, attitudes, and practices (KAP) about homelessness, 2) investigating differences in attitudes within the general population of eight European countries.

## Method

### Survey and participants

Ethics approval for this study has been received from the research ethics committee of Aix-Marseille University (reference number: 2016-01-02-01). A quota telephone survey using landlines and mobile phones was conducted from March 2017 to December 2017. At the beginning of each telephone call, the selected person was able indicate whether he or she wished to participate in the survey. The respondent could also refuse to answer the questions asked and end the interview whenever they wished. All interviews were conducted using Computer Assisted Telephone Interviews (CATI) software. A pilot study was conducted with a sample of 30 French individuals to assess the length of the questionnaire and its intelligibility (face validity). Then, the survey questionnaire was translated into the targeted native languages using the best standardized practice (see Supporting Information S1 File).[27] Participants were randomly selected from opt-in panels to be representative of respective national populations. The sample size for each country was 2,500 people, from which we expected a response rate of approximately 30% to reach our target of 700 surveyed individuals per country. Full details of the survey protocol are available.[28]

Adult citizens (18 years and older) of each of the eight European countries, namely France, Ireland, Italy, the Netherlands, Poland, Portugal, Spain, and Sweden were included. Respondents were informed of the purpose of the study, the intended use of the data, and assured of anonymity.

### Measures

The survey questionnaire was designed to investigate the knowledge, attitudes, and practices (KAP) of the general population regarding homelessness. It was composed of two existing survey questionnaires [24,29] in addition to newly created items designed to answer our research objectives.

Attitudes were defined as the respondents’ beliefs or emotional reactions towards people experiencing homelessness, as well as their intention to act to reduce homelessness. [30] Eleven items addressing a respondent’s perception of the capabilities of people experiencing homelessness, their empowerment,[31] and their integration within the community [32] were created and scored on a four-point scale ranging from strongly agree to strongly disagree. Other items drawn from the Eurobarometer 355 on poverty and social exclusion [33] were added to explore a respondent’s perception of the magnitude (2 items) and main causes of homelessness (12 items), government interventions and spending (8 items) and their inclination to help reduce homelessness (3 items) (S1 Table).

Knowledge based questions addressed three areas: the number of people homeless, and who funded health care and social services for people who are homeless. Respondents’ estimates of the number of people homeless were compared to the latest official estimates available in each country. Answers were classified as ‘good’ if they fell within 20% above or below official figures, ‘partial’ if within 40% and ‘poor’ if outside this range (2017 official estimates were as follows: France: 141,000 [34]; Ireland: 4,875 [35]; Italy: 50,724 [36]; the Netherlands: 30,500 [37]; Poland: 33,408 (Ministry of Family, Labour and Social Policy (MRPPS) cited in [38]); Portugal: 5,265 (Estimate provided by ISPA-Instituto Universitário, and see [39]); Sweden: 30,250 [40]; and Spain: 30,250 [41]). The funding sources of services addressing homelessness were assessed by asking respondents who funded most social or health care services for homeless people, with four response options including ‘government’, ‘non-governmental organization/charities’, ‘Churches and religious communities’, and ‘don’t know’. Answers were classified as ‘good’ if correct, and ‘poor’ if incorrect.

Three items were used to gather information about the self-reported practices regarding homelessness as follows: "Over the past year, have you given money, food, clothing to a homeless person?", "… to a charitable or non-profit organization for homeless people?", and "Over the past year, have you done any volunteer work in a charitable or non-profit organization for homeless people?"; three response options were available (‘yes’, ‘no’, and ‘don’t know’).

Sociodemographic data were then collected.

### Statistical analysis

The distribution of gender, age and education was assessed in each national sample to ensure representativeness. Since discrepancies were found between the distribution of those variables and the 2017 census data obtained through the World Bank [42] and Eurostat [43], weights were applied. Answers to the KAP survey are presented descriptively as the means or proportions, and compared using either analyses of variances for continuous variables or the Chi Squared test (or the Fisher exact test) for qualitative measures.

A multiple correspondence analysis (MCA) was performed on the attitudes items. MCA is an exploratory method used to summarize a complex data set comprised of qualitative variables into simplified dimensions.[44] In MCA, the relations between the variable categories can be visualized through bi-plots. Only those variables contributing strongly to the first two dimensions were kept (based on the maximum percentages of inertia using Burt table analysis). Subsequently, a hierarchical clustering approach was used to create an attitude indicator.

Finally, a generalized linear model for clustered (on country) and weighted samples was used to probe the associations between groups with opposing attitudes and respondents’ knowledge, reported practices, and country of citizenship, adjusted for sociodemographic characteristics. The analysis was conducted with the R Foundation for Statistical Computing, version 3.4.0 [45], using ‘Survey’ and ‘FactoMineR’ packages.

## Results

### Sample description

Response rates to the KAP survey ranged from 30.4% to 33.5%, for a total number of respondents of 5,631 which resulted in 5,295 valid questionnaires. The majority of respondents were women (52% for the overall sample). Across all countries, at least 31% completed higher education, except in Poland and in Italy. Respondents were mainly employed either full-time or part-time, except in France, Italy and most notably in Spain (Table 1). Due to aforementioned discrepancies with census data, results from the weighted samples are presented next.

**Table 1:** Response rates and sociodemographic characteristics of the weighted study population.

		COUNTRIES							
	All	FR	IR	IT	NL	PL	PT	SE	SP
Eligibility* (over 2,500 each)	17,633	2,263	2,261	2,220	2,154	2,206	2,311	2,101	2,117
Complete interviews	5,631	700	701	713	701	708	703	703	702
Response rate	31.9	30.9	31.0	32.1	32.5	32.1	30.4	33.5	33.2
**Characteristics**									
*Gender*									
Woman	51.69	51.60	51.18	51.15	47.26	54.61	54.73	49.03	54.09
*Age groups*									
18–24 years	9.12	6.46	9.13	13.19	6.60	9.24	9.09	8.58	10.48
25.34 years	13.70	10.55	11.86	16.15	10.43	13.85	19.37	14.05	13.02
35.44 years	16.01	18.58	11.86	16.89	13.65	15.01	18.18	18.05	15.57
45.54 years	17.57	25.67	14.90	14.37	18.25	15.58	14.90	17.60	19.61
55.64 years	15.40	12.76	16.35	15.26	19.17	14.86	16.84	16.12	11.98
65.74 years	14.14	10.08	15.71	14.52	15.64	13.42	10.28	13.02	20.21
> = 75 years	14.06	15.91	20.19	9.63	16.26	18.04	11.33	12.57	9.13
*Marital status*									
Married/Civil ^a^	56.54	58.75	66.27	52.99	53.09	62.89	52.96	48.13	57.46
Widowed	8.79	13.16	8.14	10.26	5.61	11.03	6.21	7.17	9.26
Separated/Divorced	10.45	10.91	6.07	11.64	8.92	9.09	15.53	9.87	11.77
Single	24.21	17.17	19.53	25.11	32.37	16.99	25.30	34.83	21.51
*Educational attainment*									
Lower secondary	25.77	22.99	22.20	40.97	25.71	11.54	22.35	18.17	43.55
Upper secondary or vocational	42.90	45.82	39.21	42.99	42.64	62.97	38.60	45.65	24.19
University degree	31.33	31.19	38.58	16.04	31.65	25.49	39.05	36.18	32.26
*Have children*									
Yes ^b^	28.25	28.69	26.83	29.77	29.44	25.09	29.64	30.98	24.72
*Employment status*									
Full-time	37.77	37.34	40.95	32.14	33.24	43.63	53.94	33.59	26.19
Part-time	11.81	8.65	12.17	10.55	16.76	12.59	6.09	16.34	10.79
Unemployed	8.76	6.09	6.82	12.18	5.29	5.40	7.13	3.93	24.29
Retired	30.62	39.10	28.34	34.09	32.35	24.74	26.00	33.89	27.14
Other	11.04	8.81	11.72	11.04	12.35	13.64	6.84	12.25	11.59

*Eligible but not interviewed; included population sample who refused, who were unreachable or with language problem; not eligible population sample included non-targeted population (less than 18 years old or non-European citizens for example) or for whom telephone number were not in service.

FR: France; IR: Ireland; IT: Italy; NL: Netherlands; PL: Poland; PT: Portugal; SE: Sweden; SP: Spain.

a: Marital status: Married or in civil union.

b: The proportion of 'No' answers can de deduced.

The proportion of missing values for all variables in the table was <2%.

### Knowledge about homelessness

Respondents reported a relatively poor knowledge of official estimates of homelessness numbers. For example, around 8% of French respondents came within 20% of French official. Across the overall sample, only 12.9% demonstrated ‘good’ knowledge (Table 2). Notably, Ireland and the Netherlands had a significantly higher percentage of more proximate answers compared to their counterparts (more than 20%). Regarding the sources of funding for homeless services, the proportions of good answers varied between 32% and 69% according to country.

**Table 2:** Knowledge about homelessness and respondent's self-reported practices over the last year (weighted study population).

		COUNTRIES								
Knowledge	All (n(%))	FR (n(%))	IR (n(%))	IT (n(%))	NL (n(%))	PL (n(%))	PT (n(%))	SE (n(%))	SP (n(%))	*p-value*
Magnitude of homelessness ^a^										
Good	608 (12.9)	49 (8.3)	122 (21.1)	36 (6.2)	133 (20.4)	48 (8.0)	65 (10.9)	68 (12.2)	88 (15.7)	**<0.001**
Partial	422 (9.0)	64 (11.0)	77 (13.4)	31 (5.4)	111 (17.0)	24 (4.1)	42 (7.1)	17 (3.0)	56 (10.1)	
Poor	3,668 (78.1)	470 (80.7)	378 (65.5)	517 (88.4)	407 (62.6)	522 (87.9)	488 (82.0)	472 (84.8)	414 (74.2)	
Funding social services for homeless ^b^										
Good	2,405 (45.5)	347 (55.6)	214 (31.8)	240 (36.8)	289 (41.7)	349 (51.9)	250 (37.1)	280 (42.1)	436 (68.8)	**<0.001**
Poor	2,882 (54.5)	277 (44.4)	461 (68.2)	412 (63.2)	403 (58.3)	322 (48.1)	425 (62.9)	385 (57.9)	198 (31.2)	
Funding healthcare services for homeless ^b^										
Good	3,183 (60.3)	385 (61.7)	331 (48.9)	440 (67.5)	449 (64.8)	362 (54.0)	306 (45.3)	452 (69.0)	458 (72.5)	**<0.001**
Poor	2,093 (39.7)	239 (38.3)	345 (51.1)	212 (32.5)	244 (35.2)	308 (46.0)	369 (54.7)	203 (31.0)	173 (27.5)	
**Practices**	All (n(%))	FR (n(%))	IR (n(%))	IT (n(%))	NL (n(%))	PL (n(%))	PT (n(%))	SE (n(%))	SP (n(%))	*p-value*
In person help ^c^										
Yes	3,164 (60.2)	368 (59.0)	415 (61.4)	430 (66.4)	341 (49.7)	379 (56.9)	424 (62.8)	380 (59.0)	429 (67.4)	**<0.001**
Help through organisation ^d^										
Yes	2,969 (56.7)	285 (45.7)	471 (69.8)	309 (48.3)	401 (59.1)	344 (51.9)	498 (73.7)	334 (52.1)	326 (51.3)	**<0.001**
Volunteer work ^e^										
Yes	607 (11.6)	41 (6.6)	109 (16.2)	81 (12.8)	35 (5.1)	58 (8.8)	152 (22.5)	63 (9.8)	67 (10.7)	**<0.001**

FR: France; IR: Ireland; IT: Italy; NL: Netherlands; PL: Poland; PT: Portugal; SE: Sweden; SP: Spain.

a: The following question was used: “Could you tell me approximately the number of homeless people in your Country”. Then, responses about magnitude of homelessness were classified as either “good”, “partial” or “poor” depending on whether the estimates were within 20%, 40% or above 40% of the reference value.

b: The following questions were used: “In (Country), who funds most social services for homeless people?”, “In (Country), who funds most healthcare services for homeless people?”. Then, responses about funding of services were classified as either “good” or “poor” according to the main stream of funding of such services in the target country.

c: To address “In person help”, the following question was used “Over the past year, have you given money, food, clothing to a homeless person?”

d: To address “Help through organization”, the following question was used “Over the past year, have you given money, food, clothing to a charitable or non-profit organisation for homeless people?”

e: To address “Volunteer work”, the following question was used “Over the past year, have you done any volunteer work in a charitable or non-profit organisation for homeless people?”

### Practices about homelessness

More than half of respondents reported having given food, money or offered some assistance to homeless people either in person or through a non-profit organization (Table 2). Fewer reported having volunteered in an organization assisting homeless people; the highest self-reported engagements were found in Portugal and Ireland.

### Attitudes about homelessness

Opinions varied significantly between countries. Most respondents reported seeing few or no homeless people in an average week. In contrast, when asked about the overall trend of homelessness in their country over the last 3 years, respondents reported an increase, most notably in France and in Italy. More than three-quarters of citizens thought the government should bear the main responsibility for the provision of emergency shelters and long-term housing (77.7% and 81.2%, respectively for overall sample) and that government spending (local or central level) on programs addressing homelessness was insufficient, especially in Portugal (85%) and in Spain (88%) (Table 3). Nonetheless, respondents were generally reluctant to pay more taxes to help reduce homelessness (“yes” 31.0%), especially in the Netherlands (18%), Italy (20.5%), and Poland (22.1%).

**Table 3:** Some attitudes of respondents about homelessness (weighted sample) (N(%)).

		COUNTRIES								
	All (n(%))	FR (n(%))	IR (n(%))	IT (n(%))	NL (n(%))	PL (n(%))	PT (n(%))	SE (n(%))	SP (n(%))	*p-value*
***Government spending on Homeless programs***										< .001
Too much	128(2.4)	16(2.6)	4(0.7)	11(1.8)	9(1.3)	24(3.5)	21(3)	39(5.8)	3(0.5)	
Enough	728(13.7)	143(22.9)	74(11)	40(6.2)	134(19.3)	88(13)	54(8)	160(23.9)	35(5.5)	
Too little	4,003(75.6)	435(69.7)	532(78.7)	519(79.6)	493(71)	463(68.9)	574(85)	429(64.1)	559(87.9)	
DK/R	439(8.3)	30(4.8)	65(9.7)	81(12.5)	58(8.4)	98(14.5)	27(4)	41(6.1)	39(6.1)	
***Who should be mainly responsible for providing…***										
*Emergency shelters*										< .001
Government	4,114(77.7)	485(77.8)	563(83.3)	553(84.8)	416(60)	452(67.3)	579(85.6)	572(85.6)	493(77.6)	
NGOs	736(13.9)	123(19.7)	78(11.6)	24(3.7)	199(28.7)	125(18.5)	67(9.9)	22(3.3)	98(15.4)	
Religious groups	178(3.4)	6(1)	7(1.1)	39(6)	39(5.6)	33(4.9)	8(1.1)	20(3)	27(4.2)	
Homeless themselves	144(2.7)	9(1.5)	16(2.4)	11(1.7)	17(2.5)	49(7.3)	7(1)	35(5.3)	1(0.1)	
DK/R	126(2.4)	0(0)	11(1.6)	25(3.8)	23(3.3)	14(2.1)	17(2.5)	19(2.9)	17(2.7)	
*Long-term housing*										< .001
Government	4301(81.2)	461(74)	576(85.2)	567(87)	586(84.5)	424(63.1)	577(85.3)	584(87.4)	525(82.6)	
NGOs	450(8.5)	94(15.1)	26(3.9)	26(4)	53(7.6)	101(15)	66(9.8)	17(2.5)	67(10.5)	
Religious groups	129(2.4)	7(1.1)	8(1.2)	22(3.4)	1(0.2)	23(3.4)	12(1.8)	41(6.2)	14(2.2)	
Homeless themselves	268(5.1)	61(9.8)	44(6.5)	17(2.6)	25(3.6)	106(15.7)	6(0.8)	6(0.9)	3(0.5)	
DK/R	150(2.8)	0(0)	21(3.1)	19(3)	29(4.1)	18(2.7)	15(2.3)	20(3)	27(4.2)	
***To reduce homelessness*, *would you be willing to…***										
*Pay more taxes*										< .001
Yes	1,640(31)	204(32.7)	307(45.4)	134(20.5)	125(18)	148(22.1)	240(35.5)	276(41.4)	205(32.3)	
No	3,325(62.7)	419(67.1)	281(41.6)	411(63)	545(78.5)	500(74.3)	394(58.2)	374(55.9)	401(63.1)	
DK/R	334(6.3)	1(0.2)	88(13)	107(16.4)	25(3.5)	24(3.6)	42(6.2)	18(2.7)	29(4.6)	
*Volunteer*										< .001
Yes	2,390(45.1)	254(40.7)	315(46.6)	289(44.3)	175(25.2)	219(32.6)	510(75.4)	265(39.7)	363(57)	
No	2,665(50.3)	367(58.8)	326(48.3)	242(37.2)	493(71.1)	426(63.4)	156(23.1)	393(58.8)	262(41.1)	
DK/R	243(4.6)	3(0.6)	34(5.1)	121(18.5)	26(3.7)	27(4)	10(1.4)	10(1.6)	12(1.8)	
*Have a homeless shelter near your home*										< .001
Yes	2,656(50.1)	292(46.8)	362(53.6)	288(44.2)	366(52.8)	212(31.5)	353(52.2)	481(71.9)	302(47.5)	
No	2,231(42.1)	326(52.2)	227(33.6)	216(33.2)	298(43)	387(57.6)	304(44.9)	176(26.3)	297(46.7)	
DK/R	411(7.8)	7(1.1)	87(12.8)	148(22.7)	29(4.2)	73(10.8)	19(2.8)	12(1.8)	37(5.8)	

FR: France; IR: Ireland; IT: Italy; NL: Netherlands; PL: Poland; PT: Portugal; SE: Sweden; SP: Spain; DK/R: Don’t know or refusal; NGOs: Non-governmental organizations; ERs: Emergency rooms

When asked to list the three leading causes of homelessness, respondents in every country mentioned job loss (60.3% of overall sample) (S2 Table); addiction was also mentioned in all countries, except in France where indebtedness was mentioned more frequently, closely followed by divorce or the loss of family, rent arrears, with addiction appearing fifth. In all countries, the majority of respondents thought that homeless people had shorter lifespans than members of the general population, were the victims of violence, and were discriminated against when seeking employment. A sizeable proportion of respondents (48.3%) agreed with the statement that homeless people remain homeless by choice; significantly higher rates were observed in Poland (79.9%) and Portugal (67.6%). One third or more stated that homeless people eat at least two meals a day or are able to keep in touch with family and friends. These proportions are doubled in Poland (65.5% and 67.4% respectively). When respondents were asked about the integration of homeless people within the community, more than 40% stated that homeless people had access to paid or unpaid work.

### Exploratory analysis on the attitudes variables

The hierarchical clustering (S1 Fig) performed on the MCA (S2 Fig) clearly identified three clusters of respondents: 1) people without opinion (in green, 12% of respondents); 2) people who thought that being homeless limited a person’s ability, that government interventions were insufficient to address homelessness, and who were willing to act to reduce homelessness (in black, 58% labelled as having “positive attitudes”); 3) people who held opposing opinions on the same items (in red, 30% labelled as having “negative attitudes”).

### Multivariate analysis on attitudes

Nationality was seen to give some indication of attitudes about homelessness, with French and Polish respondents expressing more negative attitudes compared to other surveyed European countries (p<0.001) (Table 4). Gender, age, educational attainment, employment status and marital status were not found to be significantly associated with attitudes. Our knowledge variables were also not significant associated with attitudes.

**Table 4:** Multivariate analysis on attitudes binary variable (positive versus negative attitudes) (N = 4,670, weighted sample).

	Negative attitudes (N = 1,584; 33.92%)	Positive attitudes (N = 1,584; 33.92%)	Univariate analysis	Multivariate analysis		
	N (%) or mean (SD)	N (%) or mean (SD)	*p value*	AOR	95% CI	*p value*
***Sociodemographic variables***						
Age (years)	49.9 (0.6)	53.3 (1.5)	**0.027**	0.99	0.98–0.99	0.121
Gender (Female)	1,549 (53.1%)	811 (47.4%)	0.191	1.09	0.78–1.51	0.601
Education attainment (*ref lower secondary*)						
Upper secondary or vocational	1,159 (40.4%)	776 (46.0%)	0.980	1.35	0.63–2.85	0.433
University Degree	1,030 (35.9%)	452 (26.7%)	0.145	1.64	0.95–2.82	0.073
Marital status (*ref In couple*) Single/widowed/divorced	1,266 (43.4%)	706 (41.2%)	0.608	1.13	0.83–1.52	0.432
Child (Yes)	2,002 (71.7%)	1,171 (74.6%)	0.303	1.20	0.93–1.53	0.142
Working status (Unemployed)^a^	1,510 (48.0%)	795 (53.3%)	0.238	1.18	0.99–1.39	0.055
Living area (*ref Rural*)					
Semi-urban	727 (25.1%)	413 (24.6%)	**0.034**	1.33	1.07–1.66	**0.008**
Urban	1,671 (57.6%)	859 (51.3%)	**0.076**	1.34	1.04–1.73	**0.025**
Country (*ref FR*)						
IR	428 (14.7%)	158 (9.2%)	**<0.001**	1.81	1.58–2.06	**<0.001**
IT	346 (11.8%)	129 (7.5%)	**<0.001**	2.19	1.95–2.47	**<0.001**
NL	371 (12.7%)	237 (13.8%)	**<0.001**	1.27	1.12–1.45	**<0.001**
PL	146 (5.0%)	409 (23.9%)	**<0.001**	0.24	0.21–0.29	**<0.001**
PT	513 (17.6%)	115 (6.7%)	**<0.001**	2.51	2.25–2.80	**<0.001**
SE	405 (13.8%)	231 (13.5%)	**<0.001**	1.40	1.32–1.48	**<0.001**
SP	385 (13.2%)	164 (9.5%)	**<0.001**	2.18	1.736–2.73	**<0.001**
***Practice variables***						
In person help (Yes)	2,021 (69.3%)	776 (46.1%)	**<0.001**	2.51	1.94–3.23	**<0.001**
Help through organisation (Yes)	1,860 (64.0%)	801 (47.7%)	**<0.001**	1.47	1.22–1.77	**<0.001**
Volunteer work (Yes)	445 (15.4%)	107 (6.4%)	**<0.001**	1.67	1.16–2.41	**0.005**
***Knowledge variables***						
Magnitude of homelessness *(ref poor)*						
Partial	130 (8.4%)	248 (9.6%)	0.450	1.05	0.63–1.74	0.842
Good	192 (12.4%)	366 (14.2%)	0.589	1.01	0.61–1.68	0.964
Funding social services for homeless (Good)	902 (52.9%)	1,201 (41.2%)	**0.001**	0.70	0.46–1.06	0.093
Funding healthcare services for homeless (Good)	1,084 (63.6%)	1,676 (57.6%)	0.175	0.86	0.63–1.18	0.359

Generalized linear model (using a quasi-binomial distribution). SD: Standard deviation; AOR: adjusted odds ratio; 95%CI: 95% Confidence Interval; FR: France; IR: Ireland, IT: Italy, NL: The Netherlands; PL: Poland; PT: Portugal; SE: Sweden: SP: Spain.

a: Working status: Employed (including Full-time and Part-time) or Unemployed (including Retired and other working status)

## Discussion

In this study, we surveyed a representative sample of European adult citizens to explore the KAP about homelessness in Europe. Overall, our results revealed that a majority of European citizens reported positive attitudes toward people who are homeless, especially acknowledging that living on the street limits one’s capabilities, and also expressed a willingness for their governments to make a larger budget allocation to address homelessness. Compared to the limited existing European data on the same topic,[26,33] our results also suggest marked differences between countries in attitudes about homeless people.

Although most respondents reported not seeing people who are homeless in an average week, respondents from our study nevertheless recognized an increase in the number of people experiencing homelessness over the last 3 years, as confirmed by recent figures from European countries.[2,37,40,41,46,47] Compared to the 2010 Eurobarometer survey, a much higher proportion reported that many people in their area are homeless (14% vs. 3%).[33] This combination may reflect policies in force across several countries to reduce the visibility of people who are homeless by moving them on from public spaces, banning panhandling, or the hostile design of urban spaces to deter rough sleeping,[11,12] whilst demonstrating that such policies have not been effective at deflecting or ameliorating public concern with or awareness of homelessness.

Interestingly, we found that there were differences of opinion about the causes of homelessness. In Ireland, the Netherlands, Poland, Portugal, and Sweden, addiction was most often identified as the foremost cause of homelessness. Although unemployment was mentioned among the leading causes of homelessness in all countries, only in France, Italy, and Spain was it mentioned more often than other putative causes, reflecting the continuing economic impact, especially in Spain and in Italy, of the 2008 financial crisis. Toro and colleagues had already noted that Italians were more likely to recognize the prominence of economic factors in becoming homeless than other surveyed European countries.[26]

While more than three quarters of respondents acknowledged that people living on the streets experienced discrimination when seeking employment, loss of life expectancy or violence, our results also evidenced a proportion of respondents who thought becoming homeless was a choice, most notably in Italy and in Poland. Similarly, nearly 50% of respondents agreed with the idea that homeless people remain homeless by choice, with significantly higher numbers in Poland (79%) and Portugal (67%). This suggests that ‘homelessness as a choice’ is a widely held opinion in Europe, although this encompasses a complex dynamic in which liberal social values foreground choice in an economic environment in which choices can be severely constrained, particularly for people who experience homelessness. As previous studies have discussed, people who are homeless may themselves consider their position to result from personal choices, whilst acknowledging that these choices were severely restricted.[48] Despite this, another study in the USA showed that around 57% respondents believed laziness to play a role in homelessness,[49] and it is likely that our results suggest that such negative perceptions of people who are homeless persist across Europe, with variances between countries.

Contrary to respondents in Toro and colleagues’ study [26], a majority of respondents were reluctant to pay more taxes to address homelessness, suggesting a possible shift in attitudes since Toro et al’s 1999–2002 timeframe. Considering the responsibility for providing long-term housing, the surveyed countries face similar problems: the production of social housing or capacity thereof remains insufficient despite several action plans, such as Rebuilding Ireland or the Multi-annual plan against poverty and social inclusion 2013–2017 in France. In Sweden, homeless people with low to moderate needs find shelter and ultimately stable housing not within the regular housing market but within the “secondary housing market” administered by local authorities.[50] Tenants within the secondary housing market have difficulty returning to the regular housing market, partly because of the shortage in affordable housing but also because the municipalities have no say in the allocation of social housing in the regular housing market. In France, local authorities can influence the allocation of funds for social housing in favor of homeless people, even more so since the introduction of the Enforceable Right to Housing act in 2007 (DALO in French). However, the continued shortage in social housing stock undermines this right to housing.

The general level of knowledge about homelessness as assessed in our study was low. Regarding the magnitude of homelessness, there are a variety of possible reasons for the generally poor ability to approximate official estimates of homelessness in each country. These could range from a possibly low ability to envisage national figures, to over or under-estimates based on perceived local prevalence or personal perceptions of the extent of homelessness. This would certainly be complicated by the difficulties involved in defining and quantifying homelessness, which can produce widely varying estimates.[51] Depending on the public’s exposure to these debates, such issues could exacerbate disparities between respondent’s estimates and official statistics.

The generally poor understanding of funding for social services for people who are homeless is also notable. In the majority of the surveyed countries (France, Ireland, the Netherlands, Spain, and Sweden) local authorities (municipalities) are enablers of organizations providing social services to homeless people by purchasing these services with local funds or disbursements from the central government. Therefore, although the funding is public, service provision is carried out by secular or faith-based non-governmental organizations (NGOs). This could explain why only a small majority of French, Spanish and a narrow majority of Polish respondents reported accurately that most of the funding of such services is public. This does not negate the fact that some NGOs also benefit from donations, which can represent a sizeable proportion of their budget, if not, for some, their major source of funding. This is the case for the Foundation Abbé Pierre in France, for which donations accounted for 90% of their budget whilst public funds represented only 2% during the 2016–2017 fiscal year.[52] Although Foundation Abbé Pierre is unusual in France, such privately funded organizations are prominent and well-established service providers of emergency services and shelters in Spain and Sweden.[53] Italy represents a special case, as the majority of homelessness services are supplied without public funds.[54] However, the majority of Italian respondents were unaware of this fact, and so appear to have underestimated the involvement of faith-based organizations who are well-established and prominent funders and providers of these services. Conversely, the majority of respondents knew that the government funds healthcare services for homeless people; out of the eight countries, only respondents from Ireland and Portugal thought NGOs played a prominent role in funding healthcare.

Interestingly, demographic variables across Europe as a whole, had no impact on positive attitudes towards people who are homeless. This observation is contrary to other studies that have suggested gender as a predictor of attitudes, with women more likely to consider homelessness as a growing concern, to support increase in federal spending for homelessness and to favor work-oriented interventions as a means of reducing homelessness.[24] Also, Tompsett and colleagues linked higher education with perceiving seeing homelessness as the result of personal flaws,[29] which we did not find in this survey. These studies were conducted in the USA, and this may explain the differences with our study interviewing European citizens, although this certainly warrants further investigation.

### Limitations

There are some limitations to the study design that should be considered while interpreting these findings. Social desirability bias may have led some participants to express more positive attitudes than they hold privately.[55] However, the anonymity of the telephone survey procedure usually allows more self-expression than face-to-face interviews.[56] Interviewers were trained to administer the survey systematically, so to avoid leading responses. To compensate for the potential under sampling of certain groups, interviewers were instructed to call unanswered landlines or cellphones fifteen times before discarding them, and to offer appointments to either start or complete an interview. In addition, following a comparison of respondents’ demographic characteristics to national census data, weights were applied for the analyses of the knowledge, attitudes, and practices of the general population of each country to reflect the national distribution of age, sex, and education.

### Policy implications

This study demonstrates that the majority of European citizens hold positive attitudes about homelessness and wish that European states would do more to reduce it. Our results suggest that policy makers should plan for careful reallocation of funds in favor of programs that effectively address homelessness. However, for these programs to be fully successful, they should aim to address the significant numbers who continue to hold negative attitudes towards people who are homeless. The generally poor knowledge of the magnitude of homelessness, and the funding of social and healthcare services for people who are homeless, suggests that public discussion could be improved to better inform citizens.

## Supporting information

S1 Table Attitudes’ items. (DOC)

S2 Table Attitudes of respondents about homelessness. (DOC)

S1 Fig Factor map. (TIFF)

S2 Fig CA factor map. (TIFF)

S1 File Survey Questionnaire in eight European languages. (DOCX)
