# Supplementary material for: European public perceptions of homelessness: A knowledge, attitudes and practices survey
Source: PLoS One. 2019 Sep 25;14(9):e0221896. doi: 10.1371/journal.pone.0221896 (PMC6760760; doi:10.1371/journal.pone.0221896)
Supplement: S1 Table — (DOC) [file pone.0221896.s001.doc]

**Online supplementary materials**

**S1 Table**: Attitudes’ items

| Items | Variable names | Category of answers |
| --- | --- | --- |
| **Opinion statement about homeless people (capabilities, empowerment and community integration)** | | |
| “Homeless people are the victims of assaults (violence, robbery, threats, and attacks)” | capa.victims | Strongly agree  Somewhat agree  Somewhat disagree  Strongly disagree  Don’t know/Refusal |
| “They are discriminated against in hiring | capa.hiring |
| “They eat at least two meals a day” | capa.meals |
| “They are able to keep in touch with family and friends” | capa.family |
| “They have a shorter life expectancy than the general population” | capa.life |
| “Many remain homeless by choice” | capa.choice |
| “Most have working skills” | emp.skills |
| “They could look after (keep clean, decorate) a home if they had one” | emp.home |
| “They have access to paid or unpaid work (volunteering, internship etc.)” | ci.paid |
| “Their main source of income comes from social welfare benefits” | ci.welfare |
| “They spend much of their time alone, outside of any social network” | emp.alone |
| **Attitudes of respondents about homelessness magnitude** | | |
| “In the last 3 years, would you say the number of homeless people has …” | hlns.country | Strongly increased  Somewhat increased  Somewhat decreased  Strongly decreased  Stayed the same (spontaneous)  Don’t know/Refusal |
| “On average, how many different homeless people do you see per week?” | hp.see | Many people  Some people  A few people  None  Don’t know/Refusal |
| **Attitudes of respondents about the causes of homelessness** | | |
| "In your opinion, what are the THREE reasons that best explain why people become homeless?" | hlns.job  hlns.rent  hlns.catas  hlns.debt  hlns.ill  hlns.addict  hlns.breakup  hlns.mental  hlns.welfare  hlns.immig  hlns.choice  hlns.other | Job lost/ unemployment period  Insufficient income/can’t afford to pay a rent  Home destroyed by a catastrophe (fire, floods, etc.)  Over-indebted  Ill or disabled  Addiction (alcohol, drugs or other types)  Break-up, divorced or loss of family member  Mental health problems  No access to social welfare benefits or support services  Illegal immigration  Own choice  Other reasons ^a^  None  Don’t know/Refusal |
| **Attitudes of respondents about government interventions and spending** | | |
| “Do you think that the Government spends (too much, enough or too little) …… to help homeless people?”  “In general, do you think the Government spends (too much, enough or too little) on social welfare …” | Gvt.hp  Gvt.wlf | Too much  Enough  Too little  Don’t know/Refusal |
| “In your opinion, who should be mainly responsible for providing EMERGENCY SHELTHER for homeless people?” | Resp.shelt | Government  Non-Governmental Organizations/Charities /  Churches and religious communities  Homeless themselves  Don’t know/Refusal |
| “In your opinion, who should be mainly responsible for providing LONG TERM HOUSING for homeless people?” | Resp.house |
| “In your opinion, do services provided in hospitals and emergency rooms meet the needs of homeless people” | Sat.ER | Strongly agree  Somewhat agree  Somewhat disagree  Strongly disagree  Don’t know/Refusal |
| “In your opinion, do services provided by General Practitioners and outpatient specialists meet the needs of homeless people” | Sat.GP |
| “In your opinion, do services provided in emergency shelters meet the needs of homeless people” | Sat.shel |
| “In your opinion, do services provided in transitional shelters meet the needs of homeless people” | Sat.temp |
| **Attitudes of respondents about inclination to help reduce homelessness** |  |  |
| “To reduce homelessness, would you be willing to…” |  | Yes  No  Don’t know/Refusal |
| Pay more taxes? | WTAct.taxes |
| Volunteer? | WTAct.vol |
| Have a homeless shelter near your home? | WTAct.shel |

a: Other reasons related, grouped into one category
