# Supplementary material for: European public perceptions of homelessness: A knowledge, attitudes and practices survey
Source: PLoS One. 2019 Sep 25;14(9):e0221896. doi: 10.1371/journal.pone.0221896 (PMC6760760; doi:10.1371/journal.pone.0221896)
Supplement: S2 Table — (DOC) [file pone.0221896.s002.doc]

**S2 Table: Attitudes of respondents about homelessness (weighted sample) (N(%))**

|  |  | | COUNTRIES | | | | | | | | | | |  |
| --- | --- | --- | --- | --- | --- | --- | --- | --- | --- | --- | --- | --- | --- | --- |
|  | All (n(%)) | | FR (n(%)) | | IR (n(%)) | | IT (n(%)) | NL (n(%)) | | PL (n(%)) | PT (n(%)) | SE (n(%)) | SP (n(%)) | *p-value* |
| ***Homelessness in country over the last 3 years*** | | | | | | | | | | | | | | <.001 |
| Increased | 3,916(73.9) | | 564(90.3) | | 510(75.5) | | 591(90.6) | 408(58.8) | | 425(63.2) | 410(60.7) | 512(76.6) | 496(77.9) |  |
| Same | 591(11.2) | | 41(6.5) | | 63(9.3) | | 29(4.4) | 154(22.2) | | 81(12.1) | 125(18.4) | 67(10.1) | 30(4.8) |  |
| Decreased | 402(7.6) | | 11(1.7) | | 62(9.1) | | 0(0) | 78(11.3) | | 71(10.5) | 75(11.1) | 50(7.5) | 55(8.6) |  |
| DK/R | 390(7.4) | | 9(1.4) | | 41(6.1) | | 32(5) | 53(7.7) | | 96(14.2) | 66(9.7) | 38(5.8) | 55(8.7) |  |
| ***Homeless people seen in a week*** | | | | | | | | | | | | | | <.001 |
| Many people | 721(13.6) | | 85(13.6) | | 23(3.3) | | 119(18.2) | 58(8.3) | | 108(16.1) | 52(7.7) | 101(15.2) | 176(27.6) |  |
| Some people | 1,303(24.6) | | 165(26.4) | | 95(14.1) | | 162(24.8) | 153(22.1) | | 246(36.6) | 180(26.7) | 181(27.1) | 121(19) |  |
| A few people | 1,652(31.2) | | 186(29.9) | | 305(45.1) | | 169(25.9) | 288(41.5) | | 177(26.3) | 183(27) | 176(26.3) | 168(26.5) |  |
| None | 1,546(29.2) | | 187(30) | | 240(35.5) | | 175(26.9) | 190(27.4) | | 138(20.5) | 256(37.9) | 198(29.6) | 162(25.5) |  |
| DK/R | 77(1.4) | | 1(0.1) | | 13(2) | | 28(4.2) | 5(0.7) | | 3(0.5) | 5(0.7) | 13(1.8) | 9(1.4) |  |
| ***Government spending on welfare*** | | | | | | | | | | | | | | <.001 |
| Too much | 430(8.1) | | 177(28.4) | | 50(7.4) | | 15(2.4) | 28(4) | | 49(7.3) | 24(3.5) | 66(9.8) | 21(3.3) |  |
| Enough | 1,177(22.2) | | 191(30.6) | | 227(33.5) | | 62(9.5) | 279(40.2) | | 113(16.8) | 80(11.9) | 163(24.3) | 63(9.9) |  |
| Too little | 3282(61.9) | | 250(40.1) | | 345(51.1) | | 491(75.4) | 338(48.7) | | 415(61.8) | 539(79.8) | 401(59.9) | 502(78.9) |  |
| DK/R | 410(7.7) | | 6(0.9) | | 54(8) | | 83(12.8) | 49(7.1) | | 95(14.1) | 33(4.8) | 40(5.9) | 51(8) |  |
| ***Government spending on Homeless programs*** | | | | | | | | | | | | | | <.001 |
| Too much | 128(2.4) | | 16(2.6) | | 4(0.7) | | 11(1.8) | 9(1.3) | | 24(3.5) | 21(3) | 39(5.8) | 3(0.5) |  |
| Enough | 728(13.7) | | 143(22.9) | | 74(11) | | 40(6.2) | 134(19.3) | | 88(13) | 54(8) | 160(23.9) | 35(5.5) |  |
| Too little | 4,003(75.6) | | 435(69.7) | | 532(78.7) | | 519(79.6) | 493(71) | | 463(68.9) | 574(85) | 429(64.1) | 559(87.9) |  |
| DK/R | 439(8.3) | | 30(4.8) | | 65(9.7) | | 81(12.5) | 58(8.4) | | 98(14.5) | 27(4) | 41(6.1) | 39(6.1) |  |
| ***Who should be mainly responsible for providing…*** | | | | | | | | | | | | | |  |
| *Emergency shelters* |  | |  | |  | |  |  | |  |  |  |  | <.001 |
| Government | 4,114(77.7) | | 485(77.8) | | 563(83.3) | | 553(84.8) | 416(60) | | 452(67.3) | 579(85.6) | 572(85.6) | 493(77.6) |  |
| NGOs | 736(13.9) | | 123(19.7) | | 78(11.6) | | 24(3.7) | 199(28.7) | | 125(18.5) | 67(9.9) | 22(3.3) | 98(15.4) |  |
| Religious groups | 178(3.4) | | 6(1) | | 7(1.1) | | 39(6) | 39(5.6) | | 33(4.9) | 8(1.1) | 20(3) | 27(4.2) |  |
| Homeless themselves | 144(2.7) | | 9(1.5) | | 16(2.4) | | 11(1.7) | 17(2.5) | | 49(7.3) | 7(1) | 35(5.3) | 1(0.1) |  |
| DK/R | 126(2.4) | | 0(0) | | 11(1.6) | | 25(3.8) | 23(3.3) | | 14(2.1) | 17(2.5) | 19(2.9) | 17(2.7) |  |
| *Long-term housing* |  | |  | |  | |  |  | |  |  |  |  | <.001 |
| Government | 4301(81.2) | | 461(74) | | 576(85.2) | | 567(87) | 586(84.5) | | 424(63.1) | 577(85.3) | 584(87.4) | 525(82.6) |  |
| NGOs | 450(8.5) | | 94(15.1) | | 26(3.9) | | 26(4) | 53(7.6) | | 101(15) | 66(9.8) | 17(2.5) | 67(10.5) |  |
| Religious groups | 129(2.4) | | 7(1.1) | | 8(1.2) | | 22(3.4) | 1(0.2) | | 23(3.4) | 12(1.8) | 41(6.2) | 14(2.2) |  |
| Homeless themselves | 268(5.1) | | 61(9.8) | | 44(6.5) | | 17(2.6) | 25(3.6) | | 106(15.7) | 6(0.8) | 6(0.9) | 3(0.5) |  |
| DK/R | 150(2.8) | | 0(0) | | 21(3.1) | | 19(3) | 29(4.1) | | 18(2.7) | 15(2.3) | 20(3) | 27(4.2) |  |
| ***Homeless people's needs are met at…*** | | | | | | | | | | | | | |  |
| *Hospitals and ERs* |  | |  | |  | |  |  | |  |  |  |  | <.001 |
| Strongly Agree/Agree ^a^ | 2,973(56.1) | | 353(56.6) | | 379(56.1) | | 475(72.8) | 278(40) | | 472(70.2) | 318(47.1) | 312(46.6) | 387(60.9) |  |
| Strongly Disagree/Dis. | 1,721(32.5) | | 237(38) | | 222(32.8) | | 110(16.9) | 337(48.6) | | 118(17.6) | 278(41.1) | 263(39.3) | 156(24.5) |  |
| DK/R | 604(11.4) | | 34(5.4) | | 75(11.1) | | 67(10.3) | 79(11.4) | | 82(12.3) | 80(11.8) | 94(14.1) | 93(14.6) |  |
| *Specialist or general practitioner clinics* |  | |  | |  | |  |  | |  |  |  |  | <.001 |
| Strongly Agree/Agree | 2,401(45.3) | | 250(40) | | 283(41.8) | | 368(56.5) | 192(27.7) | | 388(57.8) | 249(36.8) | 335(50.1) | 337(52.9) |  |
| Strongly Disagree/ Dis | 2,207(41.6) | | 336(53.8) | | 281(41.6) | | 194(29.7) | 420(60.6) | | 197(29.3) | 336(49.6) | 243(36.4) | 200(31.4) |  |
| DK/R | 690(13) | | 39(6.2) | | 112(16.6) | | 90(13.8) | 82(11.8) | | 86(12.8) | 92(13.6) | 90(13.5) | 99(15.6) |  |
| *Emergency shelters* |  | |  | |  | |  |  | |  |  |  |  | <.001 |
| Strongly Agree/Agree | 3,170(59.8) | | 332(53.2) | | 464(68.6) | | 356(54.6) | 514(74.1) | | 423(63) | 264(39) | 402(60.1) | 416(65.4) |  |
| Strongly Disagree/ Dis | 1,463(27.6) | | 250(40.1) | | 115(17) | | 189(28.9) | 131(18.9) | | 165(24.5) | 306(45.2) | 174(26.1) | 133(21) |  |
| DK/R | 666(12.6) | | 42(6.7) | | 98(14.4) | | 108(16.5) | 49(7) | | 84(12.5) | 107(15.8) | 92(13.8) | 87(13.6) |  |
| *Temporary shelters* |  | |  | |  | |  |  | |  |  |  |  | <.001 |
| Strongly Agree/Agree | 3,297(62.2) | | 352(56.4) | | 427(63.2) | | 302(46.3) | 557(80.2) | | 430(64) | 414(61.2) | 408(61.1) | 407(64) |  |
| Strongly Disagree/ Dis | 1,250(23.6) | | 229(36.7) | | 120(17.7) | | 220(33.7) | 87(12.5) | | 162(24) | 157(23.2) | 149(22.2) | 127(20) |  |
| DK/R | 751(14.2) | | 43(6.9) | | 129(19.1) | | 130(19.9) | 50(7.2) | | 80(11.9) | 106(15.6) | 112(16.7) | 102(16) |  |
| ***To reduce homelessness, would you be willing to…*** | | | | | | | | | | | | | |  |
| *Pay more taxes* |  | |  | |  | |  |  | |  |  |  |  | <.001 |
| Yes | 1,640(31) | | 204(32.7) | | 307(45.4) | | 134(20.5) | 125(18) | | 148(22.1) | 240(35.5) | 276(41.4) | 205(32.3) |  |
| No | 3,325(62.7) | | 419(67.1) | | 281(41.6) | | 411(63) | 545(78.5) | | 500(74.3) | 394(58.2) | 374(55.9) | 401(63.1) |  |
| DK/R | 334(6.3) | | 1(0.2) | | 88(13) | | 107(16.4) | 25(3.5) | | 24(3.6) | 42(6.2) | 18(2.7) | 29(4.6) |  |
| *Volunteer* |  | |  | |  | |  |  | |  |  |  |  | <.001 |
| Yes | 2,390(45.1) | | 254(40.7) | | 315(46.6) | | 289(44.3) | 175(25.2) | | 219(32.6) | 510(75.4) | 265(39.7) | 363(57) |  |
| No | 2,665(50.3) | | 367(58.8) | | 326(48.3) | | 242(37.2) | 493(71.1) | | 426(63.4) | 156(23.1) | 393(58.8) | 262(41.1) |  |
| DK/R | 243(4.6) | | 3(0.6) | | 34(5.1) | | 121(18.5) | 26(3.7) | | 27(4) | 10(1.4) | 10(1.6) | 12(1.8) |  |
| *Have a homeless shelter near your home* | | | | | | | | | | | | | | <.001 |
| Yes | 2,656(50.1) | | 292(46.8) | | 362(53.6) | | 288(44.2) | 366(52.8) | | 212(31.5) | 353(52.2) | 481(71.9) | 302(47.5) |  |
| No | 2,231(42.1) | | 326(52.2) | | 227(33.6) | | 216(33.2) | 298(43) | | 387(57.6) | 304(44.9) | 176(26.3) | 297(46.7) |  |
| DK/R | 411(7.8) | | 7(1.1) | | 87(12.8) | | 148(22.7) | 29(4.2) | | 73(10.8) | 19(2.8) | 12(1.8) | 37(5.8) |  |
| ***Opinion statements about homeless people (Capabilities, empowerment and community integration)*** | | | | | | | | | | | | | |  |
| *They are the victims of assaults* | | | | | | | | | | | | | | <.001 |
| Strongly Agree/Agree | 3,803(71.8) | | 531(85.1) | | 490(72.5) | | 453(69.5) | 227(32.7) | | 548(81.6) | 531(78.5) | 503(75.2) | 519(81.7) |  |
| Strongly Disagree/ Dis | 1,173(22.1) | | 61(9.7) | | 162(23.9) | | 142(21.8) | 414(59.6) | | 77(11.5) | 122(18.1) | 123(18.4) | 72(11.3) |  |
| DK/R | 323(6.1) | | 32(5.1) | | 24(3.6) | | 56(8.7) | 53(7.7) | | 47(7) | 23(3.4) | 43(6.4) | 45(7) |  |
| *They have a shorter lifespan than the general population* | | | | | | | | | | | | | | <.001 |
| Strongly Agree/Agree | 4,237(80) | | 515(82.6) | | 618(91.5) | | 477(73.2) | 512(73.8) | | 456(67.8) | 589(87.1) | 524(78.4) | 546(85.9) |  |
| Strongly Disagree/ Dis | 715(13.5) | | 89(14.2) | | 35(5.2) | | 98(15) | 141(20.4) | | 92(13.6) | 70(10.3) | 128(19.2) | 62(9.8) |  |
| DK/R | 346(6.5) | | 20(3.2) | | 23(3.3) | | 77(11.8) | 41(5.9) | | 125(18.5) | 17(2.5) | 16(2.4) | 28(4.4) |  |
| *Many remain homeless by choice* | | | | | | | | | | | | | | <.001 |
| Strongly Agree/Agree | 2,559(48.3) | | 306(49.1) | | 254(37.5) | | 242(37.2) | 267(38.5) | | 536(79.8) | 456(67.5) | 214(32) | 284(44.6) |  |
| Strongly Disagree/ Dis | 2,450(46.2) | | 290(46.5) | | 383(56.6) | | 325(49.8) | 384(55.3) | | 92(13.8) | 207(30.7) | 446(66.7) | 323(50.7) |  |
| DK/R | 289(5.5) | | 27(4.4) | | 40(5.8) | | 85(13) | 43(6.3) | | 43(6.4) | 12(1.8) | 9(1.3) | 30(4.7) |  |
| *Most have working skills* | | | | | | | | | | | | | | <.001 |
| Strongly Agree/Agree | 3,868(73) | | 462(74.1) | | 465(68.8) | | 350(53.6) | 556(80.1) | | 508(75.6) | 553(81.8) | 479(71.6) | 496(78) |  |
| Strongly Disagree/ Dis | 1,041(19.6) | | 125(20) | | 150(22.2) | | 165(25.3) | 113(16.2) | | 89(13.2) | 111(16.4) | 178(26.6) | 110(17.4) |  |
| DK/R | 389(7.3) | | 37(5.9) | | 61(9) | | 137(21.1) | 26(3.7) | | 75(11.2) | 12(1.8) | 12(1.8) | 30(4.7) |  |
| *They are discriminated against in hiring* | | | | | | | | | | | | | | <.001 |
| Strongly Agree/Agree | 4,484(84.6) | | 545(87.4) | | 588(87) | | 539(82.7) | 484(69.8) | | 523(77.8) | 638(94.4) | 601(89.8) | 567(89.1) |  |
| Strongly Disagree/ Dis | 544(10.3) | | 61(9.7) | | 56(8.3) | | 55(8.5) | 173(24.9) | | 77(11.4) | 31(4.6) | 57(8.5) | 35(5.5) |  |
| DK/R | 271(5.1) | | 18(2.9) | | 32(4.7) | | 58(8.9) | 37(5.4) | | 73(10.8) | 7(1) | 11(1.7) | 35(5.4) |  |
| *They eat at least two meals a day* | | | | | | | | | | | | | | <.001 |
| Strongly Agree/Agree | 1,744(32.9) | | 177(28.3) | | 182(27) | | 160(24.6) | 164(23.6) | | 440(65.5) | 123(18.2) | 167(24.9) | 331(52.1) |  |
| Strongly Disagree/ Dis | 2,975(56.1) | | 421(67.4) | | 390(57.7) | | 336(51.6) | 459(66.2) | | 127(18.9) | 514(76.1) | 479(71.6) | 249(39.1) |  |
| DK/R | 579(10.9) | | 27(4.3) | | 104(15.3) | | 156(23.9) | 71(10.2) | | 104(15.5) | 39(5.7) | 23(3.5) | 56(8.8) |  |
| *They are able to keep in touch with family and friends* | | | | | | | | | | | | | | <.001 |
| Strongly Agree/Agree | 2,027(38.3) | | 275(44.1) | | 223(33) | | 174(26.7) | 280(40.3) | | 453(67.4) | 158(23.4) | 245(36.6) | 220(34.5) |  |
| Strongly Disagree/ Dis | 2,798(52.8) | | 321(51.5) | | 392(58) | | 351(53.8) | 361(52) | | 142(21.1) | 487(72.1) | 404(60.4) | 340(53.4) |  |
| DK/R | 473(8.9) | | 27(4.4) | | 60(8.9) | | 127(19.5) | 53(7.7) | | 78(11.5) | 31(4.6) | 20(3) | 76(12) |  |
| *They could look after (keep clean, decorate) a home if they had one* | | | | | | | | | | | | | | <.001 |
| Strongly Agree/Agree | 3,830(72.3) | | 468(75.1) | | 516(76.3) | | 339(52) | 512(73.8) | | 453(67.4) | 546(80.8) | 527(78.8) | 468(73.7) |  |
| Strongly Disagree/ Dis | 992(18.7) | | 125(20.1) | | 109(16.1) | | 148(22.8) | 141(20.3) | | 95(14.2) | 111(16.5) | 134(20.1) | 127(19.9) |  |
| DK/R | 476(9) | | 30(4.8) | | 51(7.6) | | 164(25.2) | 41(5.9) | | 124(18.4) | 18(2.7) | 7(1.1) | 41(6.4) |  |
| *They have access to paid or unpaid work* | | | | | | | | | | | | | | <.001 |
| Strongly Agree/Agree | 2,136(40.3) | | 208(33.4) | | 233(34.4) | | 175(26.9) | 312(45) | | 459(68.3) | 214(31.6) | 272(40.6) | 264(41.5) |  |
| Strongly Disagree/ Dis | 2,452(46.3) | | 351(56.3) | | 346(51.2) | | 300(46) | 327(47.1) | | 95(14.1) | 387(57.2) | 342(51.1) | 305(48) |  |
| DK/R | 710(13.4) | | 65(10.4) | | 97(14.4) | | 177(27.1) | 55(7.9) | | 119(17.6) | 76(11.2) | 56(8.3) | 67(10.5) |  |
| *Their main source of income comes from social welfare benefits* | | | | | | | | | | | | | | <.001 |
| Strongly Agree/Agree | 3,686(69.6) | | 426(68.3) | | 507(75) | | 255(39.1) | 529(76.3) | | 492(73.2) | 500(73.9) | 534(79.8) | 443(69.7) |  |
| Strongly Disagree/ Dis | 1,070(20.2) | | 159(25.5) | | 121(17.9) | | 242(37.2) | 123(17.8) | | 92(13.8) | 130(19.2) | 100(15) | 102(16) |  |
| DK/R | 542(10.2) | | 38(6.2) | | 48(7.1) | | 155(23.8) | 41(6) | | 87(13) | 47(6.9) | 35(5.2) | 91(14.3) |  |
| *They spend much of their time alone* | | | | | | | | | | | | | | <.001 |
| Strongly Agree/Agree | 4116(77.7) | | 442(70.8) | | 567(84) | | 459(70.3) | 549(79.1) | | 535(79.6) | 615(91) | 508(76) | 441(69.3) |  |
| Strongly Disagree/ Dis | 829(15.6) | | 150(24.1) | | 79(11.6) | | 128(19.6) | 111(16) | | 81(12) | 45(6.7) | 134(20) | 101(16) |  |
| DK/R | 353(6.7) | | 32(5.1) | | 30(4.4) | | 66(10.1) | 34(4.9) | | 56(8.4) | 15(2.3) | 27(4) | 94(14.7) |  |
| ***Three main causes of homelessness ^b^*** | | | | | | | | | | | | | |  |
| *Job loss* | 3,196(60.3) | 465(74.6) | | 388(57.4) | | 369(56.6) | | 303(43.7) | 401(59.7) | | 482(71.4) | 255(38.1) | 532(83.7) | <.001 |
| *Rent arrears* | 1,621(30.6) | 209(33.5) | | 301(44.5) | | 157(24.1) | | 263(38) | 141(21) | | 190(28.1) | 173(25.8) | 187(29.4) | <.001 |
| *Catastrophe ^c^* | 248(4.7) | 15(2.4) | | 27(4) | | 35(5.3) | | 30(4.3) | 75(11.2) | | 30(4.4) | 16(2.4) | 20(3.2) | <.001 |
| *Over indebtedness* | 1,259(23.8) | 249(39.8) | | 91(13.5) | | 98(15.1) | | 278(40) | 96(14.3) | | 180(26.7) | 110(16.5) | 156(24.6) | <.001 |
| *illness or disability* | 565(10.7) | 144(23) | | 61(9.1) | | 65(10) | | 18(2.6) | 81(12) | | 38(5.7) | 83(12.4) | 74(11.7) | <.001 |
| *Addictions ^d^* | 3,148(59.4) | 198(31.7) | | 455(67.4) | | 276(42.3) | | 398(57.4) | 519(77.3) | | 499(73.8) | 431(64.5) | 371(58.3) | <.001 |
| *Divorce/ loss family ^e^* | 1,632(30.8) | 214(34.4) | | 215(31.8) | | 187(28.7) | | 246(35.4) | 198(29.4) | | 248(36.7) | 197(29.4) | 127(20) | <.001 |
| *Mental health problem* | 1,361(25.7) | 127(20.4) | | 233(34.5) | | 67(10.3) | | 178(25.7) | 109(16.3) | | 188(27.7) | 338(50.6) | 120(18.9) | <.001 |
| *Lack of welfare ^f^* | 563(10.6) | 31(5) | | 78(11.5) | | 55(8.5) | | 163(23.4) | 98(14.5) | | 30(4.5) | 60(8.9) | 49(7.6) | <.001 |
| *Illegal immigration* | 748(14.1) | 129(20.7) | | 45(6.7) | | 182(27.9) | | 84(12.2) | 20(3) | | 74(10.9) | 93(14) | 120(18.8) | <.001 |
| *Own choice ^g^* | 397(7.5) | 49(7.9) | | 17(2.6) | | 73(11.2) | | 20(2.8) | 165(24.5) | | 17(2.5) | 21(3.2) | 35(5.5) | <.001 |
| *Other ^h^* | 159(3) | 12(1.9) | | 24(3.6) | | 10(1.5) | | 10(1.5) | 43(6.5) | | 12(1.8) | 34(5.1) | 12(1.9) | <.001 |

FR: France; IR: Ireland; IT: Italy; NL: Netherlands; PL: Poland; PT: Portugal; SE: Sweden; SP: Spain; DK/R: Don’t know or refusal; NGOs: Non-governmental organizations; ERs: Emergency rooms; Dis: Disagree

a: Strongly agree/Agree answers were grouped together; Strongly disagree/Disagree answers were grouped together.

b: Respondents were asked " In your opinion, what are the THREE reasons that best explain why people become homeless?" Respondents gave spontaneous answers, no list was provided.

c: Home destroyed by catastrophe (fire, flood, earthquake etc.)

d: Addictions to drug, alcohol or others substances

e: Divorce, break-up, loss of a family member

f: Lack of access to social welfare benefits or support services

g: People choose to become homeless

h: Other reasons related were grouped into one category
