# Supplementary figures and images for: European public perceptions of homelessness: A knowledge, attitudes and practices survey
Source: PLoS One. 2019 Sep 25;14(9):e0221896. doi: 10.1371/journal.pone.0221896 (PMC6760760; doi:10.1371/journal.pone.0221896)

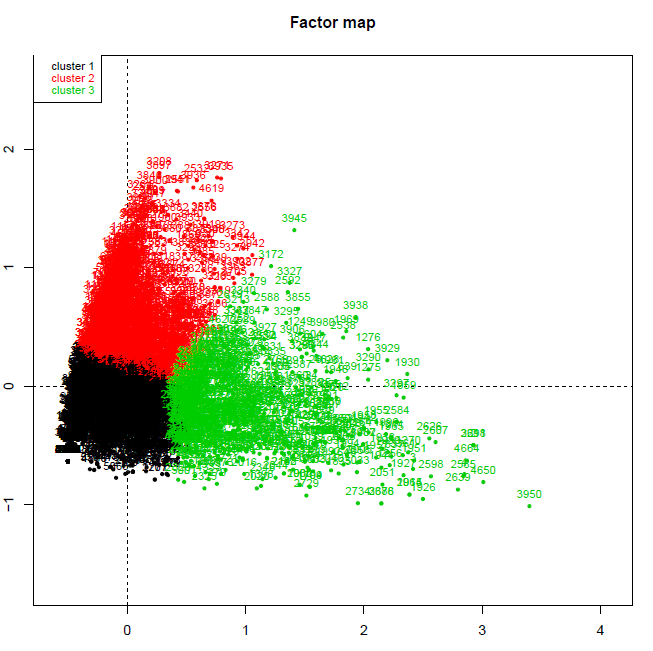

Supplement: S1 Fig — (TIFF) [file pone.0221896.s003.tiff]

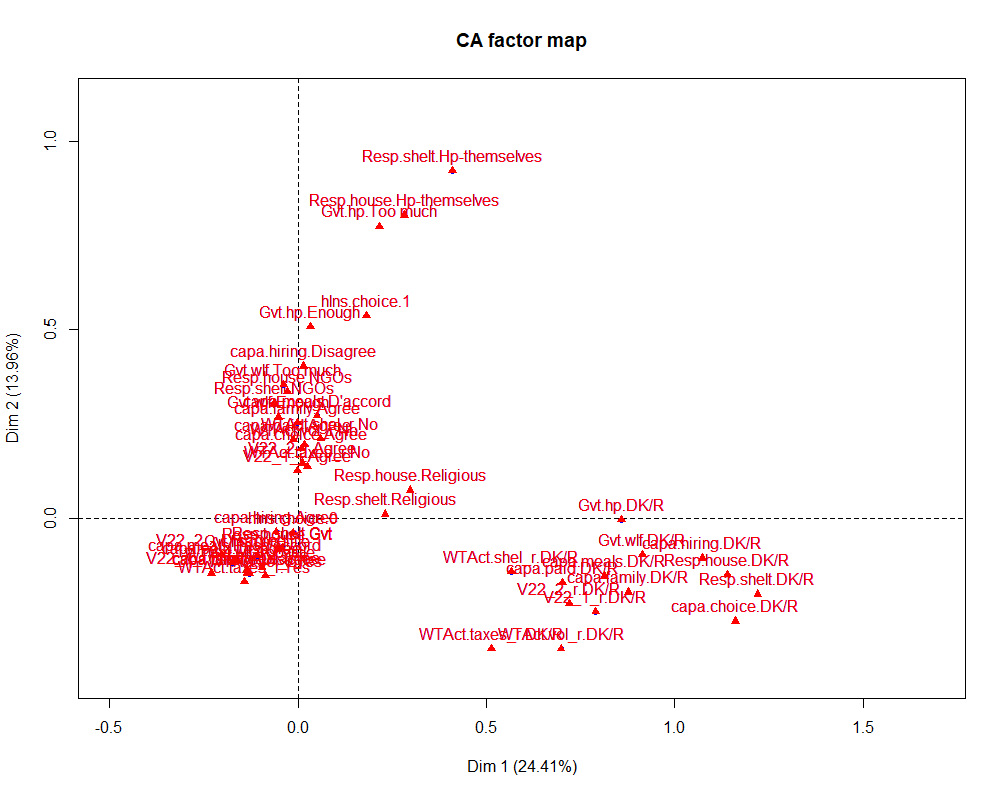

Supplement: S2 Fig — (TIFF) [file pone.0221896.s004.tiff]
