# Supplementary material for: European public perceptions of homelessness: A knowledge, attitudes and practices survey
Source: PLoS One. 2019 Sep 25;14(9):e0221896. doi: 10.1371/journal.pone.0221896 (PMC6760760; doi:10.1371/journal.pone.0221896)
Supplement: S1 File — (DOCX) [file pone.0221896.s005.docx]

| CITIZEN SURVEY OF  THE PUBLIC'S OPINION  ON HOMELESSNESS AND PREFERENCES |
| --- |
| IDENTIFICATION NUMBER: /__/__/__/__/__/  DATE: (____/____/____)  INTERVIEW START TIME : (____:____) FINISH TIME: (____:____)  INTERVIEWER : _________________________ INTERVIEWER CODE : __________ |

1. ***INTRODUCTION***

*[INTERVIEWER’S SCRIPT]*

**Hello, my name is ----------------------------------. I'm calling from** *(Fill in according to Country)* **University. Your household has been randomly chosen to take part in a European survey on (***NATIONALITY***) OPINION ABOUT HOMELESSNESS.**

**Are you 18 or older?**

*[If yes, then go to part II aVAILABILITY FOR THE SURVEY]*

*[If no, then ask]:* **Could I speak to anyone who’s 18 or older in your household right now?**

1. *[If yes, when the person comes to the phone, continue with the following]:* **Hello, my name is ----------------------------------. I'm calling from** *(Fill in according to Country)* **University. Your household has been randomly chosen to take part in a European survey on THE PUBLIC’S OPINION ABOUT HOMELESSNESS.** *[and go to part II aVAILABILITY FOR THE SURVEY]*
2. *[If no, adult person is not at home then ask*:] **When would be a good time to reach that person at home?**

[LOG FOR CALL-BACK]: Date: _______ Time: ________NAME (First only) ________ **Thank you. We’ll call back then.**

1. [If don’t know or no:]: **Thanks. We’ll try again some other time**;
2. ***aVAILABILITY FOR THE SURVEY***

**Can we talk for about 20 minutes?**

1. [*If yes]:* **Thanks!** *(and go to section V* *SETTING UP THE INTERVIEW)*
2. *[If no, ask*:] **When would be a convenient time for me to call you back?**

[*LOG FOR CALL-BACK*:] Date:__________ Time: _______________ **Thank you. We’ll call back later.**

- - *[If no or doesn’t know, say*:] **We would really like to get your views on this subject. Are you sure we can't talk now or at any other time?**
    - *[If answer is yes].* **Thanks!** *(and go to section V* *SETTING UP THE INTERVIEW]*
    - [*If answer Yes but another time, note Date/time:]*

Date:_______________ Time: _______________ **Thank you. We’ll call back later.**

- - - *[If no, go to section III Refusal].*

1. ***REFUSAL***
   1. **We understand you do not want to participate; but could you help us analyze refusals by telling us your age, your highest level of education and your occupational status?**

1 = Yes

2 = No

- 1. **Year of birth: ____________________**
  2. **Highest educational level :**

1 = Postgraduate degree (Masters, PhD, etc/ 4 years or more of post-secondary education)

2 = Bachelor or equivalent (up to 3 years of post-secondary education)

3 = Higher education (up to 2 years of post-secondary education)

4 = Leaving Certificate

5 = High school with no Leaving Cert

6 = Junior Certificate

7 = Primary school

8 = Have not attended school

9 = Other qualification

10= DK

11= NA

- 1. **Occupational status :**

1 = Working Full time (35 hours or more the week)

2 = Working part time (8 to 35 hours per week)

3 = Working part time occasionally (less than 8 hours)

4 = Full time Student

5 = Retired

6 = Unemployed

7 = Not working for other reasons

8 = DK

9 = NA

- 1. **How many people live in your household, including yourself? ____**
  2. **How many children under 18 live in your household? ______**

**Thanks for your help and sorry for bothering you.**

1. ***THE Respondent DOES NOT SPEAK*** *(to adapt according to the language****) ADEQUATELY***

*[Note: If you reach someone who speaks a Foreign language (and whose French (adapt to COUNTRY) is obviously insufficient to complete interview), say:****]***

***I’m sorry; I only speak*** *(to adapt according to the language).* ***Thank you for your time, Good bye.***

1. ***SETTING UP THE INTERVIEW***

*[INTERVIEWER’S SCRIPT]*

**This telephone survey is part of a major survey conducted in 8 European countries. The goal is to measure citizens' opinions on the issue of homelessness and understand their expectations on this issue. Please note that during this interview only your opinion concerning the situation in** *(COUNTRY)* **will be addressed. Your answers will remain strictly anonymous. I will not ask you for your name or your first name. If you do not wish to answer some questions, you may simply say so.**

**Do you agree to participate in this survey measuring citizens’ opinions on homelessness?** YES

NO *(go to section III REFUSAL)*

*[If yes]* **All right then, so now let's begin!**

*[INTERVIEWER’S SCRIPT]* **I will start by asking you general questions about homeless people. First of all, you should know that we define homelessness as sleeping in the street, in a car, or living in an emergency or temporary shelter.**

| *[Note :* ***Never*** *read out DK or R modalities]* | DK= Doesn’t know  R= refuses to answer |
| --- | --- |
|  |  |
| 1. **So, have you ever been homeless?**   *[note : even for one night, count as YES]* | 1 = YES  2 = NO *(skip to Q4)*  3 = R(*Skip to Q4*) |
| 1. *[If answers “yes” to Q1]:* **When was**   **It?** *[read out items]* | 1 = In the past 12 months  2 = 1-2 years ago  3 = 3-4 years ago  4 = 4-5 years ago  5 = More than 5 years ago  6 = DK  7 = R |
| 1. *[If answers “yes” to Q1]:* **How much time in total have you been homeless over your life?** *[read out items]* | 1 = Less than a week  2 = Less than a month  3 = Less than a year  4 = Less than two years  5 = Less than four years  6 = More than four years  7 = DK  8= R |
| 1. **Has any member of your family, friend or acquaintance ever been homeless?** | 1 = YES  2 = NO *(Skip to Q6)*  3 = DK (*Skip to Q6)*  4 = R (*Skip to Q6)* |
| 1. *[If family or friend ever homeless = yes]* **Was it …** | 1 = Parent *[father, mother]*  2 = Child  3 = Brother/sister  4 = Husband/wife/ partner  5 = Friend  6 = Other relative  7 = Acquaintance  8 = DK  9 = R |
| 1. **Could you tell me approximately how many homeless people there are, in** *(country)***?**   *[the respondent should give a number- not a percentage]* | /_______________/ |
| 1. **In your opinion, what is the percentage of homeless people with...**   *[the respondent should give a percentage]* |  |
| 1. Mental disorders? | _______% |
| 1. Addiction problems (alcohol, drugs)? | _______% |
| 1. **In *(country),* who funds most social services for homeless people***?*   *[Only one answers is expected]* | 1 = Government  2 = Non-Governmental Organizations/Charities  3 = Churches and religious communities  4 = DK  5 = R |
| 1. **In** *(country),* **who funds most healthcare facilities for homeless people?**   *[Only one answers is expected]* | 1 = Government  2 = Non-Governmental Organizations/Charities  3 = Churches and religious communities  4 = DK  5 = R |
| 1. **In the area where you live, would you say there are many,** **some, a few or no homeless people?**   *[In the area where you live = In your neighborhood]* | 1 = Many people  2 = Some people  3 = A few people  4 = None (*Skip to Q12)*  5 = DK  6 = R |
| 1. **On average, how many different homeless people do you see per week?**   *[If respondent sees the same person several times a week, it counts as 1 person]* | 1 = None  2 = 1 OR 2  3 = 3 TO 10  4 = More than 10  5 = DK  6 = R |
| 1. **Should public authorities (***for example the government, the state, political stakeholders***) consider homelessness as a priority?** | 1 = YES  2 = NO  3 = DK  4 = R |
| 1. **When passing by a homeless person, are you cautious?** | 1= Often  2= Sometimes  3= Rarely  4= Never  5= DK  6 =R |
| 1. **Over the past year, have you …**   *[read out items]* | 1 = YES  2 = NO  3 = DK  4 = R |
| 1. Given money, food or clothing to a homeless person | 1 2 3 4 |
| 1. Given money, food or clothing to a charitable or non-profit organization for homeless people | 1 2 3 4 |
| 1. Done any volunteer work in a charitable or in non-profit organization for homeless people. | 1 2 3 4 |
| 1. Other*[specify]*: ___________________ | 1 2 3 4 |
| 1. **To reduce homelessness, would you be willing to** *[read out item]***?** | 1 = YES  2 = NO  3 = DK  4 = R |
| 1. Pay more taxes? | 1 2 3 4 |
| 1. Volunteer? | 1 2 3 4 |
| 1. Have a homeless shelter near your home? | 1 2 3 4 |

**Module *Willingness to pay***

*[Note:* ***Never*** *read out DK or R modalities]*

| [Interviewer Script]  Now, we would like to know your opinion on different interventions to house homeless people.  Do you know that in Ireland:  4643 people are homeless; About 1/3 suffer from addictions (alcohol or drugs) and 1/3 suffer from severe mental illness.  Current solutions are either emergency shelters or transitional shelters.  In the first instance, a homeless person is offered emergency accommodation on a night-by-night basis. They may then be offered longer term or transitional accommodation in a congregate hostel setting. Residence in this setting can range from 3 months to 2 years, although many people stay longer. The homeless person must obey rules (e.g., curfews). Often, there is a requirement for sobriety and adherence to mental health treatment plans. Couples and people with pets are generally not allowed. If any of these rules or requirements are violated, eviction is possible. Support workers are available on-site and a financial contribution is requested from the homeless individual on the basis of income.  In the next section, we will describe an innovative housing program for homeless people.  We ask you to tell us how much you would be willing to pay for this program. We do not want you to estimate the actual cost of the program but to say what value you put in the program. All this is hypothetical and only for research purposes. There will be no tax increase!  You may be willing to pay a lot, a little or nothing at all.  There is no right or wrong answer. | | |
| --- | --- | --- |
| **This innovative program is called *Housing First*.**  It is for homeless people with mental or physical health problems (i.e., mental illness, alcohol or drug addiction, disability).  To enter this program, a homeless person doesn’t need to undergo any treatment for mental health disorders or addiction problems.  They have individual housing and benefit from medical and social assistance adapted to their needs (7 days a week).  They pay part of their rent, but in case of insufficient income, subsidies are available.  A similar program is currently being tested in Ireland and shows that after two years, the vast majority of homeless people (about 67%) included in this program stays in their dwelling. | | |
| 1. **I will propose amounts in euros. Please tell me what you would be willing to pay each year through taxes for this program.**   *[Interviewer: if spontaneous answer is “Zero euro” or do not want to pay, report 1 = YES here.*  *Then go to Q16.2]*  *[Interviewer: after the first refusal or “don’t know”* ***stop*** *and continue with the next question Q 16.1].*  **Would you be willing to pay ………………………**  *[If respondent accepts the highest bid (€*400*), go to question Q 16.1].* | 1 = YES  2 = NO  3= DK  4 =R  0€ == > 1  *(Currency/change will be adapt to COUNTRY)*  €10? …… 1 2 3 4 *[83cts/month]*  €25? …….1 2 3 4 *[2€/ month]*  €50? …....1 2 3 4 *[4€// month]*  €100? …...1 2 3 4 *[8€// month]*  €200? …...1 2 3 4 *[17€// month]*  €400? …...1 2 3 4 *[33€// month]* |  |
| 1. **What would be the maximum you would be willing to pay each year through taxes for this program?**   *Remind the respondent of the last amount they accepted (and the amount they refused, where applicable). The goal is to have the respondent indicate a maximum between these 2 values].*  *Ex: if yes to 25 € and not 50 €, ask: Between 25 and 50 € what would be the maximum amount ...* | /-----------/ € |  |
| 1. *[If respondent answered 0€]* **Please, could you tell me the main reason why you refused? Is it because**… | 1 = The program won’t work  2 = Other programs are more important/of higher priority  3 = I do not want to pay more taxes  *(go to Q18)*  4 = I cannot afford to pay more taxes *(go to Q18)*  5 = Other: -------------------------------  6= DK  7 =R |  |
| 1. **So far I have told you about a program for homeless with mental disorders or addictions. Would you be willing to pay (more, the same, or less) each year through taxes if this program were OPEN TO ALL homeless people?** | 1= More *(go to Q17.1)*  2= Same *(go to Q18)*  3= Less *(go to Q17.1)*  4= DK  5 = R |  |
| 1. **Specify the maximum amount you would be willing to pay every year through taxes for this program?** | /----------/ € |  |
| 1. **How confident are you of your answer?** | 1= absolutely sure  2= relatively sure  3= more or less sure  4= relatively unsure  5 = absolutely unsure |  |
| 1. **For data analysis, we need to know if your income is taxable.** | 1 = YES  2= NO  3= DK  4 =R |  |

*[INTERVIEWER’S SCRIPT].* **Thank you for your answers. Now, I’d like to ask you a few questions about the living conditions of homelessness people in** (*Country).*

| 1. **In the last 3 years, would you say the number of homeless people has …** | 1 = Strongly increased  2 = Somewhat increased  3 = Somewhat decreased  4 = Strongly decreased  5 = Stayed the same (spontaneous)  6 = DK  7 = R |
| --- | --- |
| 1. **I will read several statements about homeless people.** *[Read out item]* **Please tell me if you…** | 1 = STRONGLY AGREE  2 = Somewhat AGREE  3 = Somewhat DISAGREE  4 = STRONGLY DISAGREE  5= DK  6 =R |
| 1. Homeless people are the victims of assaults (violence, robbery, threats, and attacks). | 1 2 3 4 5 6 |
| 1. They are discriminated against in hiring | 1 2 3 4 5 6 |
| 1. They eat at least two meals a day. | 1 2 3 4 5 6 |
| 1. They are able to keep in touch with family and friends. | 1 2 3 4 5 6 |
| 1. They have a shorter life expectancy than the general population. | 1 2 3 4 5 6 |
| 1. Many remain homeless by choice | 1 2 3 4 5 6 |
| 1. Most have working skills | 1 2 3 4 5 6 |
| 1. They could look after (keep clean, decorate) a home if they had one | 1 2 3 4 5 6 |
| 1. They have access to paid or unpaid work (volunteering, internship etc.). | 1 2 3 4 5 6 |
| 1. Their main source of income comes from social welfare benefits | 1 2 3 4 5 6 |
| 1. They spend much of their time alone, outside of any social network | 1 2 3 4 5 6 |
| 1. **In your opinion, do services provided............. meet the needs of homeless people:** | 1 = STRONGLY AGREE  2 = Somewhat AGREE  3 = Somewhat DISAGREE  4 = STRONGLY DISAGREE  5= DK  6 =R |
| 1. In hospitals and emergency rooms | 1 2 3 4 5 6 |
| 1. By General Practitioners and outpatient specialists | 1 2 3 4 5 6 |
| 1. In emergency shelters   *[Investigator if question: maximum length of stay:*  *7 days]* | 1 2 3 4 5 6 |
| 1. In transitional shelters   *[Investigator if question: maximum length of stay: 3 to 6 months]* | 1 2 3 4 5 6 |
| 1. In the *Housing First* program better meet the needs of homeless people when compared to transitional shelter services? | 1 2 3 4 5 6 |
| 1. **In your opinion, what are the THREE reasons that best explain why people become homeless?**   *[Note: wait for spontaneous answer and tick the closest answer].* | 1 = job lost/ unemployment period  2 = insufficient income/can’t afford to pay a rent  3 = home destroyed by a catastrophe (fire, floods, etc.)  4 = over-indebted  5 = ill or disabled  6 = addiction (alcohol, drugs or other types of addiction)  7 = break-up, divorced or loss of family member  8 = mental health problems  9 = no access to social welfare benefits or support services  10 = illegal immigration  11 = Own choice  12 = Other 1:___________________________________  13 = Other 2:___________________________________  14 = Other 3:___________________________________  15 = None *(SPONTANEOUS)*  16= DK  17 =R |
| 1. **In your opinion, who should be mainly responsible for providing EMERGENCY SHELTER for homeless people?** | 1 = Government  2 = Non-Governmental Organizations/Charities /  3 = Churches and religious communities  4 = Homeless themselves  5= DK  6 = R |
| 1. **In your opinion, who should be mainly responsible for providing LONG TERM HOUSING for homeless people?** | 1 = Government  2 = Non-Governmental Organizations/Charities /  3 = Churches and religious communities  4 = Homeless themselves  5 = DK  6 = R |
| 1. **Which homeless group should be given priority for a long-term housing program?**   *[Open-ended question, the modalities should not be read out loud.*  *Wait for answer and check the closest answer or if not listed, write the answer under "other"]* | 1 = Families  2 = Women  3 = Young [<30 years old **with no** children]  4 = People with mental disorders  5 = Employed  6 = the elderly  7 = People with disabilities  8 = Unemployed  9 = people with addiction problems with alcohol or drugs  10 = None  11 = Other ___________________________________  12=DK  13=R |
| 1. **In general, do you think the Government spends (too much, enough or too little) on social welfare …** | 1 = Too much  2 = Enough  3 = Too little  4 = DK  5 = R |
| 1. **Do you think that the Government spends (too much, enough or too little) …… to help homeless people?** | 1 = TOO MUCH  2 = ENOUGH  3 = TOO LITTLE *(go to Q30)*  4 = DK  5 = R *(go to Q30)* |
| 1. **Could you be more specific?**   *[Note: wait for spontaneous answer and tick the closest answer]*  **Is it because…** | 1 = Homeless programs are not a priority  2 = Waste of funds because the policies are inefficiency  3 = Waste of funds because homeless people don’t deserve it  4 = DK  5 =R |

***[INTERVIEWER’S SCRIPT]* Socio-demographic characteristics.**

**Finally, I will ask you a few questions to define your socio-demographic profile.**

| 1. *[For the interviewer ONLY, don’t ask to the respondent!]* | 1= male  2=female |
| --- | --- |
| 1. **In what year were you born?**   *[Code with* ***4 digits****. For example: 1953 years is coded 1953, 90 is coded as 1990.*  *[If there is some HESITATION, skip to Age question]* | Year: /---/---/---/---/ |
| - 1. How old are you? | Age: /----------/ years |
| 1. **What is your nationality?** *[write down country]* | /_______________________/ |
| 1. **What is your highest level of education?**   *Interviewer: Spontaneous answer are expected, don’t provide modalities range…* | 1 = Postgraduate degree (Masters, PhD, etc/ 4 years or more of post-secondary education)  2 = Bachelor or equivalent (up to 3 years of post-secondary education)  3 = Higher education (up to 2 years of post-secondary education)  4 = Leaving Certificate  5 = High school with no Leaving Cert  6 =Junior Certificate  7 = Primary school  8 = have not attended school  9 = Other qualification  10 = DK  11 = NA |
| 1. **At present, what is your work situation?**   *[Only one answer is expected]* | 1 = Working Full time (35 hours or more the week)  2 = Working part time (8 to 35 hours per week)  3 = Working part time occasionally (less than 8 hours)  4 = Full time Student  5 = Retired  6 = Unemployed  7 = Not working for other reasons  8 = DK  9 = R |
| 1. **What is the gross annual income of your HOUSHOLD before any deductions (before paying for income or other taxes, healthcare Insurance, Tax etc.?)**   *[Interviewer: wait for a spontaneous answer and tick the appropriate answer; if refusal to provide exact amount, the following Gross annual household income scale should be proposed:]*  *[Interviewer: if respondent ask for additional information, precise:]* **You should include all sources of income including wages, pensions, social welfare benefits, interest on savings, and annuities.** | /----------------------------------------/€  *(Currency/change will be adapt to COUNTRY)*  1= ‘less than 5 000€’  2= between 5 000 & 10 000’€  3= between 10 000 & 15 000’€  4= between 15 000 & 20 000’€  5= between 20 000 & 30 000’€  6= between 30 000 & 40 000’€  7= between 40 000 & 50 000’€  8= between 50 000 & 60 000’€  9= between 60 000 & 70 000’€  10= ’more than 70 000 €  11 = DK  12 = R |
| 1. **What’s your marital status? Are you?** | 1 = married  2 = widowed  3 = separated/divorced  4 = In common-law union (contractual union outside of marriage)  5 = single |
| 1. **Including yourself, how many people altogether live in your household?** | /-------------------/ |
| 1. **How many children do you have?** | /------------------/ |
| 1. **How many dependent children (that is linked to the taxable household income) are there?** | **/------------------/** |
| 1. **What is your Eircodes?**   *[If respondent doesn’t know or does not wish to give the information, ask for* ***closest*** *administrative division such as department (Fr)/ county (Ir)/ province (It)…]* | /-----------------/ |
| 1. County | /-----------------/ |
| 1. **How many operational landlines does your household have** (not including assigned phone numbers that are not used-i.e. only for internet- or business calls)**?** | /------------------/ |
| 1. **How many mobile phones are does your household have** (not including those used for business calls)? | /------------------/ |
| 1. Do you have a mobile phone | 1 = Yes  2 = No  3 = R |

**END OF INTERVIEW**

**Now, we're done with the interview!**

**The University of (***adapt to* *COUNTRY***) thanks you for participating.**

**Do you have any questions?** *[If yes, the interviewer should make every effort to try to answer].*

*[If the interviewer cannot answer or is unsure]* ***I’m sorry. I do not have this information. However, you can send an email at*** [***HOME_EU@ispa.pt***](mailto:HOME_EU@ispa.pt)

*[Interviewer’s instruction: PLEASE make a note of the question for the next training session.]*

*Questions: ------------------------------------------------------------------------------------------------------------------------------------------------------------------------------------------------------------------------------------------------------------------*

**Also, if you have any questions or comments later, you may call the project director, Dr. Rachel Manning**, **at** **University** of Limerick.

**The phone number is:** 061-234846 or Rachel.manning@ul.ie.

**A short report of the results of this survey once it is completed will be available at the following address: www.home-eu.org**

**Remember, your participation is anonymous. Thank you again. Have a nice day.**

| 1. *INTERVIEWER QUESTION: Did the respondent answer most of the questions in a reasonable manner? Were there any problems that may make the data obtained invalid or questionable?* | 1 = NO PROBLEMS  2 = POTENTIAL PROBLEMS (describe below): _________________________________________________  _________________________________________________  3 = SERIOUS PROBLEMS (describe below): _________________________________________________  _________________________________________________ |  |
| --- | --- | --- |

*INTERVIEWER NOTE: PLEASE GO BACK AND REVIEW TO MAKE SURE ITEMS ARE COMPLETE AND SCORED CORRECTLY. IF NECESSARY, CALL THE RESPONDENT BACK TO CLARIFY ANY PROBLEMS.*

ENQUÊTE TELEPHONIQUE

SUR LES OPINIONS ET PREFERENCES

DES CITOYENS SUR LE SANS-ABRISME

NUMERO D’IDENTIFIANT: /__/__/__/__/__/

DATE: (____ / ____ / ____)

HEURE DEBUT ENTRETIEN : (____: ____) HEURE FIN ENTRETIEN : (____: ____)

ENQUETEUR : _________________________ CODE ENQUETEUR __________

***I. INTRODUCTION***

*[SCRIPT DE L’ENQUETEUR]*

**Bonjour, mon nom est -----------------------------. J'appelle de l’Université Aix Marseille***.* **Votre foyer a été choisi au hasard pour participer à une enquête européenne sur les opinions de la population française au sujet du sans abrisme.**

**Avez-vous 18 ans ou plus ?**

*[Si oui, allez à la section II DISPONIBILITE]*

*[Si non, demandez] :* **Pourrais-je parler à un membre de votre foyer âgé de 18 ans ou plus?**

[*Si oui,* *Une fois que la personne est au téléphone, continuez :]*

**Bonjour, mon nom est -----------------------------. J'appelle de l’Université Aix Marseille. Votre foyer a été choisi au hasard pour participer à une enquête européenne sur les opinions de la population française au sujet du sans abrisme.** *(puis allez à la section II DISPONIBILITE)*

*[Si non, aucun adulte du foyer présent, demandez :]* **Quel est le meilleur moment pour rappeler cette personne?** [*notez DATE/HEURE*] : DATE : ________, HEURE : _______ PRENOM (seulement) ________. **Merci. Nous la rappellerons.**

[*Si non ou ne sait pas*] **Merci beaucoup. Nous essayerons une autre fois.**

***II DISPONIBILITE POUR L’ENQUETE***

**Est-ce un moment convenable pour vous parler pendant environ 20 minutes?**

*[Si oui]* **Merci.** *(puis allez à la section* *V* *CONTEXTUALISER L’ENQUETE)*

*[Si non,*  *demandez :]* **Y at-il un meilleur moment pour vous rappeler?**

- [*Si oui, notez Date/Heure*] : DATE :________, HEURE : ________. **Merci. Nous vous rappellerons.**
- [*Si non ou ne sait pas*]. **Nous souhaiterions vraiment connaître votre opinion sur ce sujet. Etes-vous certain de ne pas pouvoir me consacrer 20 minutes de votre temps maintenant ou à un autre moment qui vous convienne mieux ?**
- *[Si oui, veut participer maintenant]* **Merci beaucoup !** *(puis allez à la section* *V CONTEXTUALISER L’ENQUETE)*
- [*Si oui une autre fois, notez Date/Heure*] : DATE :________, HEURE : ________. **Merci. Nous vous rappellerons.**
- *[Si non, allez à la section III REFUS]*

***III. REFUS***

1. **Nous comprenons bien que vous ne souhaitez pas participer à l’enquête; Pourriez-vous cependant nous aider à analyser les refus en nous donnant votre âge, votre diplôme le plus élevé et votre statut professionnel?**

1 = Oui

2 = Non

1. **Année de naissance /---/---/---/---/**
2. **Niveau d’éducation le plus élevé**

1 = Master/thèse (BAC+4 ou plus)

2 = Licence (BAC+3)

3 = Etudes supérieures (BAC + 2)

4 = BAC

5 = Lycée

6 = Collège

7= Primaire

8= Non scolarisé(e)

9 = Autre/Diplôme étranger

10 = NSP

11 = NVPR

1. **Statut professionnel**

1 = Plein temps (35 heures ou plus per semaine)

2 = Temps partiel (8 à 32 heures par semaine)

3 = Temps partiel occasionnel (< 8 heures/semaine)

4 = Etudiant

5 = Retraité

6 = Chômeur

7 = Sans activité pour autre raison

8 = NSP

9 = NVPR

1. **Combien de personnes vivent dans votre foyer, y compris vous-même? _______**
2. **Combien d'enfants (de moins de 18 ans) vivent dans votre foyer ? ________**

**Merci pour votre aide et désolé(e) de vous avoir dérangé.**

**IV. LE RÉPONDANT EST UN ÉTRANGER**

*[Note: Si vous parlez à quelqu'un qui parle une langue étrangère (et dont le français est évidemment insuffisant pour terminer l'entrevue), dites:]*

**Je suis désolé; Je parle seulement français. Merci pour votre temps, au revoir.**

***V. CONTEXTUALISER L’ENQUETE***

*[SCRIPT DE L’ENQUETEUR]* **Cette enquête téléphonique fait partie d'une grande enquête menée dans 8 pays européens. L’objectif est de mesurer l’opinion des citoyens sur la question du sans-abrisme et de comprendre leurs attentes sur cette question. Sachez qu’au cours de cet entretien seule votre opinion concernant la situation en France sera abordée. Vos réponses resteront strictement anonymes. Je ne vous demanderai ni votre nom ni votre prénom. Si vous ne souhaitez pas répondre à certaines questions, vous pouvez simplement me le dire.**

**Etes-vous d'accord pour participer à cette enquête sur les opinions des citoyens sur le sans-abrisme?**

OUI NON *[allez à la section III REFUS]*

*[Si OUI]* **Très bien. Nous pouvons alors commencer!**

*[SCRIPT DE L’ENQUETEUR]* **Dans un premier temps, je vais vous poser des questions d’ordre général sur les Sans Domicile Fixes/SDFs. Tout d’abord, sachez que nous définissons le sans-abrisme comme le fait de dormir dans la rue, dans une voiture, ou vivre dans un foyer d’hébergement d’urgence ou temporaire.**

| *[Note : Ne* ***jamais*** *lire les modalités NSP et NVPR]* | NSP = Ne sais pas  NVPR = Ne veut pas répondre |
| --- | --- |
|  |  |
| 1. **Avez-vous déjà été SDF ?**   *[Enquêteur : même pour une nuit, compter OUI]* | 1 = OUI  2 = NON *(allez à Q4)*  3 = NVPR (*allez à Q4*) |
| 1. *[Si «oui» à Q1]:* **C’était quand la dernière fois?** *[lire les items]* | 1 = Au cours des 12 derniers mois  2 = Il y a 1 ou 2 ans  3 = Il y a 3 ou 4 ans  4 = Il y a 4 ou 5 ans  5 = Il y a plus de 5 ans  6 = NSP  7= NVPR |
| 1. *[Si «oui» à Q1]:* **Au cours de votre vie, combien de temps avez-vous été SDF?** *[énoncez]* | 1 = Moins d’une semaine  2 = Moins d’un mois  3 = Moins d’une année  4 = Moins de 2 années  5 = Moins de 4 années  6 = Plus de 4 années  7 = NSP  8 = NVPR |
| 1. **Y a t-il eu des membres de votre famille, des amis ou des connaissances ayant été SDF?** | 1 = OUI  2 = NON *(allez à Q6)*  3 = NSP (*allez à Q6)*  4 = NVPR (*allez à Q6)* |
| 1. *[Si famille ou ami sans abri = oui]* **Était-ce ...** | 1 = Parent *[père, mère]*  2 = Enfant  3 = Frère/sœur  4 = Mari/épouse/partenaire  5 = Ami  6 = Autre membre de la famille  7 = Une connaissance  8 = NSP  9 = NVPR |
| 1. **Pourriez-vous me dire approximativement combien y a-t-il de SDFs en France?**   *[Le répondant doit fournir un nombre - pas un pourcentage]* | _______________ |
| 1. **A votre avis quel est le pourcentage de SDFs**   *[Le répondant doit fournir un pourcentage]* |  |
| 1. avec troubles mentaux? | _______% |
| 1. avec des problèmes de dépendances (à l’alcool ou drogues)? | _______% |
| 1. **Qui selon-vous finance principalement les structures sociales pour les SDFs en France?**   *[Une seule réponse possible]* | 1 = Gouvernement  2 = Organisations non gouvernementales/ de charité  3 = Eglise ou autre communauté religieuse  4 = NSP  5 = NVPR |
| 1. **Qui selon-vous finance principalement les structures sanitaires pour les SDFs en France ?**   *[Une seule réponse possible]* | 1 = Gouvernement  2 = Organisations non gouvernementales/ de charité  3 = Eglise ou autre communauté religieuse  4 = NSP  5 = NVPR |
| 1. **Là où vous vivez, diriez-vous qu'il y en a (beaucoup de personnes, quelques personnes, peu de personnes ou aucune personne) SDF?**   *[là où vous vivez = le quartier]* | 1 = Beaucoup de personnes  2 = Quelques personnes  3 = Peu de personnes  4 = Aucune *(allez à Q12)*  5 = NSP  6 = NVPR |
| 1. **Combien de SDFs différents voyez-vous en moyenne par semaine?**   *[Si le répondant voit la même personne plusieurs fois par semaine, il faut compter une seule personne]* | 1 = Aucun  2 = 1 ou 2  3 = 3 à 10  4 = Plus de 10  5 = NSP  6 = NVPR |
| 1. **Pensez-vous que les pouvoirs publics devraient considérer le sans-abrisme comme une priorité?** | 1 = Oui  2= Non  5 = NSP  6 = NVPR |
| 1. **Lorsque vous passez près d'une personne SDF, êtes-vous prudent?** | 1= Souvent  2= Parfois  3= Rarement  4= Jamais  5 = NSP  6 = NVPR |
| 1. **Au cours de l'année précédente, avez-vous …**   *[lire les items]* | 1 = OUI  2 = NON  3 = NSP  4 = NVPR |
| 1. Donné de l'argent, de la nourriture, des vêtements à une personne sans abri ? | 1 2 3 4 |
| 1. Donné de l'argent, de la nourriture, des vêtements à un organisme ou association pour les sans-abris ? | 1 2 3 4 |
| 1. Travaillé de façon bénévole dans un organisme ou une association pour les sans-abris ? | 1 2 3 4 |
| 1. Autre *[précisez]:* __________________________ | 1 2 3 4 |
| 1. **Pour réduire le sans-abrisme, seriez-vous prêt à** *[lire proposition] ?* | 1 = OUI  2 = NON  3 = NSP  4 = NVPR |
| 1. Payer plus d'impôts? | 1 2 3 4 |
| 1. Faire du bénévolat? | 1 2 3 4 |
| 1. Accepter un foyer pour les sans-abris situé près de chez vous? | 1 2 3 4 |

**Module *Disposition à payer***

*[Note : Ne* ***jamais*** *lire les modalités NSP et NVPR]*

| [Script enquêteur]  Maintenant, nous aimerions connaître votre opinion sur différentes interventions pour loger les sans-abri.  Savez-vous qu’en France :  140 000 personnes sont sans abri; environ 1/3 souffrent de dépendances (à l’alcool ou aux drogues) et 25% souffrent de maladies mentales sévères.  Les solutions actuelles sont soit des hébergements d'urgence, soit des hébergements temporaires.  Un sans-abri peut rester jusqu’à une semaine dans un hébergement d'urgence. Après plusieurs mois d'attente, il peut accéder à un hébergement temporaire, où il dort souvent dans des dortoirs. Il est autorisé à y rester 3 à 6 mois, s'il respecte certaines règles, comme les couvre-feux et s’il n'a aucun problème de santé mentale ou de dépendances à l'alcool à l'entrée. Des travailleurs sociaux sont également disponibles. Les couples et les personnes avec des animaux de compagnie ne sont pas admis. Une contribution financière est demandée sur la base du revenu.  Dans la section suivante, nous vous présentons un programme innovant de logement pour les SDFs.  Votre objectif est de nous dire combien vous seriez prêt à payer pour ce programme. Nous ne voulons pas que vous estimiez le coût réel du programme mais que vous exprimiez la valeur/l’utilité que vous associez au programme. Tout cela est hypothétique et uniquement à des fins de recherche. Il n'y aura pas d'augmentation d’impôts!  Vous pouvez être disposé à payer beaucoup, un peu ou rien du tout.  Il n'y a pas de réponse juste ou fausse. | |
| --- | --- |
| **Ce programme innovant s’appelle *Un chez Soi d’Abord.***  Il s'adresse aux personnes sans-abris souffrant de troubles de la santé mentale ou physique (à savoir maladie mentale, dépendance à l’alcool ou aux drogues, handicaps).  Aucune obligation de traitement psychotrope ou de sevrage ne conditionne l’accès à ce programme.  Ils ont un logement individuel et bénéficient d’une assistance médicale et sociale adaptée à leurs besoins (pouvant aller jusqu’à 7 jours sur 7).  Ils paient une partie de leur loyer, mais en cas de revenus insuffisants, des subventions sont disponibles.  Un programme similaire est actuellement expérimenté en France et montre qu'après deux ans, la grande majorité des SDFs (environ 90%) inclus dans ce programme se maintiennent dans leur logement. | |
| 1. **Je vais vous proposer des montants en euros. Dîtes-moi ce que vous seriez prêt à payer chaque année via l’impôt pour ce programme.**   *[Si la réponse spontanée est «Zéro euro» ou qu'il ne veut pas payer, entourez 1 = OUI ici. Puis passez ensuite à Q16.2]*  *[Après le premier NON ou NSP,* ***arrêtez-vous****, et passer à la question Q 16.1]*  **Seriez-vous prêt à payer** ………………………  *[Si le répondant dit OUI à l'offre la plus élevée (€ 400), passer à la question Q16.1:]* | 1 = OUI  2 = NON  3 = NSP  4 = NVPR  0€ == > 1  €10? …… 1 2 3 4 *[83cts/mois]*  €25? …….1 2 3 4 *[2 €/mois]*  €50 ? …....1 2 3 4 *[4€//mois]*  €100? …...1 2 3 4 *[8€//mois]*  €200? …...1 2 3 4 *[17€//mois]*  €400? …...1 2 3 4 *[33€//mois]* |
| 1. **Quel serait le montant maximal que vous seriez prêt à payer par an via l’impôt pour ce programme?**   *[Rappelez le dernier montant accepté (et le montant refusé si c’est le cas). Le but est d’amener le répondant un montant maximal entre ces 2 valeurs]. Ex : si oui à 25€ et non à 50€, demander : Entre 25 et 50€ quel serait le montant maximal…* | /-----------/ €  *(Puis allez Q17)* |
| 1. *[Si réponse = 0€]* **Pouvez-vous me dire la raison principale de votre refus? Est-ce parce que ...**   *[Puis passez à la Q 26]* | 1 = Le programme ne fonctionnera pas  2 = D'autres programmes sont plus importants/ prioritaires  3 = Vous ne souhaitez pas payer plus d'impôts *(allez Q18)*  4 = Vous ne pouvez pas vous permettre de payer plus d'impôts *(allez Q18)*  5 = Autre: ----------------------------  6 = NSP  7 = NVPR |
| 1. **Jusqu’à présent je vous ai parlé d’un programme pour les SDFs avec troubles mentaux ou addictions. Seriez-vous disposé à payer (plus, autant, ou moins) chaque année via l’impôt si ce programme était OUVERT A TOUS LES SDFs ?** | 1 = Plus *(allez Q17.1)*  2 = Autant *(allez Q18)*  3 = Moins *(allez Q17.1)*  4 = NSP  5 = NVPR |
| 1. **Précisez le montant maximal que vous seriez prêt à payer par an via l’impôt pour ce programme?** | /-------/ € |
| 1. **À quel point êtes-vous certain de vos réponses à cet exercice?** | 1 = Tout à fait certain  2 = Plutôt certain  3 = Plutôt pas certain  4 = Pas du tout certain  5 = NSP  6 = NVPR |
| 1. **Pour l’analyse des données, j’ai besoin de savoir si vous êtes imposable.** | 1 = OUI  2 = NON  3 = NSP  4 = NVPR |

[*SCRIPT enquêteur*] **Merci pour vos réponses. Maintenant, je vais vous poser des questions sur les conditions de vie des SDFs en France**

*[Note : Ne* ***jamais*** *lire les modalités NSP et NVPR]*

| 1. **Au cours des 3 dernières années, diriez-vous que le nombre de SDFs a …** | 1 = Fortement augmenté  2 = Plutôt augmenté  3 = Plutôt diminué  4 = Fortement diminué  5 = Est resté le même (spontané)  6 = NSP  7 = NVPR |
| --- | --- |
| 1. **Je vais vous lire plusieurs affirmations sur les SDFs. (***Lire chaque item***) Dites-moi si vous êtes…. :** | 1=Tout à fait d'accord  2=Plutôt d'accord  3=Plutôt pas d’accord  4=Pas du tout d’accord  5=NSP  6= NVPR |
| 1. Ils sont victimes d'agressions (violence, vol, menaces et attaques). | 1 2 3 4 5 6 |
| 1. Ils sont victimes de discrimination à l'embauche | 1 2 3 4 5 6 |
| 1. Ils mangent au moins deux repas par jour. | 1 2 3 4 5 6 |
| 1. Ils sont capables de garder des contacts avec des amis ou des parents | 1 2 3 4 5 6 |
| 1. Ils ont une espérance de vie plus courte que la population générale. | 1 2 3 4 5 6 |
| 1. Beaucoup restent sans abri par choix | 1 2 3 4 5 6 |
| 1. Ils ont des compétences professionnelles | 1 2 3 4 5 6 |
| 1. Ils pourraient s’occuper (maintenir propre, aménager) d’un logement s’ils en avaient un | 1 2 3 4 5 6 |
| 1. Ils ont accès à une activité rémunérée ou non (emploi, bénévolat, etc.) | 1 2 3 4 5 6 |
| 1. Leurs principales sources de revenus sont les prestations/aides sociales | 1 2 3 4 5 6 |
| 1. Ils passent une grande partie de leur temps seul, en dehors de tout réseau social | 1 2 3 4 5 6 |
| 1. **Selon vous, est ce que l’offre …………. répond aux besoins des SDFs :** | 1=Tout à fait d'accord  2=Plutôt d'accord  3=Plutôt pas d’accord  4=Pas du tout d’accord  5=NSP  6= NVPR |
| 1. De soins hospitaliers et des services d’urgences | 1 2 3 4 5 6 |
| 1. De soins en médecine de ville | 1 2 3 4 5 6 |
| 1. Des services d’hébergement d'urgence   *[Enquêteur si question : durée maximale séjour 7 jours]* | 1 2 3 4 5 6 |
| 1. Des **s**ervices d’hébergement temporaire   *[Enquêteur si question : durée séjour 3 à 6 mois]* | 1 2 3 4 5 6 |
| 1. Du programme *Un chez soi d’abord* comparé aux services d’hébergement temporaire répond mieux aux besoins des SDFs ? | 1 2 3 4 5 6 |
| 1. **Selon vous, quelles sont les trois raisons qui expliquent le mieux pourquoi les gens deviennent SDFs ?**   *[Remarque: attendre les réponses spontanées et cocher la réponse la plus proche]* | 1 = perte emploi/période de chômage  2 = revenus insuffisants/ pas possible de payer un loyer  3 = logement détruit par une catastrophe (incendie, inondation, etc.)  4 = le surendettement  5 = maladie ou handicap  6 = les problèmes de dépendance (alcool, drogues ou autres)  7 = séparation ou perte d’un parent proche  8 = problèmes de santé mentale  9 = pas accès à des prestations sociales ou à des services d’aide adaptés  10 = immigration illégale  11 = choix de vie  12 =autre 1:_________________  13 =autre 2:_________________  14 =autre 3:_________________  15 = aucune (spontané)  16 = NSP  17 = NVPR |
| 1. **A votre avis, qui devrait être principalement responsable de L’HEBERGEMENT D’URGENCE pour les SDFs.** | 1 = Gouvernement  2 = Organisations non gouvernementales/associations caritatives  3 = Eglises ou autres communautés religieuses  4 = SDFs eux-mêmes  5 = NSP   1. = NVPR |
| 1. **A votre avis, qui devrait être principalement responsable du logement à long terme des SDFs.** | 1 = Gouvernement  2 = Organisations non gouvernementales/associations caritatives  3 = Eglises ou autres communautés religieuses  4 = SDFs eux-mêmes  5 = NSP  6 = NVPR |
| 1. **Quelle population parmi les SDFs devrait être prioritaire pour un programme de Logement à long terme?**   *[Question ouverte, les modalités de réponse ne doivent pas être dites.*  *Attendre la réponse et cocher la réponse la plus proche ou si ne figure pas dans la liste, renseigner la réponse dans autre]* | 1 = les Familles  2 = les Femmes  3 = les Jeunes *[<30 ans* ***sans*** *enfant(s)]*  4 = les personnes avec des troubles mentaux  5 = les personnes avec un travail  6 = les personnes âgées  7 = les personnes handicapées  8 = les personnes sans travail  9 = les personnes avec des problèmes d’addiction à l’alcool ou la drogue  10 = Aucune  11= Autre __________________  12 = NSP  13 = NVPR |
| 1. **En général, pensez-vous que le Gouvernement dépense (trop, suffisamment, pas assez) en prestations/aides sociales ?** | 1 = Trop élevées  2 = Suffisamment élevées  3 = Pas assez élevées  4 = NSP  5 = NVPR |
| 1. **Pensez-vous que le Gouvernement dépense (trop, suffisamment, pas assez) pour aider les SDFs?** | 1 = Trop  2 = Suffisamment  3 = Pas assez (*allez Q31)*  4 = NSP  5= NVPR (*allez Q31)* |
| 1. *[Si réponse Q29 « Trop » ou* « *Suffisamment »]* **Pouvez-vous préciser votre réponse?**   *[Remarque: attendre une réponse spontanée et cocher la réponse qui s'y rapproche le plus]*  **Est-ce parce que…** | 1 = Ce n’est pas une priorité  2 = C’est un gaspillage parce que inefficace  3 = C’est un gaspillage parce que les sans-abris ne le méritent pas  4 = le gouvernement dépense un montant « adéquat » pour les personnes sans abris  5 = Autre  6 = NSP  7 = NVPR |

***[SCRIPT ENQUETEUR] Pour finir, je vais vous poser quelques questions* pour définir votre profil socio-démographique**

| 1. [*Pour l’enquêteur SEULEMENT, ne pas demander au répondant*!] | 1= Homme  2 =Femme |
| --- | --- |
| 1. **En quelle année êtes-vous né?**   *[Code à 4 chiffres. Par exemple: 1953 ans est marqué 1953, 90 est codé comme 1990.]*  *[S'il y a une HESITATION, passez à la question Age]* | Année: /---/---/---/---/ |
| 1. Quel âge avez-vous? | Age: /----------/ année |
| 1. **Quelle est votre nationalité ?** [noter le pays] | /_________________________ / |
| 1. **Quel est votre plus haut niveau d'éducation?**   *[Enquêteur: Une réponse spontanée est attendue, ne pas lire les modalités ...]* | 1 = Master/thèse (BAC+4 ou plus)  2 = Licence (BAC+3)  3 = Etudes supérieures (BAC + 2)  4 = BAC  5 = Lycée  6 = Collège  7 = Primaire  8 = Non scolarisé(e)  9 = Autre/ Diplôme étranger  10 = NSP  11 = NVPR |
| 1. **À l'heure actuelle, quelle est votre situation professionnelle?**   *[Enquêteur : Une seule réponse possible]* **Vous travaillez… :** | 1 = Plein temps (35 heures ou plus per semaine)  2 = Temps partiel (8 à 32 heures par semaine)  3 = Temps partiel occasionnel (< 8 heures/semaine)  4 = Etudiant  5 = Retraité  6 = Chômeur  7 = Sans activité pour autre raison  8 = NSP  9 = NVPR |
| 1. **Quel est le revenu brut annuel de votre MÉNAGE avant toute déduction (avant impôts et autres cotisations telles que CSG, assurance maladie, etc)?**   *[Enquêteur : Préférez une réponse spontanée et cochez la tranche de revenu correspondante ; Si le répondant refuse de donner un montant exact, proposer les tranches de revenus :]*  *[Enquêteur : Si le répondant veut des précisions, précisez :]* Vous devez inclure toutes vos sources de revenus, y compris les salaires, les pensions, les avantages sociaux, les intérêts sur l'épargne et les rentes. | /------------------------------/€  1= ‘moins de 5 000€’  2= entre 5 000 et 10 000’€  3= entre 10 000 et 15 000’€  4= entre 15 000 et 20 000’€  5= entre 20 000 et 30 000’€  6= entre 30 000 et 40 000’€  7= entre 40 000 et 50 000’€  8= entre 50 000 et 60 000’€  9= entre 60 000 et 70 000’€  10= ‘plus de 70 000 €  11=NSP  12=NVPR |
| 1. **Quel est votre statut matrimonial?** | 1 = Marrié(e)  2 = Veuf(ve)  3 = Séparé(e)/divorcé(e)  4 = En concubinage/PACS  5 = Célibataire |
| 1. **En vous comptant, combien de personnes vivent au sein de votre foyer ?** | /-----------/ |
| 1. **Combien avez-vous d’enfants?** | /-----------/ |
| 1. **Combien d’enfants sont rattachés au foyer fiscal ?** | /-----------/ |
| 1. **Quel est votre code postal?**   *[Demandez le département si ne sait pas son code postal]* | /---------/ |
| - 1. Département | ____________ |
| **40.bis Diriez-vous que vous habitez… ?** | 1 = en milieu urbain  2 = en milieu semi-urbain  3 = en milieu rural  4 = NSP  5 = NVPR |
| 1. **Combien de lignes téléphoniques fixes fonctionnelles dispose votre foyer** (sans inclure les lignes attribuées mais non utilisées ni les lignes professionnelles)**?** | /---------/ |
| 1. **Combien de lignes téléphoniques mobiles dispose votre foyer?** | /---------/ |
| - 1. Avez-vous personnellement un téléphone mobile ? | 1= OUI  2= NON  3= NVPR |

**FIN DE L’ENTRETIEN**

**Maintenant, l’entretien est terminé!**

**L’université Aix Marseille vous remercie de votre participation.**

**Avez des questions ?** [*Si oui, l’enquêteur doit faire de son mieux pour y répondre].*

*[Si l’enquêteur ne peut y répondre ou est incertain].* **Désolé, je n’ai pas cette information. Cependant vous pouvez envoyer un mail à** [**HOME_EU@ispa.pt**](mailto:HOME_EU@ispa.pt)**.**

*[Note enquêteur : Veuillez prendre en note de la question pour la prochaine session de formation].* Question : ____________________________________________________________________________

**Si vous avez des questions ou des commentaires plus tard, vous pouvez appeler le responsable de l’enquête, le Pr Pascal AUQUIER, à l’Université AIX MARSEILLE.**

**Le numéro de téléphone est:** 04 91 32 45 22.

**Un bref rapport des résultats de cette enquête sera disponible sur le site :** [**www.HOME-EU.ORG**](http://www.HOME-EU.ORG)**.**

**Je vous rappelle que votre participation à cette enquête est tout à fait anonyme.**

**Merci infiniment ! Bonne journée !**

| 1. **QUESTION** *POUR ENQUETEUR: Selon vous, est-ce que la plupart des réponses données sont fiables ?* | 1. = Aucun de problème 2. = Légers Problèmes *[précisez]* :   ______________________________________________   1. = Problèmes sérieux  *[Renseignez les questions qui ont posées problème]*: __________________________________________________________________________________   ______________________________________________ |
| --- | --- |

*NOTE: Veuillez relire le questionnaire et pour vous assurer que tous les items sont bien remplis. Et si nécessaire, rappelez la personne pour préciser certaines réponses.*

| VRAGENLIJST VOOR BURGERS OVER  DE PUBLIEKE OPINIE  OVER DAKLOOSHEID EN VOORKEUREN |
| --- |
| IDENTIFICATIENUMMER: /__/__/__/__/__/  DATUM: (____/____/____)  STARTTIJD INTERVIEW : (____:____) EINDTIJD INTERVIEW: (____:____)  INTERVIEWER : _________________________ INTERVIEWERCODE : __________ |

1. ***INTRODUCTIE***

*[INTERVIEWER’S SCRIPT]*

**Hallo, mijn naam is ----------------------------------. Ik bel namens het Radboudumc. Uw huishouden is willekeurig geselecteerd om deel te nemen aan een Europees onderzoek naar de (***NEDERLANDSE***) opinie over dakloosheid.**

**Bent u 18 jaar of ouder?**

*[Zo ja, ga naar deel II: BESCHIKBAARHEID VOOR VRAGENLIJST]*

*[Zo nee, vraag]:* **Kan ik nu iemand spreken in uw huishouden die 18 jaar of ouder is?**

1. *[Zo ja, wanneer die persoon aan de telefoon komt, vervolg met het volgende: ]:* **Hallo mijn naam is ----------------------------------. Ik bel namens het Radboudumc. Uw huishouden is willekeurig geselecteerd om deel te nemen aan een Europees onderzoek naar de publieke opinie over dakloosheid.** *[en ga naar deel II BESCHIKBAARHEID VOOR VRAGENLIJST]*
2. *[Zo nee, volwassene is niet thuis, vraag dan:*] **Wanneer zou een goed tijdstip zijn om deze persoon thuis te bereiken?**

[NOTEER VOOR TERUGBELLEN]: Datum: _______ Tijd: ________Naam (Alleen voornaam) ________ **Dank u wel, We zullen dan terugbellen.**

1. [Bij ‘weet niet’ of nee:]: **Bedankt. We proberen het een andere keer opnieuw.**
2. ***BESCHIKBAARHEID VOOR VRAGENLIJST***

**Kunnen we ongeveer 20 minuten praten?**

1. [*Zo ja]:* **Bedankt!** *(en ga naar deel V* *HET INTERVIEW STARTEN)*
2. *[Zo nee, vraag*:] **Wanneer zou een gunstige tijd zijn om terug te bellen?**

[*NOTEER VOOR TERUGBELLEN*:] datum:__________ tijd: _______________ **Dank u wel. We zullen dan terugbellen.**

- - *[Zo nee of weet het niet, zeg*:] **We zouden heel graag uw kijk op dit onderwerp willen. Weet u zeker dat we niet nu of op een ander tijdstip kunnen praten?**
    - *[Zo ja].* **Bedankt!** *(ga naar deel V* *HET INTERVIEW STARTEN]*
    - [*Zo ja maar ander tijdstip, noteer datum en tijd:]*

datum:_______________ tijd: _______________ **Dank u wel. We bellen u dan terug.**

- - - *[ZO nee, ga naar deel III* WEIGERING*].*

1. ***WEIGERING***
   1. **We begrijpen dat u niet wilt deelnemen; maar zou u ons willen helpen met het analyseren van afwijzingen door uw leeftijd, hoogste afgeronde opleiding en uw beroepsstatus te vertellen?**

1 = Ja

2 = Nee

- 1. **Geboortejaar: ____________________**
  2. **Hoogst genoten opleiding :**

1 = Postuniversitaire diploma (Master, PhD, etc/ 4 of meer jaar vervolgonderwijs)

2 = Bachelor of vergelijkbaar (tot 3 jaar vervolgonderwijs)

3 = Hoger onderwijs (tot 2 jaar vervolgonderwijs)

4 = [Eindexamen](http://context.reverso.net/traduction/neerlandais-francais/eindexamen) (VWO, VMBO, HAVO)

5 = Middelbare school afgerond

6 = Middelbare school

7 = Basisschool

8 = Niet naar school geweest

9 = Andere kwalificaties

10= DK

11= NA

- 1. **Beroepsstatus:**

1 = voltijd werk (35 uur per week of meer)

2 = deeltijd werk (8 to 35 uur per week)

3 = af en toe deeltijd werk (minder dan 8 uur)

4 = Voltijd student

5 = gepensioneerd

6 = werkloos

7 = geen werk vanwege andere redenen

8 = DK

9 = NA

- 1. **Hoeveel mensen wonen er in uw huishouden, inclusief uzelf? ____**
  2. **Hoeveel kinderen onder de 18 jaar wonen in uw huishouden? ______**

**Bedankt voor uw hulp en excuses voor het storen.**

1. ***De deelnemer spreekt Onvoldoende (Nederlands)***

*[Let op: Als de persoon die je bereikt hebt een andere taal spreekt (en wiens Nederlands duidelijk niet voldoende is om het interview af te nemen), zeg:****]***
***Het spijt me, ik spreek alleen Nederlands. Bedankt voor uw tijd en tot ziens.***

1. ***HET INTERVIEW STARTEN***

*[INTERVIEWER’S SCRIPT]*

**Deze telefonische vragenlijst is onderdeel van een groot vragenlijstonderzoek, dat uitgevoerd wordt in 8 Europese landen. Het doel is om de meningen en verwachtingen van burgers over dakloosheid te meten en te begrijpen. Tijdens dit interview zal alleen uw mening over de situatie in Nederland aan de orde komen. Uw antwoorden blijven strikt anoniem. Ik zal u niet om uw naam of voornaam vragen. Als u sommige vragen niet wilt beantwoorden, dan kunt u dat gewoon zeggen.**

**Gaat u akkoord met deelname aan deze vragenlijst dat de meningen van burgers over dakloosheid meet?**  Ja

Nee *(Ga naar Deel III WEIGERING)*

*[Zo ja]* **Goed dan, laten we nu beginnen!**

*[INTERVIEWER’S SCRIPT]* **Ik zal beginnen algemene vragen te stellen over daklozen. Allereerst moet u weten dat wij dakloosheid definiëren als slapen op straat of in een auto, of verblijven in de noodopvang of tijdelijke opvang.**

| *[Let op : Lees DK of R opties* ***nooit*** *hardop voor]* | DK= Weet niet  R= Weigert te antwoorden |
| --- | --- |
|  |  |
| 1. **Bent u ooit dakloos geweest?**   *[let op : Zelfs één nacht telt als JA]* | 1 = JA  2 = Nee *(ga naar Q4)*  3 = R(*ga naar Q4*) |
| 1. *[Als antwoord op Q1 = JA]:* **Wanneer was dit?** *[Lees antwoordmogelijkheden voor]* | 1 = in de afgelopen 12 maanden  2 = 1-2 jaar geleden  3 = 3-4 jaar geleden  4 = 4-5 jaar geleden  5 = Meer dan 5 jaar geleden  6 = DK  7 = R |
| 1. *[Als antwoord op Q1 = Ja]:* **Hoe lang bent u in totaal dakloos geweest gedurende uw leven?** *[Lees antwoordmogelijkheden voor]* | 1 = Minder dan een week  2 = Minder dan een maand  3 = Minder dan een jaar  4 = minder dan twee jaar  5 = minder dan drie jaar  6 = minder dan vier jaar  7 = DK  8= R |
| 1. **Is een familielid, vriend of kennis van u ooit dakloos geweest?** | 1 = JA  2 = Nee *(ga naar Q6)*  3 = DK (*ga naar Q6)*  4 = R (*ga naar Q6)* |
| 1. *[Als familielid of vriend ooit dakloos was (Q4=JA)]* **Was dit u/een..?** | 1 = ouder *[vader, moeder]*  2 = Kind  3 = broer/zus  4 = Echtgenoot/echtgenote/partner  5 = vriend  6 = ander familielid  7 = Kennis  8 = DK  9 = R |
| 1. **Kunt u mij vertellen hoeveel daklozen er ongeveer zijn in (Nederland)?**   *[De deelnemer moet een aantal noemen – geen percentage]* | /_______________/ |
| 1. **Wat is volgens u het percentage daklozen met…**   *[De deelnemer moet een percentage noemen]* |  |
| 1. Psychische stoornissen? | _______% |
| 1. Verslavingsproblemen (alcohol, drugs)? | _______% |
| 1. **Wie financiert in *(Nederland)* de meeste sociale voorzieningen voor daklozen?**   *[Slechts een antwoord]* | 1 = overheid  2 = niet-gouvermentele organisaties/ liefdadigheidsinstellingen  3 = Kerken en religieuze gemeenschappen  4 = DK  5 = R |
| 1. **Wie financiert in *(Nederland)* de meeste gezondheidszorg voor daklozen?**   *[Slechts een antwoord* **]** | 1 = overheid  2 = niet gouvermentele organisaties/ liefdadigheidsinstellingen  3 = Kerken en religieuze gemeenschappen  4 = DK  5 = R |
| 1. **Zou u zeggen dat in de omgeving waar u woont er veel, enkele, weinig of geen daklozen zijn?**   *[In de omgeving waar u woont = In uw buurt]* | 1 = Veel mensen  2 = Enkele mensen  3 = Weinig mensen  4 = Geen (*ga naar Q12)*  5 = DK  6 = R |
| 1. **Hoeveel verschillende daklozen ziet u gemiddeld per week?**   *[Als de deelnemer dezelfde persoon meerdere keren per week ziet, telt dit als 1 persoon.]* | 1 = geen  2 = 1 of 2  3 = 3 tot 10  4 = meer dan 10  5 = DK  6 = R |
| 1. **Zouden overheidsinstanties dakloosheid als prioriteit moeten beschouwen?** | 1 = Ja  2 = Nee  3 = DK  4 = R |
| 1. **Bent u op uw hoede als u een dakloze tegenkomt?** | 1= Vaak  2= Soms  3= Zelden  4= Nooit  5= DK  6 =R |
| 1. **Heeft u in het afgelopen jaar …**   *[Lees antwoordmogelijkheden voor]* | 1 = Ja  2 = Nee  3 = DK  4 = R |
| 1. Geld, eten of kleding aan een dakloze gegeven? | 1 2 3 4 |
| 1. Geld, eten of kleding gegeven aan een liefdadigheids- of non-profit organisatie voor daklozen? | 1 2 3 4 |
| 1. Vrijwilligerswerk gedaan in een liefdadigheids- of non-profit organisatie voor daklozen? | 1 2 3 4 |
| 1. Anders *[licht toe]*: ___________________ | 1 2 3 4 |
| 1. **Zou u, om dakloosheid te verminderen, bereid zijn om***[Lees antwoordmogelijkheden voor]***?** | 1 = YES  2 = NO  3 = DK  4 = R |
| 1. Meer belasting te betalen? | 1 2 3 4 |
| 1. Vrijwilligerswerk te doen? | 1 2 3 4 |
| 1. Een daklozenopvang in de buurt van uw huis te hebben? | 1 2 3 4 |

**Module *Bereidheid om te betalen***

*[Let op: lees* ***Nooit*** *DK of R optie voor]*

| [Interviewer Script]  Nu zouden we graag uw mening willen weten over verschillende interventies om dakloze mensen te huisvesten.  Weet u dat in (*NEDERLAND*) ongeveer 30.000 mensen dakloos zijn? Uit onderzoek in de 4 grote steden blijkt dat door dakloze mensen vaker alcohol en drugs worden gebruikt dan door de algemene bevolking.  In Nederland zijn verschillende vormen van opvang voor dakloze mensen, namelijk nachtopvang, crisisopvang, 24-uursopvang en wonen met begeleiding. Crisisopvang en 24-uursopvang is er voor mensen die om verschillende redenen niet meer thuis kunnen wonen. In deze opvang werken dakloze mensen via een begeleidingstraject aan het oplossen van hun problemen zodat ze weer zelfstandig kunnen wonen. Als mensen na de crisisopvang of 24-uurs opvang nog niet zelfstandig kunnen wonen bijvoorbeeld vanwege psychische problemen of verslaving, dan gaan ze begeleid wonen. Voor de opvang geldt een regiobinding dat betekent dat daklozen alleen kunnen aanmelden bij hun eigen centrumgemeente. Uitgangspunt van deze crisis en 24-uursopvang is dat het zo lang duurt als nodig is maar zo kort als mogelijk. Het kan verschillen tussen opvangvoorzieningen maar de duur van het verblijf in crisisopvang/24-uursopvang ligt tussen meestal een aantal weken tot een aantal maanden. In de opvangvoorzieningen zijn er vaak één of tweepersoons kamers maar met gedeelde keuken, badkamer en andere faciliteiten. Daarnaast zijn maatschappelijk werkers beschikbaar.  In het volgende deel leggen we u een innovatief huisvestingsprogramma voor daklozen voor.  Het doel is dat u ons vertelt hoeveel u bereid zou zijn om voor dit programma te betalen. We willen niet dat u de daadwerkelijke kosten van het programma inschat, maar dat u zegt welke waarde / welk nut u het programma geeft. Alles is hypothetisch en alleen voor onderzoeksdoeleinden. Er zal geen belastingverhoging plaatsvinden!  U kunt bereid zijn om veel, een beetje of helemaal niets te betalen.  Er zijn geen goede of slechte antwoorden. | | |
| --- | --- | --- |
| **Dit innovatieve programma heet *Housing First*.**  Het is gericht op dakloze mensen met geestelijke of lichamelijke gezondheidsproblemen (bijv., psychische aandoening, alcohol- of drugsverslaving, handicap).  Om aan dit programma deel te nemen, hoeft een dakloze geen behandeling te ondergaan voor psychische stoornissen of verslavingsproblemen.  Ze krijgen individuele huisvesting en medische en sociale zorg, aangepast aan hun behoeften (tot 7 dagen per week).  Ze betalen een deel van de huur, maar in geval van onvoldoende inkomen zijn er subsidies beschikbaar.  Een vergelijkbaar programma wordt momenteel getest in (*NEDERLAND*) en laat zien dat na de start van de Housing First praktijken de overgrote meerderheid (tussen 79% en 93%) van de daklozen stabiel en individueel gehuisvest is. | | |
| 1. **Ik ga bedragen in Euro’s voorstellen. Vertelt u me alstublieft hoeveel u per jaar door middel van belastingen voor dit programma zou willen betalen.**   *[Interviewer: als het spontane antwoord “nul Euro” of wil niet betalen, noteer 1 = JA hier.*  *Ga dan naar vraag 16.2]*  *[Interviewer: Na eerste weigering of “weet niet”, stop en ga door met de volgende vraag Q 16.1].*  **Zou u bereid zijn om………te betalen?**  *[Als de deelnemer het hoogste bod aanvaart(€*400*), ga dan naar vraag Q 16.1].* | 1 = JA  2 = Nee  3= DK  4 =R  0€ == > 1  *(Currency/change will be adapt to COUNTRY)*  €10? …… 1 2 3 4 *[83cts/maand]*  €25? …….1 2 3 4 *[2€/ maand]*  €50? …....1 2 3 4 *[4€// maand]*  €100? …...1 2 3 4 *[8€// maand]*  €200? …...1 2 3 4 *[17€// maand]*  €400? …...1 2 3 4 *[33€// maand]* |  |
| 1. **Wat zou het hoogste bedrag zijn dat u per jaar door middel van belastingen voor dit programma zou willen betalen?**   *[Herinner de deelnemer aan het laatste bedrag dat hij heeft geaccepteerd (en het bedrag dat hij heeft geweigerd, indien van toepassing) Het doel is om de deelnemer het maximum tussen deze twee vragen te laten bepalen. ].*  *Vb: Bij Ja voor 25 € en nee bij 50 €, vraag: Tussen 25 en 50 €, wat zou het maximale bedrag zijn….* | /-----------/ € |  |
| 1. *[Als respondent 0€ heeft geantwoord:]* **Zou u mij alstublieft de belangrijkste reden willen geven waarom u weigert? Is dat omdat**… | 1 = Het programma niet zal werken  2 = Andere programma’s belangrijker zijn / hogere prioriteit hebben  3 = Ik niet meer belasting wil betalen  *(ga naar Q18)*  4 = Ik niet meer belasting kan betalen *(ga naar Q18)*  5 = Anders: -------------------------------  6= DK  7 =R |  |
| 1. **Tot nu toe heb ik u verteld over een programma voor daklozen met psychische aandoeningen of verslaving. Zou u bereid zijn om per jaar door middel van belastingen (meer, hetzelfde of minder) te betalen als dit programma OPEN stond voor alle daklozen?** | 1= Meer *(ga naar Q17.1)*  2= Hetzelfde *(ga naar Q18)*  3= Minder *(ga naar Q17.1)*  4= DK  5 = R |  |
| 1. **Kunt u alstublieft het maximale bedrag noemen dat u bereid zou zijn per jaar door middel van belastingen te betalen voor dit programma?** | /----------/ € |  |
| 1. **Hoe zeker bent u van uw antwoord?** | 1= Helemaal zeker  2= redelijk zeker  3= min of meer zeker  4= redelijk onzeker  5 = Helemaal onzeker |  |
| 1. **Voor de data-analyse is het voor ons van belang om te weten of uw inkomen belastbaar is.** | 1 = Ja  2= Nee  3= DK  4 =R |  |

*[INTERVIEWER’S SCRIPT].* **Bedankt voor uw antwoorden. Nu wil ik u graag wat vragen stellen over de leefomstandigheden van daklozen in** (*Nederland).*

| 1. **Zou u zeggen dat het aantal daklozen in de afgelopen drie jaar … is?** | 1 = sterk gestegen  2 = enigszins gestegen  3 = enigszins gedaald  4 = sterk gedaald  5 = Hetzelfde gebleven (spontaan)  6 = DK   1. = R |
| --- | --- |
| 1. **Ik ga verschillende stellingen over daklozen voorlezen. Kunt u mij vertellen in hoeverre u het er mee eens bent..** *[Lees stelling voor]* | 1 = Helemaal mee eens  2 = enigszins mee eens  3 = enigszins mee oneens  4 = helemaal mee oneens  5= DK  6 =R |
| 1. Daklozen zijn slachtoffers van geweld (geweld, overvallen, bedreigingen en aanvallen). | 1 2 3 4 5 6 |
| 1. Zij worden gediscrimineerd bij het zoeken naar werk | 1 2 3 4 5 6 |
| 1. Zij eten minstens twee maaltijden per dag | 1 2 3 4 5 6 |
| 1. Zij zijn in staat om in contact te blijven met familie en vrienden. | 1 2 3 4 5 6 |
| 1. Zij hebben een kortere levensverwachting dan de algemene bevolking. | 1 2 3 4 5 6 |
| 1. Velen kiezen ervoor dakloos te blijven | 1 2 3 4 5 6 |
| 1. De meesten hebben vaardigheden om te kunnen werken. | 1 2 3 4 5 6 |
| 1. Ze zouden voor een huis kunnen zorgen (schoonhouden, inrichten) als ze dat hadden | 1 2 3 4 5 6 |
| 1. Ze hebben toegang tot betaald of onbetaald werk (vrijwilligerswerk, stage etc.). | 1 2 3 4 5 6 |
| 1. Hun voornaamste bron van inkomsten zijn sociale uitkeringen | 1 2 3 4 5 6 |
| 1. Zij brengen veel tijd alleen door, zonder een sociaal netwerk. | 1 2 3 4 5 6 |
| 1. **Voldoet de hulpverlening die wordt geboden ………… volgens u aan de behoeften van daklozen?** | 1 = Helemaal eens  2 = Enigszins eens  3 = Enigszins oneens  4 = Helemaal oneens  5= DK  6 =R |
| 1. In ziekenhuizen en Eerste Hulp posten | 1 2 3 4 5 6 |
| 1. Door huisartsen en poliklinisch specialisten | 1 2 3 4 5 6 |
| 1. In crisisopvang | 1 2 3 4 5 6 |
| 1. In tijdelijke opvang | 1 2 3 4 5 6 |
| 1. In het *Housing First* programma beter aan de behoeften van daklozen vergeleken met tijdelijke opvangvoorzieningen? | 1 2 3 4 5 6 |
|  |  |
| 1. **Wat zijn volgens u de DRIE redenen die het best verklaren waarom mensen dakloos worden?**   *[Let op: wacht op een spontaan antwoord en kruis het antwoord aan dat daar het dichtstbij ligt].* | 1 = baanverlies/ periode van werkloosheid  2 = onvoldoende inkomen / kan huur niet betalen  3 = huis vernietigd door ramp (brand, overstroming, enz)  4 = overmatige schuldenlast  5 = ziek of gehandicapt  6 = verslaving (alcohol, drugs of andere soort verslaving)  7 = beëindigen van relatie, scheiding of verlies van familielid  8 = psychische gezondheidsproblemen  9 = Geen toegang tot sociale uitkeringen of hulpverlening  10 = illegale immigratie  11 = Eigen Keuze  12 = anders 1:___________________________________  13 = anders 2:___________________________________  14 = anders 3:___________________________________  15 = Geen *(SPONTAAN)*  16= DK  17 =R |
| 1. **Wie zou volgens u voornamelijk verantwoordelijk moeten zijn voor het voorzien in NOODOPVANG voor daklozen ?** | 1 = overheid  2 = niet-gouvermentele organisaties/ liefdadigheidsinstellingen  3 = Kerken en religieuze gemeenschappen  4 = Daklozen zelf  5= DK  6 = R |
| 1. **Wie zou volgens u voornamelijk verantwoordelijk moeten zijn voor het voorzien in LANGDURIGE HUISVESTING voor daklozen?** | 1 = overheid  2 = niet-gouvermentele organisaties/ liefdadigheidsinstellingen  3 = Kerken en religieuze gemeenschappen  4 = Daklozen zelf  5= DK  6 = R |
| 1. **Welke groep daklozen zou prioriteit moeten krijgen in een programma voor langdurige huisvesting?**   *[Open vraag, de antwoordmogelijkheden moeten niet voorgelezen worden.*  *Wacht op het antwoord en kruis het antwoord aan dat het dichtsbij ligt. Of als deze er niet tussen staat, schrijf op onder ‘anders’ ]* | 1 = Families  2 = Vrouwen  3 = Jongeren [<30 jaar zonder kinderen]  4 = Mensen met psychische aandoeningen  5 = Werkenden  6 = ouderen  7 = mensen met beperkingen  8 = Werklozen  9 = Mensen met verslavingsproblemen (alcohol, drugs)  10 = Geen  11 = anders ___________________________________  12=DK  13=R |
| 1. **Vindt u dat de overheid over het algemeen (te veel, genoeg of te weinig) geld uitgeeft aan sociale voorzieningen?** | 1 = Te veel  2 = genoeg  3 = Te weinig  4 = DK  5 = R |
| 1. **Vindt u dat de overheid (te veel, genoeg, of te weinig) geld uitgeeft om daklozen te helpen?** | 1 = Te veel  2 = genoeg  3 = Te weinig *(go to Q30)*  4 = DK  5 = R *(go to Q30)* |
| 1. **Kunt u dit toelichten?**   *[Let op: Wacht op een spontaan antwoord en kruis dichtstbijzijnde antwoord aan]*  **Is dit omdat…** | 1 = Programma’s voor daklozen geen prioriteit zijn  2 = Het geldverspilling is omdat beleid inefficiënt is  3 = Het geldverspilling is omdat daklozen het niet verdienen  4 = De overheid geeft al voldoende uit om daklozen te helpen  5=Anders: ___________________________________  6=DK  7 =R |

***[INTERVIEWER’S SCRIPT]* Sociaal demografische gegevens.**

**Tot slot wil ik u een paar vragen stellen om uw sociaaldemografische profiel vast te stellen.**

| 1. *[ALLEEN voor interviewer, niet aan deelnemer vragen!]* | 1= Man  2=Vrouw |
| --- | --- |
| 1. **In welk jaar bent u geboren?**   *[noteer* ***4 cijfers****. Bijvoorbeeld : het jaar 1953 wordt genoteerd als 1953, 90 wordt gecodeerd als 1990.*  *[Als er AARZELING is, ga dan naar vraag over leeftijd]* | Jaar: /---/---/---/---/ |
| - 1. Hoe oud bent u? | leeftijd: /----------/ jaar |
| 1. **Wat is uw nationaliteit?** *[Schrijf land op]* | /_______________________/ |
| 1. **Wat is uw hoogst genoten opleiding?**   *Interviewer: Wacht op spontaan antwoord, geef de antwoordmogelijkheden niet.* | 1 = Postuniversitaire diploma (Master, PhD, etc/ 4 of meer jaar vervolgonderwijs)  2 = Bachelor of vergelijkbaar (tot 3 jaar vervolgonderwijs)  3 = Hoger onderwijs (tot 2 jaar vervolgonderwijs)  4 = [Eindexamen](http://context.reverso.net/traduction/neerlandais-francais/eindexamen) (VWO, VMBO, HAVO)  5 = Middelbare school afgerond  6 = Middelbare school  7 = Basisschool  8 = Niet naar school geweest  9 = Andere kwalificaties  10= DK  11= NA |
| 1. **Wat is uw huidige werksituatie?**   *[een antwoord mogelijk]* | 1 = voltijd werk (35 uur per week of meer)  2 = deeltijd werk (8 to 35 uur per week)  3 = af en toe deeltijd werk (minder dan 8 uur)  4 = Voltijd student  5 = gepensioneerd  6 = werkloos  7 = geen werk vanwege andere redenen  8 = DK  9 = NA |
| 1. **Wat is het bruto jaarinkomen van uw HUISHOUDEN (voor aftrek van inkomsten- of andere belastingen, zorgverzekering, enz.)?**   *[Interviewer: wacht op een spontaan antwoord en kruis het antwoord aan; bij weigering om het exacte bedrag te noemen moet de volgende schaal met bruto jaarinkomens voorgelegd worden:]*  *[Interviewer: Als de deelnemer om extra informatie vraagt:]* **U moet alle inkomstenbronnen meetellen, inclusief loon, pensioen, uitkeringen, rente van spaarrekeningen en lijfrentes.** | /----------------------------------------/€  1= ‘minder dan 5 000€’  2= tussen 5 000 & 10 000’€  3= tussen 10 000 & 15 000’€  4= tussen 15 000 & 20 000’€  5= tussen 20 000 & 30 000’€  6= tussen 30 000 & 40 000’€  7= tussen 40 000 & 50 000’€  8= tussen 50 000 & 60 000’€  9= tussen 60 000 & 70 000’€  10= meer dan 70 000 €  11 = DK  12 = R |
| 1. **Wat is uw burgerlijke staat? Bent u?** | 1 = getrouwd  2 = weduwe/weduwenaar  3 = uit elkaar/gescheiden  4 = in geregistreerd partnerschap/samenlevingscontract  5 = alleenstaand |
| 1. **Hoeveel mensen wonen er in totaal in uw huishouden, inclusief uzelf?** | /-------------------/ |
| 1. **Hoeveel kinderen heeft u?** | /------------------/ |
| 1. **Hoeveel afhankelijke kinderen zijn er (verbonden aan het belastbaar inkomen van het huishouden)?** | **/------------------/** |
| 1. **Wat is uw postcode?** *(4 cijfers)*   *[Als de deelnemer het niet weet of deze informatie niet wil geven vraag dan om de* ***dichtstbijzijnde*** *administratieve afdeling zoals* ***gemeente (NL****) ]* | /-----------------/ |
| 1. Gemeente | /------------------/ |
| 40.bis Volgens u, woont u **… ?** | 1 = in een stedelijke omgeving  2 = in de voorstadt (in de buiten wijken)  3 = in een plattelandsomgeving  4 = DK  5 = R |
| 1. **Hoeveel werkende vaste telefoonlijnen heeft u in uw huishouden?** (telefoonnummers die niet gebruikt worden niet meetellen- bijv. alleen voor internet of zakelijke telefoontjes) | /------------------/ |
| 1. **Hoeveel mobiele telefoons heeft uw huishouden?** (zakelijke telefoons niet meetellen) | /------------------/ |
| 1. Heeft u een mobiele telefoon? | 1 = Ja  2 = Nee  3 = R |

**EINDE VAN HET INTERVIEW**

**We zijn klaar met het interview!**

**Het Radboudumc bedankt u voor uw deelname.**

**Heeft u nog vragen?** *[Zo ja, de interviewer moet proberen zo goed mogelijk antwoord te geven].*

*[Als de interviewer niet kan antwoorden of het niet zeker weet]* ***Het spijt me. Ik heb deze informatie niet. Maar u kunt een email sturen naar*** [***Home_EU@ispa.pt***](mailto:Home_EU@ispa.pt)***.***

*[Instructie voor interviewer: Maak alstublieft een notitie voor de volgende training sessie.]*

*Vragen: ------------------------------------------------------------------------------------------------------------------------------------------------------------------------------------------------------------------------------------------------------------------*

**Als u later nog vragen of opmerkingen heeft, dan kunt u de projectleider, Dr.** *Tessa van Loenen*, **van**  *het Radboudumc* **bellen**.

**Het telefoonnummer is:** *024-3614420*.

**Wanneer dit onderzoek is afgerond, dan zal er een kort verslag van de resultaten beschikbaar komen op het volgende adres:** [**www.HOME.EU.ORG**](http://www.HOME.EU.ORG).

**Zoals gezegd, uw deelname is anoniem. Nogmaals bedankt. Een prettige dag verder.**

| 1. *VRAAG AAN INTERVIEWER: Heeft de respondent de meeste vragen op een redelijke manier beantwoord? Waren er problemen die de verkregen gegevens ongeldig of twijfelachtig kunnen maken?* | 1 = GEEN PROBLEMEN  2 = MOGELIJKE PROBLEMEN (beschrijf hieronder): _________________________________________________  _________________________________________________  3 = ERNSTIGE PROBLEMEN (beschrijf hieronder): _________________________________________________  _________________________________________________ |  |
| --- | --- | --- |

*OPMERKING VOOR INTERVIEWER: NEEM DE VRAGENLIJST ALSJEBLIEFT DOOR OM ZEKER TE WETEN DAT VRAGEN VOLLEDIG EN JUIST ZIJN INGEVULD. BEL DEELNEMER ZO NODIG TERUG OM PROBLEMEN OP TE HELDEREN.*

| Encuesta telefónica de opinión y de las preferencias de LA CIUDADANÍA sobre las personas sin hogar. |
| --- |
| NÚMERO DE IDENTIFICACIÓN: /__/__/__/__/__/  FECHA: (____/____/____)  HORA DE INICIO DE LA ENTREVISTA: (____:____) HORA FINAL: (____:____)  ENTREVISTADOR/A: _________________________ CÓDIGO ENTREVISTADOR/A: __________ |

1. ***INTROCUCCIÓN***

*[GUIÓN DEL ENTREVISTADOR/A]*

**Hola, mi nombre es ----------------------------------. Llamo de la RAIS Fundación. Su domicilio ha sido seleccionado aleatoriamente para participar en una encuesta europea sobre la opinión de la población española acerca de las personas sin hogar.**

**¿Es Usted mayor de edad?**

*[Si Sí, ve a la parte II disponibilidad para la encuestA]*

*[Si No, pregunta]:* **¿Podría hablar con alguna persona mayor de edad que esté en el domicilio?**

*[Si Sí, cuando la persona mayor de edad responda, continua con lo siguiente]:* **Hola, mi nombre es ----------------------------------. Llamo de la RAIS Fundación, en Francia. Su domicilio ha sido seleccionado aleatoriamente para participar en una encuesta europea sobre la opinión de la población española acerca de las personas sin hogar.**

*[y pasa a la parte II disponibilidad para la encuestA]*

1. *[Si no hay una persona adulta en el domicilio pregunta:]* **¿Cuándo podría localizar a alguien mayor de edad en casa?**

[REGISTRO PARA VOLVER A LLAMAR]: Fecha: _______ Hora: ________NOMBRE (sólo nombre, no apellidos) ________ **Gracias. Volveremos a llamar entonces.**

1. [Si no sabe cuándo:]: **Gracias. Volveremos a llamar en otro momento.**
2. ***DISPONIBILIDAD PARA LA ENCUESTA***

**¿Puede dedicarme unos 20 minutos?**

1. [*Si Sí]:* **Gracias!** *(y luego pasa a la sección V PREPARANDO DE LA ENTREVISTA)*
2. *[Si No, pregunta*:] ¿**Cuál sería un momento adecuado para llamarle?**

[REGISTRO PARA VOLVER A LLAMAR]: Fecha: _______ Hora: ________ **Gracias. Volveremos a llamar entonces.**

*[Si es que No o No sabe, di:*] **Nos gustaría realmente conocer su opinión sobre este tema. ¿De verdad no podemos hablar ahora o en otro momento?**

- [*Si responde Sí]:* **Gracias!** *(y luego pasa a la sección V PREPARANDO DE LA ENTREVISTA)*
- [*Si responde Sí, pero en otro momento, anota Fecha y hora:]*

Fecha: _______ Hora: ________ **Gracias. Volveremos a llamar entonces.**

- *[Si la respuesta es No, ve a la sección III negativa].*

1. ***NEGATIVA***
   1. **Entendemos que no quiera participar, pero ¿podría ayudarnos a analizar las respuestas negativas diciéndonos su edad, último nivel de educación y su situación laboral?**

1 = Sí

2 = No

- 1. **Año de nacimiento: ____________________**
  2. **Último nivel de educación**

1 = Master, Doctorado, etc… (5 años o más de educación universitaria)

2 = Licenciatura (4 años de educación universitaria)

3 = Diplomatura

4 = Título de Bachillerato

5 = Estudios de Bachillerato sin título

6 = Educación Secundaria Obligatoria

7 = Educación Primaria/ Educación General Básica

8 = No ha asistido a la escuela

9 = Otra cualificación

10= No sabe

11= No contesta

- 1. **Ocupación:**

1 = Trabajo a tiempo completo (35 horas o más a la semana)

2 = Trabajo a tiempo parcial (de 8 a 35 horas a la semana)

3 = Trabajo a tiempo parcial ocasional (menos de 8 horas a la semana)

4 = Estudiante a tiempo completo

5 = Jubilado/a

6 = Desempleado/a

7 = No trabaja por otros motivos

8 = No sabe

9 = No contesta

- 1. **¿Cuántas personas viven en su domicilio, incluyéndose Usted? ____**
  2. **¿Cuántas personas menores de edad viven en su domicilio? ______**

**Gracias por su ayuda y disculpe la molestia.**

1. ***LA PERSONA ENCUESTADA NO HABLA ESPAÑOL CORRECTAMENTE***

*[Nota: Si habla con alguien que habla un idioma extranjero y cuyo español es claramente insuficiente para responder la entrevista, diga:]*

***Perdón, solo hablo español. Gracias por su tiempo. Buenos días/Buenas tardes***

1. ***PREPARANDO LA ENTREVISTA***

*[GUIÓN PARA LA PERSONA ENTREVISTADORA]*

**Esta encuesta telefónica forma parte de una gran encuesta que se está llevando a cabo en 8 países europeos. El objetivo de la encuesta es analizar la opinión de la ciudadanía sobre las personas sin hogar y entender sus expectativas al respecto. Tenga en cuenta que durante esta entrevista solo le preguntaré sobre la situación de las personas sin hogar en España. Sus respuestas serán estrictamente anónimas. No le preguntaré por su nombre o apellidos. Si hay alguna pregunta que no quiere responder, simplemente dígamelo.**

**¿Está de acuerdo en participar en esta encuesta para medir la opinión de la ciudadanía sobre las personas sin hogar?** Sí

No *(ve a la sección III negativa)*

*[Si sí]* **Muy bien, pues comencemos!**

*[GUIÓN PARA LA PERSONA ENTREVISTADORA]* **Voy a empezar preguntándole sobre cuestiones generales acerca de las personas sin hogar. Antes de eso, debería saber que nosotros definimos como una persona sin hogar a quien duerme en la calle o en un coche, o vive en un albergue temporal o un alojamiento de emergencia.**

| *[Nota:* ***Nunca*** *leas las opciones NS/NC]* | NS= No sabe  NC= No contesta |
| --- | --- |
|  |  |
| 1. **¿Ha estado alguna vez sin hogar?**   *[ nota: incluso si es por una noche, cuenta como SÍ]* | 1 = SÍ  2 = NO *(pasa a la pregunta Q4)*  3 = NC *(pasa a la pregunta Q4)* |
| 1. *[Si responde Sí a P1]:* **¿Cuándo fue?** *[lee las opciones]* | 1 = En los últimos 12 meses  2 = Hace 1 o 2 años  3 = Hace 3 o 4 años  4 = Hace 4 o 5 años  5 = Hace más de 5 años  6 = NS  7 = NC |
| 1. *[Si responde “Sí” a P1]:* **¿Cuánto tiempo en total ha estado sin hogar a lo largo de su vida?** *[lee las opciones]* | 1 = Menos de una semana  2 = Menos de un mes  3 = Menos de un año  4 = Menos de dos años  5 = Menos de cuatro años  6 = Más de cuatro años  7 = NS  8 = NC |
| 1. **¿Algún miembro de su familia, amistades o personas conocidas han estado alguna vez en situación de sin hogar?** | 1 = SÍ  2 = NO *(pasa a P6)*  3 = NS *(pasa a P6)*  4 = NC *(pasa a P6)* |
| 1. *[Si algún familiar o amistades fueron personas sin hogar = Sí]* **Quién fue …** | 1 = Padre o madre  2 = Hijo/a  3 = Hermano/a  4 = Marido/Esposa/Pareja  5 = Amigo/a  6 = Otro familiar  7 = Conocido/a  8 = NS  9 = NC |
| 1. **¿Podría decirme aproximadamente cuántas personas sin hogar hay en España?**   *[la persona debe dar un número entero, no un porcentaje]* | /_______________/ |
| 1. **En su opinión, dentro de las personas sin hogar, cuál es el porcentaje de personas…** *[la persona debe dar un porcentaje]* |  |
| 7.1. ¿con problemas de salud mental? | _______% |
| - 1. ¿con problemas de adicciones (alcohol o drogas)? | _______% |
| 1. **En España, ¿quién financia la mayor parte de los servicios sociales para personas sin hogar?**   *[Solo una respuesta]* | 1 = Estado o administración pública  2 = ONGs, asociaciones o fundaciones  3 = Iglesia o comunidades religiosas  4 = NS  5 = NC |
| 1. **En España, ¿quién financia la mayor parte de los servicios de atención sanitaria para personas sin hogar?**   *[Solo una respuesta]* | 1 = Estado o administración pública  2 = ONGs o entidades de caridad  3 = Iglesia o comunidades religiosas  4 = NS  5 = NC |
| 1. **En la zona donde vive, por favor, dígame si hay personas sin hogar. ¿Diría que hay muchas personas, algunas personas, pocas personas o ninguna persona sin hogar?** | 1 = Muchas personas  2 = Algunas personas  3 = Pocas personas  4 = No hay (*Pasa a P 12)*  5 = NS  6 = NC |
| 1. **Aproximadamente, ¿cuántas personas sin hogar diferentes se encuentra Usted en una semana?**   *[Si la persona responde que ve a la misma persona varias veces en una semana, cuenta como 1 persona]* | 1 = Ninguna  2 = 1 o 2  3 = De 3 a 10  4 = Más de 10  5 = NS  6 = NC |
| 1. **¿Deberían las autoridades públicas considerar a las personas sin hogar como una prioridad?** | 1 = SÍ  2 = NO  3 = NS  4 = NC |
| 1. **Cuando pasa cerca de una persona sin hogar, ¿actúa con precaución?** | 1= A menudo  2= A veces  3= Casi nunca  4= Nunca  5= NS  6 =NC |
| 1. **Durante el año pasado, ha …**   *[leer las opciones 13.1 a 13.4]* | 1 = SÍ  2 = NO  3 = NS  4 = NC |
| 1. …dado dinero, comida o ropa a alguna persona sin hogar? | 1 2 3 4 |
| 1. …dado dinero, comida o ropa a alguna asociación, fundación u ONG para personas sin hogar? | 1 2 3 4 |
| 1. …sido voluntario/a en una asociación, una fundación u ONG para personas sin hogar? | 1 2 3 4 |
| 1. 13.4. Otros *[especificar]*: ___________________ | 1 2 3 4 |
| 1. **Para reducir el número de las personas sin hogar, ¿estaría dispuesto/a a…** *[leer opciones]* | 1= SÍ  2= NO  3= NS  4= NC |
| 1. …pagar más impuestos? | 1 2 3 4 |
| 1. …hacer voluntariado? | 1 2 3 4 |
| 1. …tener un albergue para personas sin hogar cerca de su casa? | 1 2 3 4 |

**Modulo *Disposición a pagar***

*[Nota:* ***Nunca*** *leas las opciones NS o NC]*

| [Guion de la persona entrevistadora]  Querríamos ahora conocer su opinión sobre diferentes tipos de intervención para alojar a personas sin hogar.  ¿Sabe usted que en ESPAÑA hay alrededor de 40.000 personas sin hogar, de las cuales al menos un 30% tiene problemas de adicciones al alcohol o a las drogas y/o que sufre problemas de enfermedad mental severa, aunque no se conocen las cifras reales?  Las soluciones que se ofrecen a estas personas actualmente son o albergues de emergencia o alojamientos temporales.  En los albergues de emergencia, las personas sin hogar tienen un tiempo limitado de estancia de una semana máximo, tras el cual vuelven a la calle. En los alojamientos temporales, las personas pueden acceder a un alojamiento de transición después de varios meses en lista de espera, donde a menudo duermen en dormitorios de varias personas. Allí pueden pasar entre 3 y 6 meses, siempre que cumplan con las normas del alojamiento, como los horarios de entrada y salida, y que no tengan problemas de salud mental o de adicciones. Por lo general, no se admiten parejas ni personas con animales de compañía. En estos servicios hay trabajadores sociales, y habitualmente se pide a la persona una contribución económica en base a sus ingresos.  En la siguiente sección, le vamos a presentar un programa de vivienda innovadora para personas sin hogar.  El objetivo es que nos diga cuánto estaría dispuesto/a a pagar por este programa. En ningún momento le pediremos una contribución económica. Solo queremos saber la utilidad del programa. Todo esto es una hipótesis y sólo con propósitos de investigación. ¡No se preocupe que no se van aumentar los impuestos!  Puede que esté dispuesto/a a pagar mucho, poco o nada. No hay una respuesta correcta o incorrecta. | | |
| --- | --- | --- |
| **Este programa innovador se llama Hábitat**  Está dirigido a personas sin hogar con problemas de salud mental o física, como enfermedades mentales, adicción al alcohol o a las drogas o con discapacidad.  Para acceder al programa, no se exige a la persona sin hogar que siga un tratamiento para problemas de salud mental o adicciones.  Se les ofrece una casa individual y recibe asistencia médica y social adaptada a sus necesidades (hasta 7 días por semana).  Las personas pagan parte de su alquiler, pero en caso de ingresos insuficientes, se les facilitan ayudas para cubrir el coste total.  En España, se está probando este programa desde 2014 y ha demostrado que, dos años después de entrar en el programa, más del 95% de las personas sin hogar alojadas siguen en sus viviendas. | | |
| 1. **Le voy a proponer ahora varias cantidades en euros. Por favor, dígame qué cantidad estaría dispuesto/a a pagar a través de impuestos cada año para este programa.**   *[Persona entrevistadora: si la respuesta es espontáneamente “Cero euros” o no quiere pagar nada, marca 1 = SÍ aquí.*  *Luego pasa a la P. 16.2]*  *[Persona entrevistadora: tras el primer RECHAZO o “NO SÉ”, para y continúa con la siguiente pregunta P. 16.1]*  **Estaría dispuesto/a a pagar ………………………**  *[Si la persona entrevistada acepta la cifra más alta (€*400*), pasa a la P. 16.1].* | 1 = SÍ  2 = NO  3= NS  4 =NC  0€ == > 1  €10? …… 1 2 3 4 *[83cts/mes]*  €25? ……. 1 2 3 4 *[2€/ mes]*  €50? …..... 1 2 3 4 *[4€// mes]*  €100? …... 1 2 3 4 *[8€// mes]*  €200? …... 1 2 3 4 *[17€// mes]*  €400? …... 1 2 3 4 *[33€// mes]* |  |
| 1. **¿Cuánto sería lo máximo que estaría dispuesto/a a pagar a través de impuestos cada año por este programa?**   *Recuerda a la persona la última cantidad que ha aceptado (y la cantidad que ha rechazado en su caso). El objetivo es que la persona indique una cantidad máxima entre esos dos valores].*  *Ej: si Sí a 25€ y no a 50€, pregunta: Entre 25 y 50€, cuántos impuestos estaría...* | /-----------/ € |  |
| 1. *[Si la persona ha respondido 0€]* **Por favor, ¿podría decirme el motivo por el que no pagaría nada? Es porque…** | 1 = El programa no funcionaria  2 = Otros programas son más importantes o prioritarios.  3 = No **quiero** pagar más impuestos  *(ve a P. 18)*  4 = No **puedo** pagar más impuestos (*ve a P. 18)*  5 = Otro motivo:_____________  6= NS  7 =NC |  |
| 1. **Hasta ahora le estaba preguntando por un programa para personas sin hogar con problemas de salud mental o de adicciones. ¿Estaría dispuesto/a a pagar (más, lo mismo o menos) cada año a través de impuestos si este programa fuese ACCESIBLE A TODAS las personas sin hogar?** | 1= Más *(ir a P 17.1)*  2= Lo mismo *(ir a P 18)*  3= Menos *(ir a P 17.1)*  4= NS  5 = NC |  |
| 1. **Especifique la cantidad máxima que estaría dispuesto/a a pagar a través de impuestos cada año por este programa** | /----------/ € |  |
| 1. **¿Está usted seguro/a de querer pagar esa suma de dinero?** | 1=Totalmente seguro  2=Casi seguro  3=No tan seguro  4=Para nada seguro  5 = NS  6= NC |  |
| 1. **Para poder hacer el análisis de los datos, necesitamos saber si sus ingresos están sujetos a impuestos.** | 1 = SÍ  2= NO  3= NS  4 =NC |  |

*[GUÍON DE LA PERSONA ENTREVISTADORA].* **Gracias por sus respuestas. Ahora querría hacerle algunas preguntas sobre las condiciones de vida de las personas sin hogar en España.**

| 1. **En los últimos 3 años, diría que el número de personas sin hogar en España ha…** | 1 = Aumentado mucho  2 = Aumentado un poco  3 = Disminuido un poco  4 = Disminuido mucho  5 = Es el mismo (espontáneo)  6 = NS  7 = NC |
| --- | --- |
| 1. **Leeré ahora varias afirmaciones sobre las personas sin hogar. Por favor dígame si está…** *[Lee los ítems]* | 1 = Completamente de acuerdo  2 = Algo de acuerdo  3 = Algo en desacuerdo  4 = Completamente en desacuerdo  5= NS  6 =NC |
| 1. Las personas sin hogar son víctimas de agresiones (violencia, robos, amenazas y ataques) | 1 2 3 4 5 6 |
| 1. Son discriminadas a la hora de ser contratadas | 1 2 3 4 5 6 |
| 1. Comen al menos dos veces al día | 1 2 3 4 5 6 |
| 1. Mantienen el contacto con la familia y amistades | 1 2 3 4 5 6 |
| 1. Tienen una esperanza de vida menor que la de la población general | 1 2 3 4 5 6 |
| 1. Muchas siguen sin hogar porque quieren | 1 2 3 4 5 6 |
| 1. La mayoría tienen habilidades para trabajar | 1 2 3 4 5 6 |
| 1. Podrían ocuparse de una casa (decorarla, tenerla limpia…) si tuvieran una | 1 2 3 4 5 6 |
| 1. Tienen acceso a empleo remunerado o no remunerado (voluntariado, prácticas…) | 1 2 3 4 5 6 |
| 1. Su principal fuente de ingresos son las ayudas sociales | 1 2 3 4 5 6 |
| 1. Pasan mucho tiempo solas, fuera de cualquiera red social | 1 2 3 4 5 6 |
| 1. **En su opinión, los servicios proporcionados a las personas sin hogar ……***[Lee los ítems]* **se ajustan a sus necesidades:** | 1 = Completamente de acuerdo  2 = Algo de acuerdo  3 = Algo en desacuerdo  4 = Completamente en desacuerdo  5= NS  6 =NC |
| 1. En hospitales y servicios de Urgencias | 1 2 3 4 5 6 |
| 1. Por médicos de familia y especialistas de consulta externa | 1 2 3 4 5 6 |
| 1. En centros de emergencia   *[Si la persona pregunta: para estancias máximas de 7 días]* | 1 2 3 4 5 6 |
| 1. En albergues de transición   *[Si la persona pregunta: para estancias máximas de 3 a 6 meses]* | 1 2 3 4 5 6 |
| 1. Un programa *Housing First* se ajusta mejor a las necesidades de las personas sin hogar comparado con un albergue de transición | 1 2 3 4 5 6 |
| 1. **En su opinión, ¿cuáles son las TRES razones por las que una persona se queda sin hogar?**   *[Nota: espera a que responda espontáneamente y marca la respuesta más cercana].* | 1 = pérdida del empleo o periodo de desempleo  2 = ingresos insuficientes o no puede pagar la vivienda  3 = vivienda destruida por una catástrofe (fuego, inundaciones, etc.)  4 = sobreendeudamiento  5 = enfermedad o discapacidad  6 = adicciones (alcohol, drogas u otros tipos de adicción)  7 = ruptura de pareja, divorcio o pérdida de un miembro de la familia  8 = problemas de salud mental  9 = no accede a prestaciones sociales o servicios de apoyo  10 = inmigración ilegal  11 = decisión propia  12 = Otro 1:___________________________________  13 = Otro 2:___________________________________  14 = Otro 3:___________________________________  15 = Ninguno *(espontáneo)*  16= NS  17 =NC |
| 1. **En su opinión, ¿de quién debería ser principalmente la responsabilidad de facilitar ACOGIDA DE EMERGENCIA a las personas sin hogar?** | 1 = Estado o administración pública  2 = ONGs, asociaciones o fundaciones  3 = Iglesia o comunidades religiosas  4 = Las propias personas sin hogar  5 = NS  6 = NC |
| 1. **En su opinión, ¿de quién debería ser principalmente la responsabilidad de facilitar VIVIENDA PERMANENTE a las personas sin hogar?** | 1 = Estado o administración pública  2 = ONGs, asociaciones o fundaciones  3 = Iglesia o comunidades religiosas  4 = Las propias personas sin hogar  5 = NS  6 = NC |
| 1. **Dentro de las personas sin hogar ¿qué grupo debería tener prioridad para acceder a un programa de vivienda permanente?**   *[Es una pregunta abierta, las respuestas no deberían leerse. Espera que responda y marca la respuesta más cercana o si no anótala en “Otro”]* | 1 = Familias  2 = Mujeres  3 = Jóvenes [menores de 30 sin hijos/as a cargo]  4 = Personas con problemas de salud mental  5 = Personas con trabajo  6 = Personas mayores  7 = Personas con discapacidad  8 = Personas desempleadas  9 = Personas con problemas de adicción al alcohol o drogas  10 = Ninguno  11 = Otro: ___________________________________  12=NS  13=NC |
| 1. **En general, ¿cree que el Estado invierte (demasiado, suficiente o muy poco) dinero en el sistema de bienestar social?** | 1 = Demasiado  2 = Suficiente  3 = Muy poco  4 = NS  5 = NC |
| 1. **En concreto, ¿cree que el Estado invierte (demasiado, suficiente o muy poco) dinero para ayudar a las personas sin hogar?** | 1 = Demasiado  2 = Suficiente  3 = Muy poco (ve a la P 30)  4 = NS  5 = NC (ve a la P 30) |
| 1. **¿Podría especificar por qué?**   *[Nota: espera a una respuesta espontánea y marca la respuesta más parecida]* | 1 = Los programas para las personas sin hogar no son prioritarios  2 = Es un gasto de dinero porque las políticas son ineficientes  3 = Es un gasto de dinero porque las personas sin hogar no merecen la ayuda  4 = El gobierno invierte de manera apropiada para ayudar a las personas sin hogar.  5= Otro: ___________________________________  6=NS  7 =NC |

***[Guión de la persona entrevistadora]* CARACTERÍSTICAS sociodemográficas**

**Finalmente, le preguntaré algunas cuestiones para definir su perfil sociodemográfico.**

| 1. *[Para la persona ENTREVISTADORA SOLO, ¡no preguntes a la persona que responde!]* | 1= hombre  2= mujer |
| --- | --- |
| 1. **¿En qué año nació?**   *[Codifica los* ***4 dígitos****, es decir, 1953 se codifica 1953, y “en el 90” se codifica como 1990]*  *[Si hay DUDAS, para a la siguiente pregunta]* | Año: /---/---/---/---/ |
| - 1. ¿Qué edad tiene? | Edad: /----------/ años |
| 1. **¿Cuál es su nacionalidad?** *[escribe el país]* | /_______________________/ |
| 1. **¿Cuál es su nivel de estudios?**   *Persona entrevistadora: Esperamos una respuesta espontánea, no des las opciones de respuesta…* | 1 = Master, Doctorado, etc… (5 años o más de educación universitaria)  2 = Licenciatura (4 años de educación universitaria)  3 = Diplomatura / Ciclo Formativo de Grado Superior (CFGS) / Formación Profesional de Segundo Grado (FPII)  4 = Título de Bachillerato  5 = Estudios de Bachillerato sin título  6 = Educación Secundaria Obligatoria  7 = Educación Primaria/ Educación General Básica  8 = No ha asistido a la escuela  9 = Otra cualificación  10= No sabe  11= No contesta |
| 1. **Actualmente, ¿cuál es su situación laboral?**   *[Se espera sólo una respuesta]* | 1 = Trabajo a tiempo completo (35 horas o más a la semana)  2 = Trabajo a tiempo parcial (de 8 a 35 horas a la semana)  3 = Trabajo a tiempo parcial ocasional (menos de 8 horas a la semana)  4 = Estudiante a tiempo completo  5 = Jubilado/a  6 = Desempleado/a  7 = No trabaja por otros motivos  8 = NS  9 = NC |
| 1. **¿Cuál es el ingreso anual bruto de su HOGAR antes de pagar impuestos y deducciones (sobre la renta u otros impuestos, seguros privados de salud, otras tasas, etc.)?**   *[Persona entrevistadora: espera una respuesta espontánea y marca la respuesta adecuada; si no quiere dar la cifra exacta, propón las opciones de la siguiente escala de ingresos anuales brutos:]*  *[Si la persona pide más información, puedes precisar:]* **Debería incluir todas las fuentes de ingresos, como pagos por actividades, pensiones, prestaciones sociales, intereses de ahorros o rentas vitalicias.** | /----------------------------------------/€  1= menos de 5 000€  2= entre 5 000 & 10 000’€  3= entre 10 000 & 15 000’€  4= entre 15 000 & 20 000’€  5= entre 20 000 & 30 000’€  6= entre 30 000 & 40 000’€  7= entre 40 000 & 50 000’€  8= entre 50 000 & 60 000’€  9= entre 60 000 & 70 000’€  10= más de 70 000 €  11 = NS  12 = NC |
| 1. **¿Cuál es su estado civil? Está…?** | 1 = casado/a  2 = viudo/a  3 = divorciado/a o separado/a  4 = pareja de hecho  5 = soltero/a |
| 1. **Incluyéndose a Usted mismo/a, ¿cuántas personas viven en su hogar?** | /-------------------/ |
| 1. **¿Cuántos hijos o hijas tiene Usted?** | /------------------/ |
| 1. **¿Cuántos menores dependientes hay en el hogar (es decir, dependientes de los ingresos sujetos a impuestos)?** | **/------------------/** |
| 1. **¿Cuál es su Código Postal?**   *[Si la persona no lo sabe o no quiere dar esta información, pregunta por la Provincia]* | /-----------------/ |
| 1. Provincia |  |
| 1. ¿Diría que vive en…? | 1= una zona urbanas  2= un área suburbana  3= una zona rural  4= NS  5= NC |
| 1. **¿Cuántas líneas de teléfono fijo operativas hay en su vivienda** (sin contar líneas que sólo sirvan para internet o teléfonos de empresa)**?** | /------------------/ |
| 1. **¿Cuántas líneas de teléfono móvil hay en su vivienda** (sin contar teléfonos de empresa)**?** | /------------------/ |
| 1. ¿Tiene Usted teléfono móvil? | 1 = Sí  2 = No  3 = NC |

**FIN DE LA ENTREVISTA**

**Bien, hemos acabado la entrevista.**

**La RAIS Fundación agradece su participación.**

**¿Tiene alguna pregunta?** *[Si Sí, la persona entrevistadora debería intentar responder].*

*[Si la persona entrevistadora no puede responder o no lo tiene claro]* ***Disculpe, no tengo esa información. Pero puede enviar un email a la coordinación del proyecto:*** [***HOME_EU@ispa.pt***](mailto:HOME_EU@ispa.pt)

*[Instrucciones a la persona entrevistadora: POR FAVOR, anota la pregunta para la siguiente sesión de formación.]*

*Pregunta: ------------------------------------------------------------------------------------------------------------------------------------------------------------------------------------------------------------------------------------------------------------------*

**También, si tiene alguna pregunta o comentario posterior, puede contactar con la entidad responsable del proyecto en España, RAIS Fundación, en el correo electrónico:** [home.eu.communic@raisfundacion.org](http://home.eu.communic@raisfundacion.org)

**Habrá también un breve informe de resultados de esta encuesta una vez que se haya completado en la dirección:** [**www.HOME-EU.ORG**](http://www.HOME-EU.ORG).

**Recuerde, su participación es anónima. Gracias de nuevo. Que tenga buen día.**

| 1. *PREGUNTA A PERSONA ENSTREVISTADORA: ¿Respondió la persona entrevistada de manera razonable la mayor parte de preguntas? ¿Hubo algún problema que crees que puede invalidar o cuestionar los datos obtenidos?* | 1 = SIN PROBLEMAS  2 = POTENCIALES PROBLEMAS (describe abajo): _________________________________________________  _________________________________________________  3 = PROBLEMAS SERIOS (describe abajo): _________________________________________________  _________________________________________________ |  |
| --- | --- | --- |

*NOTA PARA LA PERSONA ENTREVISTADORA: POR FAVOR, VUELVE AL INICIO Y REVISA QUE TODOS LOS ITEMS ESTÁN COMPLETOS Y PUNTUADOS CORRECTAMENTE. SI ES NECESARIO, VUELVE A LLAMAR A LA PERSONA PARA ACLARAR POSIBLES PROBLEMAS.*

| SONDAGGIO CITTADINO  SULL’OPINIONE PUBBLICA RIGUARDO  AL FENOMENO DELLE PERSONE SENZA DIMORA E PREFERENZE |
| --- |
| NUMERO IDENTIFICATIVO: /__/__/__/__/__/  DATA: (____/____/____)  ORA D’INIZIO DELL’INTERVISTA : (____:____) ORA DI FINE: (____:____)  INTERVISTATORE : _________________________ CODICE INTERVISTATORE : __________ |

1. ***INTRODUZIONE***

*[TESTO PER L’INTERVISTATORE]*

**Salve, il mio nome è ----------------------------------. Sto chiamando per conto dell’università di Padova**

**. La sua famiglia è stata scelta in maniera casuale per prendere parte a un sondaggio Europeo sull’ OPINIONE RIGUARDO AL FENOMENO DELLE PERSONE SENZA DIMORA in Italia.**

**Lei è maggiorenne?**

*[Se si, allora andare alla parte II DISPONIBILITA’ AL SONDAGGIO]*

*[Se no, allora chiedere]:* **Potrei parlare con qualcuno di maggiorenne della sua famiglia ora?**

1. *[Se si, quando la persona arriva al telefono, continuare con il seguente]:* **Salve, il mio nome è ----------------------------------. Sto chiamando per conto dell’ Università di Padova. La sua famiglia è stata scelta in maniera casuale per prendere parte a un sondaggio Europeo sull’ OPINIONE RIGUARDO AL FENOMENO DELLE PERSONE SENZA DIMORA in Italia.**
2. *[Se no, la persona adulta non è a casa, allora chiedere*:] **Qual è il momento migliore per richiamare questa persona ?**
3. [SEGNARE PER RICHIAMARE]: Data: _______ Ora: ________NOME (solo il primo) _______ **Grazie. Allora richiameremo.**
4. [Se non sa o no:]: **Grazie. Riproveremo ancora in un altro momento**;
5. ***DISPONIBILITA’ AL SONDAGGIO***

**Possiamo parlare per circa 20 minuti?**

1. [*Se si]:* **Grazie!** *(e andare alla sezione V* *REALIZZAZIONE DELL’INTERVISTA)*
2. *[Se no, chiedere*:] **Quale potrebbe essere un momento migliore per richiamarla?**

[SEGNARE PER RICHIAMARE]: Data: _______ Ora: ________NOME (solo il primo) _______ **Grazie. Allora richiameremo più tardi.**

- - *[Se no o non sa, dire*:] **Ci piacerebbe davvero avere la sua opinione su questo argomento. È sicuro che non possiamo parlare ora o in un altro momento?**
    - *[Se la risposta è si].* **Grazie!** *(e andare alla sezione V* *REALIZZAZIONE DELL’INTERVISTA]*
    - [*Se la risposta è Si ma in un altro momento, segnare Data/Ora:]*

Data:_______________ Ora: _______________ **Grazie. Richiameremo più tardi.**

- - - *[Se no, andare alla sezione III RIFIUTO].*

1. ***RIFIUTO***
   1. **Abbiamo capito che non vuole partecipare; ma potrebbe aiutarci ad analizzare i rifiuti dicendoci la sua età, il suo livello di istruzione più alto e il suo stato occupazionale?**

1 = Si

2 = No

- 1. **Anno di nascita: ____________________**
  2. **Livello di istruzione più alto :**

1 = Laurea Magistrale o Master o Dottorati (formazione universitaria di quattro anni o più).

2 = Laurea triennale o equivalente (fino a 3 anni di scuola dopo le superiori)

3 = Istruzione superiore (fino a 2 anni di formazione dopo le superiori)

4 = Diploma di scuola superiore

5 = Studi superiore interroti

6 = Scuola media

7 = Scuola elementare

8 = Non ho frequentato la scuola

9 = Altra qualificazione

10= NON SO

11= Rifiuto a rispondere

- 1. **Stato Occupazionale:**

1 = Lavoro Full time (35 ore o più alla settimana)

2 = Lavoro part time (da 8 a 35 ore alla settimana)

3 = Lavoro part time occasionalmente (meno di 8 ore)

4 = Studente a tempo pieno

5 = Pensionato

6 = Disoccupato

7 = Non sto lavorando per altre ragioni

8 = NON SO

9 = Rifiuto a rispondere

- 1. **Quante persone vivono nella sua famiglia, incluso lei? ____**
  2. **Quanti bambini (o minorenni )vivono nella sua famiglia? ______**

**Grazie per il suo aiuto e** **Ci scusi per il disturbo.**

1. ***L’INTERVISTATO NON PARLA*** *italiano* ***ADEGUATAMENTE***

*[Nota: Se trovate qualcuno che parla una lingua straniera (e il cui Italiano è evidentemente insufficiente per completare l’intervista), dire:]*

***Mi scusi; Io parlo solo*** *italiano.* ***Grazie per il suo tempo, Arrivederci.***

1. ***REALIZZAZIONE DELL’INTERVISTA***

*[TESTO PER L’INTERVISTATORE]*

**Questo sondaggio telefonico è parte di un sondaggio più ampio condotto in 8 Stati Europei. L’obiettivo è misurare le opinioni dei cittadini riguardo al tema delle persone senza dimora e comprendere le loro aspettative sul tema. La prego di notare che durante questa intervista sarà affrontata solo la sua opinione per quanto riguarda la situazione in** *ITALIA***. Le sue risposte rimarranno rigorosamente anonime. Non le chiederemo il suo nome o il suo cognome. Se non desidera rispondere ad alcune domande, può semplicemente dirlo.**

**è d’accordo a partecipare a questo sondaggio che misura le opinioni dei cittadini sul fenomeno delle persone senza dimora?** SI

NO *(andare a sezione III RIFIUTO)*

*[Se si]* **Bene allora, adesso iniziamo!**

*[TESTO PER L’INTERVISTATORE]* **Inizierò facendole delle domande generali riguardo alle persone senza dimora. Prima di tutto, dovrebbe sapere che definiamo l’essere senza dimora anche il fatto di dormire in strada, in una macchina, o vivere in un dormitorio d’emergenza o temporaneo.**

| *[Nota :* ***Mai*** *leggere ad alta voce le opzioni NS or R]* | NS= Non so  R= Rifiuto a rispondere |
| --- | --- |
|  |  |
| 1. **Dunque, lei è mai stato senza fissa dimora?**   *[nota : anche per una notte, conta come SI]* | 1 = SI  2 = NO *(passare a Q4)*  3 = R (*passare a Q4*) |
| 1. *[Se risponde “si” a Q1]:* **Quando è stato?** *[leggere ad alta voce gli items]* | 1 = Negli ultimi 12 mesi  2 = 1-2 anni fa  3 = 3-4 anni fa  4 = 4-5 anni fa  5 = Più di 5 anni fa  6 = NS  7 = R |
| 1. *[Se risponde “si” a Q1]:* **Complessivamente quanto tempo della sua vita è rimasto senza casa?** *[leggere ad alta voce gli items]* | 1 = Meno di una settimana  2 = Meno di un mese  3 = Meno di un anno  4 = Meno di due anni  5 = Meno di quattro anni  6 = Più di quattro anni  7 = NS  8= R |
| 1. **E’ mai capitato che qualche membro della sua famiglia, amico o conoscente fosse senza dimora?** | 1 = SI  2 = NO *(passare a Q6)*  3 = NS (*passare a Q6)*  4 = R (*passare a Q6)* |
| 1. *[Se famigliari o amici sono mai stati senza dimora = si]* **Si trattava di …** | 1 = Genitore*[padre, madre]*  2 = Figlio/a  3 = Fratello/sorella  4 = Marito/moglie/compagno  5 = Amico/a  6 = Altro parente  7 = Conoscente  8 = NS  9 = R |
| 1. **Potrebbe dirmi approssimativamente quante persone senza dimora ci sono, in Italia?**   *[l’intervistato dovrebbe dare un numero- non una percentuale]* | /_______________/ |
| 1. **Secondo lei, qual è la percentuale di persone senza dimora con...**   *[l’intervistato dovrebbe dare una percentuale]* |  |
| 1. Problemi mentali? | _______% |
| 1. Problemi di dipendenza (alcool, droghe)? | _______% |
| 1. **In Italia, chi finanzia la maggior parte dei servizi sociali per le persone senza dimora**?   *[è ammessa solo una risposta]* | 1 = Governo  2 = Organizzazioni Non Governative/Beneficenza  3 = Chiese e comunità religiose  4 = NS  5 = R |
| 1. **In Italia, chi finanzia la maggior parte dell’assistenza sanitaria per le persone senza dimora?**   *[è ammessa solo una risposta]* | 1 = Governo  2 = Organizzazioni Non Governative/Beneficenza  3 = Chiese e comunità religiose  4 = NS  5 = R |
| 1. **Nella zona in cui vive, direbbe che ci sono molte,** **alcune, poche o nessuna persona senza dimora?**   *[Nella zona in cui vive = Nel suo quartiere]* | 1 = Molte persone  2 = Alcune persone  3 = Poche persone  4 = Nessuna (*Passare a Q12)*  5 = NS  6 = R |
| 1. **In media, quante persone senza dimora diverse vede a settimana?**   *[Se l’intervistato vede la stessa persona diverse volte a settimana, conta come 1 persona]* | 1 = Nessuna  2 = 1 o 2  3 = da 3 a 10  4 = Più di 10  5 = NS  6 = R |
| 1. **Le autorità pubbliche dovrebbero considerare il fenomeno delle persone senza dimora come una priorità?** | 1 = SI  2 = NO  3 = NS  4 = R |
| 1. **Quando Lei passa vicino a una persona senza dimora, si comporta con cautela ?** | 1= Spesso  2= A volte  3= Raramente  4= Mai  5= NS  6 =R |
| 1. **Nell’ultimo anno, le è mai capitato di …**   *[leggere ad alta voce gli items]* | 1 = SI  2 = NO  3 = NS  4 = R |
| 1. Dare soldi, cibo, vestiti a una persona senza dimora | 1 2 3 4 |
| 1. Dare soldi, cibo, vestiti a un’organizzazione di beneficenza o no-profit per persone senza dimora | 1 2 3 4 |
| 1. Prestare qualsiasi tipo di servizio volontario in un’organizzazione di beneficenza o no-profit per persone senza dimora | 1 2 3 4 |
| 1. Altro*[specificare]*: ___________________ | 1 2 3 4 |
| 1. **Per ridurre il fenomeno delle persone senza dimora sarebbe disposto a** *[leggere ad alta voce gli items]***?** | 1 = SI  2 = NO  3 = NS  4 = R |
| 1. Pagare più tasse? | 1 2 3 4 |
| 1. Fare volontariato? | 1 2 3 4 |
| 1. Avere un dormitorio per persone senza dimora vicino a casa sua? | 1 2 3 4 |

**Modulo *Disponibilità a pagare***

*[Nota:* ***Mai*** *leggere ad alta voce le opzioni NS o R]*

| [Teso per l’intervistatore]  Adesso, ci piacerebbe sapere la sua opinione riguardo a diversi interventi per ospitare persone senza dimora.  Sappia che in Italia:  50.724 persone sono senza dimora; Circa il 10% soffre di dipendenza (alcol o droghe) e/o soffre di una grave malattia mentale.  Le soluzioni attuali sono ripari di emergenza o ripari temporanei.  Una persona senza dimora può rimanere fino a una settimana nei ripari di emergenza. Dopo diversi mesi di attesa, può accedere a ripari temporanei, dove spesso dorme in dormitori. Egli può rimanere lì da 3 a 6 mesi se rispetta certe regole quali coprifuoco e non ha problemi di salute mentale o problemi di dipendenza al momento dell’ingresso. Sono disponibili operatori sociali. Coppie e persone con animali domestici non sono ammessi. Un contributo finanziario è richiesto sulla base del reddito.  Nella prossima sezione, le presentiamo un programma di accoglienza abitativa innovativo per le persone senza dimora.  Il suo scopo è di dirci quanto sarebbe disposto a pagare per questo programma. Non vogliamo che stimi il costo effettivo del programma ma che ci dica che valore/utilità darebbe al programma. Tutto questo è ipotetico e solo per scopo di ricerca. Non ci sarà nessun aumento delle tasse!  Può essere disposto a pagare tanto, poco o nulla.  Non c’è una risposta giusta o sbagliata. | | |
| --- | --- | --- |
| **Questo programma innovativo è chiamato *Housing First*.**  Si rivolge a persone senza dimora con problemi di salute mentale o fisica (ad es., malattia mentale, dipendenza da alcol o droghe, disabilità).  Per accedere a questo programma, una persona senza dimora non ha bisogno di sottoporsi ad alcun trattamento per disturbi di salute mentale o problemi di dipendenza.  Hanno case indipendenti e beneficiano di assistenza medica e sociale adeguata ai loro bisogni (fino a 7 giorni a settimana).  Pagano parte del loro affitto, ma in caso di reddito insufficiente, sono disponibili i sussidi.  Un programma simile è attualmente in fase di sperimentazione in Italia e mostra che dopo due anni, la stragrande maggioranza di persone senza dimora (circa 80%) inserite in questo programma rimane nella propria abitazione. | | |
| 1. **Proporrò degli importi in euro. La prego di dirmi che cosa sarebbe disposto a pagare ogni anno tramite tasse per questo programma.**   *[Intervistatore: se la risposta spontanea è “Zero euro” o non voglio pagare, segnare 1 = SI qui.*  *Quindi andare a Q16.2]*  *[Intervistatore: dopo il primo rifiuto o “non so”* ***fermarsi*** *e continuare con la successiva domanda Q 16.1].*  **Sarebbe disposto a pagare ………………………**  *[Se l’intervistato accetta l’offerta più alta* (€400*), andare alla domanda Q 16.1].* | 1 = SI  2 = NO  3= NS  4 =R  0€ == > 1  *(Valuta/cambio sarà adattato allo STATO)*  €10? …… 1 2 3 4 *[83cts/mese]*  €25? …….1 2 3 4 *[2€/ mese]*  €50? …....1 2 3 4 *[4€// mese]*  €100? …...1 2 3 4 *[8€// mese]*  €200? …...1 2 3 4 *[17€// mese]*  €400? …...1 2 3 4 *[33€// mese]* |  |
| 1. **Quale sarebbe il massimo che sarebbe disposto a pagare ogni anno tramite tasse per questo programma?**   *Ricordare all’intervistato l’ultimo importo che ha accettato (e l’importo rifiutato, dove è il caso). L’obiettivo è far si che l’intervistato indichi un massimo tra questi 2 valori].*  *Es: se si a 25 € e no 50 €, chiedere: Tra 25 e50 € quanto sarebbe l’importo massimo ...* | /-----------/ € |  |
| 1. *[Se l’intervistato risponde 0€]* **Per favore, potrebbe dirmi la principale ragione del perché ha rifiutato? È perchè**… | 1 = Il programma non funzionerà  2 = Altri programmi sono più importanti/di maggiore priorità  3 = non voglio pagare più tasse  *(andare a Q18)*  4 = Non posso permettermi di pagare più tasse *(andare a Q18)*  5 = Altro: -------------------------------  6= NS  7 =R |  |
| 1. **Fino ad ora vi ho parlato di un programma per persone senza dimora con problemi di salute mentale o dipendenza. Sarebbe disposto a pagare (di più, lo stesso, o meno) ogni anno tramite tasse se il programma fosse APERTO A TUTTE le persone senza dimora?** | 1= di più *(andare a Q17.1)*  2= lo stesso *(andare a Q18)*  3= meno *(andare a Q17.1)*  4= NS  5 = R |  |
| 1. **Specifichi l’importo massimo che sarebbe disposto a pagare ogni anno tramite tasse per questo programma?** | /----------/ € |  |
| 1. **Quanto è sicuro della sua risposta?** | 1= assolutamente sicuro  2= relativamente sicuro  3= relativamente insicuro  4 = assolutamente insicuro  5= NS  6= R |  |
| 1. **Per l’analisi dei dati, avremmo bisogno di sapere se il suo stipendio è tassato.** | 1 = SI  2= NO  3= NS  4 =R |  |

*[TESTO PER L’INTERVISTATORE].* **La ringrazio per le sue risposte. Adesso, vorrei farle alcune domande riguardo alle condizioni di vita delle persone senza dimora in Italia.**

| 1. **Negli ultimi 3 anni, direbbe che il numero delle persone senza dimora è…** | 1 = Fortemente aumentato  2 = Un po’ aumentato  3 = Un po’ diminuito  4 = Fortemente diminuito  5 = Rimasto lo stesso (spontaneo)  6 = NS  7 = R |
| --- | --- |
| 1. **Leggerò diverse affermazioni sulle persone senza dimora.** *[leggere ad alta voce gli items]* **La prego di dirmi se è …** | 1 = Assolutamente D’ACCORDO  2 = Abbastanza D’ACCORDO  3 = Abbastanza in DISACCORDO  4 = Per niente D’ACCORDO  5= NS  6 =R |
| 1. Le persone senza dimora sono vittime di aggressioni (violenza, rapine, minacce e attacchi). | 1 2 3 4 5 6 |
| 1. Sono vittime di discriminazione nel momento dell’ assunzione. | 1 2 3 4 5 6 |
| 1. Mangiano almeno due pasti al giorno. | 1 2 3 4 5 6 |
| 1. Sono in grado di rimanere in contatto con famigliari e amici. | 1 2 3 4 5 6 |
| 1. Hanno un’aspettativa di vita più breve rispetto alla popolazione generale. | 1 2 3 4 5 6 |
| 1. Molti rimangono senza dimora per scelta. | 1 2 3 4 5 6 |
| 1. La maggior parte ha competenze lavorative. | 1 2 3 4 5 6 |
| 1. Potrebbero prendersi cura di una casa se ce l’avessero (tenere pulito, decorare). | 1 2 3 4 5 6 |
| 1. Hanno accesso a un lavoro retribuito o non retribuito (volontariato, stage etc.). | 1 2 3 4 5 6 |
| 1. La loro principale fonte di reddito proviene da sussidi sociali. | 1 2 3 4 5 6 |
| 1. Trascorrono molto del loro tempo da sole, fuori da qualsiasi rete sociale. | 1 2 3 4 5 6 |
| 1. **Secondo la sua opinione, i servizi forniti............ incontrano i bisogni delle persone senza dimora:** | 1 = Assolutamente D’ACCORDO  2 = Abbastanza D’ACCORDO  3 = Abbastanza in DISACCORDO  4 = Per niente D’ACCORDO  5= NS  6 =R |
| 1. Negli ospedali o pronto soccorso | 1 2 3 4 5 6 |
| 1. Da medici di base e ambulatori specialistici | 1 2 3 4 5 6 |
| 1. Rifugi di emergenza   *[Intervistatore se richiesto: durata massima del soggiorno:7 giorni]* | 1 2 3 4 5 6 |
| 1. Rifugi di transizione   *[Intervistatore se richiesto: durata massima del soggiorno:3 a 6 mesi]* | 1 2 3 4 5 6 |
| 1. Il programma Housing first, se comparato ai tradizionali servizi di ricovero, risponde meglio ai bisogni delle persone senza dimora ? | 1 2 3 4 5 6 |
| 1. **Secondo la sua opinione, quali sono le TRE ragioni che meglio spiegano perché le persone rimangono senza dimora?**   *[Nota: attendere la risposta spontanea e segnare la risposta più simile].* | 1 = Perdita del lavoro/ periodo di disoccupazione  2 = Reddito insufficiente /non riescono a pagare un affitto  3= Casa distrutta da una catastrofe (incendio, inondazione..)  4 = Eccessivamente indebitate  5 = Malati o disabili  6 = Dipendenza (alcol, droghe o altri tipi di dipendenza)  7 = Separazione, divorzio o perdita di un membro della famiglia  8 = Problemi di malattia mentale  9 = Nessun accesso a sussidi sociali o servizi di supporto  10 = Immigrazione clandestina  11 = Propria scelta  12 = Altro 1:___________________________________  13 = Altro 2:___________________________________  14 = Altro 3:___________________________________  15 = Niente *(SPONTANEA)*  16= NS  17 =R |
| 1. **Secondo la sua opinione, chi dovrebbe essere il principale responsabile nel provvedere ai ricoveri D’EMERGENZA per le persone senza dimora?** | 1 = Governo  2 = Organizzazioni Non-Governative/Beneficenza  3 = Chiese e comunità religiose  4 = Le persone senza dimora stesse  5= NS  6 = R |
| 1. **Secondo la sua opinione, chi dovrebbe essere il principale responsabile nel provvedere ad ABITAZIONI A LUNGO TERMINE per le persone senza dimora?** | 1 = Governo  2 = Organizzazioni Non-Governative/Beneficenza  3 = Chiese e comunità religiose  4 = Le persone senza dimora stesse  5= NS  6 = R |
| 1. **Quale gruppo di persone senza dimora dovrebbe avere la priorità per un programma di abitazioni a lungo termine?**   *[Domanda aperta, le opzioni non dovrebbero essere lette ad alta voce.: attendere la risposta spontanea e segnare la risposta più simile o se non è elencata, scrivere la risposta sotto “Altro”]* | 1 = Famiglie  2 = Donne  3 = Giovani [<30 anni **senza** bambini]  4 = Persone con problemi di salute mentale  5 = Lavoratori  6 = I più anziani  7 = Persone con disabilità  8 = Disoccupati  9 = Persone con problemi di dipendenza da alcool o droghe  10 = Nessuno  11 =Altro___________________________________  12= NS  13=R |
| 1. **In generale, pensa che il Governo spenda (troppo, abbastanza o troppo poco) nel welfare sociale…** | 1 = Troppo  2 = Abbastanza  3 = Troppo poco  4 = NS  5 = R |
| 1. **Pensa che il Governo spenda (troppo, abbastanza o troppo poco) …… per aiutare le persone senza dimora?** | 1 = TROPPO  2 = ABBASTANZA  3 = TROPPO POCO *(andare a Q30)*  4 = NS  5 = R *(andare a Q30)* |
| 1. **Potrebbe essere più specifico?**   *[Nota: attendere la risposta spontanea e segnare la risposta più simile]* | 1 = I programmi per le persone senza dimora non sono una priorità.  2 = Spreco di fondi perché le politiche sono inefficienti.  3 = Spreco di fondi perché le persone senza dimora non se lo meritano.  4= Il governo spende una cifra adeguata per aiutare le persone senza dimora  5 = Altro___________________________________  6=NS  7 =R |

***[TESTO PER L’INTERVISTATORE]* CARATTERISTICHE Socio-demograFICHE.**

**Infine, Le farò alcune domande per definire il suo profilo socio-demografico.**

| 1. *[SOLO per l’intervistatore, non chiedere all’intervistato!]* | 1= maschio  2=femmina |
| --- | --- |
| 1. **In che anno è nato?**   *[Codificare con* ***4 cifre****. Per esempio: 1953 anni è codificato 1953, 90 è codificato 1990.*  *[Se c’è qualche ESITAZIONE, passare alla domanda dell’età]* | Anno: /---/---/---/---/ |
| - 1. Quanti anni ha? | Età: /----------/ anni |
| 1. **Qual è la sua nazionalità?** *[scrivere lo Stato]* | /_______________________/ |
| 1. **Qual è il suo livello di istruzione più alto?**   *Intervistatore: Attendere la risposta spontanea, non fornire le varie modalità…* | 1 = Laurea Magistrale o Master o Dottorati (formazione universitaria di quattro anni o più).  2 = Laurea triennale o equivalente (fino a 3 anni di scuola dopo le superiori)  3 = Istruzione superiore (fino a 2 anni di formazione dopo le superiori)  4 = Diploma di scuola superiore  5 = Studi superiore interroti  6 = Scuola media  7 = Scuola elementare  8 = Non ho frequentato la scuola  9 = Altra qualificazione  10= NS  11= R |
| 1. **Al momento, qual è la sua situazione lavorativa?**   *[Solo una risposta è ammessa]* | 1 = Lavoro Full time (35 ore o più alla settimana)  2 = Lavoro part time (da 8 a 35 ore alla settimana)  3 = Lavoro part time occasionalmente (meno di 8 ore)  4 = Studente a tempo pieno  5 = Pensionato  6 = Disoccupato  7 = Non sto lavorando per altre ragioni  8 = NS  9 = R |
| 1. **Qual è il reddito annuale lordo della sua FAMIGLIA senza trattenute (Prima del pagamento delle tasse o di altre spese come i contributi, l’assicurazione sanitaria, etc. ?)**   *[Intervistatore: aspettare la risposta spontanea e segnare la risposta appropriata; se rifiuta di fornire l’importo esatto, dovrebbero essere proposta la seguente scala del Reddito Famigliare annuo lordo:]*  *[Intervistatore: se l’intervistato chiede altre informazioni, precisare:]* **Dovrebbe includere tutte le fonti di reddito tra salari, pensioni, sussidi sociali, interessi di risparmio, e rendite.** | /----------------------------------------/€  *(Valuta/cambio sarà adattato allo STATO)*  1= ‘meno di 5 000€’  2= tra 5 000 & 10 000 €  3= tra 10 000 & 15 000’€  4= tra 15 000 & 20 000’€  5= tra 20 000 & 30 000’€  6= tra 30 000 & 40 000’€  7= tra 40 000 & 50 000’€  8= tra 50 000 & 60 000’€  9= tra 60 000 & 70 000’€  10= più di 70 000 €  11 = NS  12 = R |
| 1. **Qual è il suo status civile? Lei è?** | 1 = sposato  2 = vedovo  3 = separato/divorziato  4 = unione civile (unione contrattuale al di fuori del matrimonio)  5 = single |
| 1. **Quanti membri del nucleo familiare vivono con lei ?** | /-------------------/ |
| 1. **Quanti figli ha?** | /------------------/ |
| 1. **Quanti figli a carico (che sono legati al reddito famigliare imponibile) ci sono?** | **/------------------/** |
| 1. **Qual è il suo codice postale?**   *[Se l’intervistato non lo sa o non vuole dare informazioni, chiedere la divisione amministrativa* ***più vicina*** *come provincia (It)…]* | /-----------------/ |
| 1. Provincia | /------------------/ |
| 1. Vive in una… ? | 1 = zona urbana  2 = intermedia (semiurbana)  3 = zona rurale  4 = NS  5 = R |
| 1. **Quanti numeri fissi operativi ha in casa** (non inclusi i numeri di telefono associati che non vengono utilizzati- ad es. solo per internet- o chiamate aziendali)**?** | /------------------/ |
| 1. **Quanti telefoni cellulari ha in casa** (non inclusi quelli usati per chiamate aziendali)? | /------------------/ |
| 1. Lei ha un telefono cellulare? | 1 = Si  2 = No  3 = R |

**FINE DELL’INTERVISTA**

**Ora, abbiamo finito con l’intervista!**

**L’ Università degli Studi di Padova la ringrazia per la partecipazione.**

**Ha qualche domanda?** *[Se si, l’intervistatore dovrebbe fare ogni sforzo per cercare di rispondere].*

*[Se l’intervistatore non può rispondere o è insicuro]* ***Mi scusi. Non ho questa informazione. Tuttavia, può inviare una mail a:*** [***HOME_EU@ispa.pt***](mailto:HOME_EU@ispa.pt)

*[Istruzioni per l’intervistatore: PER FAVORE prendere nota della domanda per la prossima sessione di formazione.]*

*Domande: ------------------------------------------------------------------------------------------------------------------------------------------------------------------------------------------------------------------------------------------------------------------*

**Inoltre, se avesse domande o commenti in seguito, è possibili chiamare il direttore del progetto, Prof. Massimo Santinello**, **dell’Università degli Studi di Padova**.

**Il numero di telefono è:** +39 **0498276520**

**Una breve relazione sui risultati di questo sondaggio una volta completato sarà disponibile al seguente indirizzo:** [**www**](http://www.HOME-EU.ORG).home-eu.org

**Ricordi, la sua partecipazione è anonima. La ringrazio ancora. Buona giornata.**

| 1. *DOMANDE ALL’INTERVISTATORE: L’intervistato ha risposto alla maggior parte delle domande in una maniera ragionevole? Ci sono stati dei problemi che potrebbero rendere i dati ottenuti non validi o discutibili?* | 1 = NESSUN PROBLEMA  2 = POTENZIALI PROBLEMI (descrivere di seguito): _________________________________________________  _________________________________________________  3 = PROBLEMI SERI (descrivere di seguito): _________________________________________________  _________________________________________________ |  |
| --- | --- | --- |

*NOTA PER L’INTERVISTATORE: PER FAVORE ANDARE INDIETRO E RIVEDERE PER ESSERE SICURI CHE GLI ITEMS SIANO COMPLETI E LE RISPOSTE CORRETTE. SE NECESSARIO, RICHIAMARE L’INTERVISTATO E CHIARIRE QUALSIASI PROBLEMA.*

| QUESTIONÁRIO DOS CIDADÃOS  SOBRE A OPINIÃO PÚBLICA ACERCA DAS PESSOAS SEM ABRIGO  E AS SUAS PREFERÊNCIAS |
| --- |
| Número de identificação: /__/__/__/__/__/  Data: (____/____/____)  Entrevistador: _________________________ Código do entrevistador: __________ |

1. ***INTRODUÇÃO***

*[GUIÃO PARA O ENTREVISTADOR]*

**Olá, o meu nome é ------------------------. Estou a ligar de Portugal. A sua morada foi aleatoriamente selecionada para fazer parte de um Estudo Europeu sobre a OPINIÃO DOS PORTUGUESES ACERCA DAS PESSOAS EM SITUAÇÃO DE SEM ABRIGO.**

**Are you 18 or older? Tem 18 anos ou mais?**

*[Se sim, então avance para a parte II DISPONIBILIDADE PARA PARTICIPAR NO ESTUDO]*

*[Se não, pergunte]:* **Posso falar com alguém com 18 anos ou mais velho que esteja em casa agora?**

1. *[Se sim, quando a pessoa vier ao telefone, prossiga dizendo]:* **Olá, o meu nome é ------------------------.** *da Universidade D’AIX em Marselha* **A sua casa foi aleatoriamente selecionada para fazer parte de um Estudo Europeu sobre a OPNIÃO DOS PORTUGUESES ACERCA DA SITUAÇÃO DAS PESSOAS EM SITUAÇÃO DE SEM ABRIGO*.*** *[avance para a parte II DISPONIBILIDADE PARA O ESTUDO]*
2. *[Se não, não houver um adulto em casa então pergunte]:* **Qual seria o melhor horário para contactar essa pessoa em casa?**

[REGISTO PARA NOVA LIGAÇÃO]: **Data: ___________ Horário: __________ Nome (somente o primeiro): ___________________ Obrigada. Voltaremos a ligar.**

[Se não sabe informar ou não quer responder]: **Obrigada. Ligaremos noutro momento.**

1. ***DISPONIBILIDADE PARA A ENTREVISTA***

**Podemos falar durante maximo 20 minutos?**

1. *[Se sim]:* **Obrigada! (**e prossiga para a *secção V* *CONFIGURANDO A ENTREVISTA*)

*[Se não, pergunte]:* **Qual seria o horário mais conveniente para voltarmos a ligar?**

[REGISTO PARA NOVA LIGAÇÃO:] Data: __________ Horário: __________ **Obrigada. Voltaremos a ligar.**

- - *[Se a pessoa não quer ou não sabe dizer, diga]*: **Nós gostaríamos muito de saber as suas opiniões sobre esse assunto. Esta seguro de que não podemos falar agora ou mais tarde?**
    - *[Se a resposta for sim].* **Obrigada! *(****e prossiga para a secção V CONFIGURANDO A ENTREVISTA]*
    - *[Se a resposta for Sim mas num outro momento, registe a data e a hora]*

**Data: _______________ Hora: ______________ Obrigada. Ligaremos novamente.**

- - - *[Se não, prossiga para a secção III RECUSA]*

1. ***RECUSA***
2. **Entendemos que não queira participar; mas poderia ajudar-nos a analisar as recusas dizendo-nos a sua idade, o seu grau de formação e a sua profissão?**

1= Sim

2= Não

**a.Ano de nascimento: ___________________**

**b. Grau mais elevado de ensino que completou:**

1 = Pós-graduação (Mestrado, PhD, etc/ 4 anos ou mais de pós-secundário)

2 = Bacharelado ou equivalente (até 3 anos de pós-secundário)

3 = Frequência do ensino superior (até 2 anos de pós-secundário)

4 = Ensino secundário

5 = Frequentou o secundário

6 = Completou o sexto ano

7 = Escola primária

8 = Não frequentou a escola

9 = Outra qualificação

10= NS Não sabe

11= NA Não aplicável

- 1. **Ocupação :**

1 = Trabalha a tempo inteiro (35 horas ou mais por semana)

2 = Trabalha em *part-time* (8 a 35 horas por semana)

3 = Trabalha ocasionalmente em *part-time* (menos de 8 horas)

4 = Estudante em tempo inteiro

5 = Aposentado

6 = Desempregado

7 = Não trabalha por outros motivos

8 = NS Não sabe

9 = NA Não aplicável

**b.Quantas pessoas vivem na sua casa, incluindo a si próprio(a)? _________**

**c.Quantas crianças com menos de 18 anos vivem na sua casa? _________**

**Obrigado pela sua ajuda e desculpe o incómodo.**

1. ***SE O ENTREVISTADO NÃO FALAR PORTUGUÊS ADEQUADAMENTE.***

*[Nota: Se contactar alguém que fale uma língua estrangeira e cujo português seja obviamente insuficiente para completar a entrevista, diga:]*

***Desculpe, eu só falo português.*** ***Obrigada pelo seu tempo, adeus.***

1. ***CONFIGURAR A ENTREVISTA***

*[GUIÃO PARA O ENTREVISTADOR]*

**Este estudo por telefone faz parte de um estudo mais alargado a decorrer em 8 países membros da União Europeia. O objetivo é avaliar a opinião dos cidadãos a respeito das pessoas em situação de sem abrigo e entender as suas expectativas acerca do assunto. Por favor tenha em atenção que durante essa entrevista a sua opinião se reporte apenas a situação em Portugal. As suas respostas permanecerão estritamente anonimas. Eu não lhe perguntarei o seu nome ou o seu sobrenome. Se não desejar responder a algumas questões sinta-se à vontade para o dizer.**

**Concorda em participar dessa pesquisa avaliando a opinião dos cidadãos acerca das pessoas em situação de sem abrigo.**

SIM

NÃO (prossiga para a secção III RECUSA)

[Se sim] **Tudo certo, então, vamos começar!**

[*GUIÃO PARA O ENTREVISTADOR*] **Vou começar por fazer perguntas gerais acerca das pessoas em situação de sem abrigo. Em primeiro lugar, informamo-lo(a) que definimos as pessoas em situação de sem abrigo como as pessoas que dormem nas ruas, nos carros, que vivem em abrigos de emergência ou alojamentos temporários.**

| *[Nota: Nunca leia as modalidades Não sabe ou Recusa responder.* | NS= Não sabe (NS)  R= Recusa responder |
| --- | --- |
|  |  |
| **1.Então, já passou por uma situação de sem-abrigo?**  *[nota: até mesmo uma noite, conta como SIM]* | 1= SIM  2= NÃO *(avance para a Q4)*  3 = R *(avance para a Q4)* |
| **2.** *[Se a resposta Q1 foi “sim”]:* **Quando foi?** *[leia os itens]* | 1 = Nos últimos 12 meses  2 = Há 1-2 anos  3 = Há 3-4 anos  4 = Há 4-5 anos  5 = Há mais que 5 anos  6 = NS  7 = R |
| *3.[Se a resposta Q1 foi “sim”]:* **Por quanto tempo esteve na situação de sem abrigo na sua vida?** *[leia os itens]* | 1 = Menos de uma semana  2 = Menos de um mês  3 = Menos de um ano  4 = Menos de dois anos  5 = Menos de quarto anos  6 = Mais de quarto anos  7 = NS  8= R |
| **4. Algum membro da sua família, amigo ou conhecido esteve numa situação de sem abrigo?** | 1 = SIM  2 = NÃO *(avance para Q6)*  3 = NS *(avance para Q6)*  4 = R *(avance para Q6)* |
| ***5.*** *[Se “sim”, algum familiar ou amigo alguma vez passou pela situação de sem abrigo]*  **Foi um(a)…** | 1 = *Pais [mãe/pai]*  2 = Filho(a)  3 = Irmão/irmã  4 = Marido/Mulher/Parceiro(a)  5 = Amigo(a)  6 = Outro familiar  7 = Conhecido  8 = NS  9= R |
| **6.Consegue dizer-me, aproximadamente, quantas pessoas em situação de sem abrigo existem em Portugal?**  *[o entrevistado deve dizer um número e não uma percentagem]* | /_______________/ |
| **7. Na sua opinião, qual é a percentagem de pessoas em situação de sem-abrigo com…**  **[o entrevistado deve dar a percentagem]** |  |
| 1. Doenças mentais? | _______% |
| 1. Problemas de adicção (álcool e drogas)? | _______% |
| **8**. **Em Portugal, quem financia a maior parte dos serviços sociais para os sem-abrigo?**  *[Somente uma resposta pode ser dada]* | 1 = Governo  2 = Organizações Não-governamentais e de Beneficência  3 = Igrejas e comunidades religiosas  4 = NS  5 = R |
| **9. Em Portugal, quem financia a maior parte das estruturas de cuidados de saúde para pessoas sem-abrigo?**  *[somente uma resposta é aceite]* | 1 = Governo  2 = Organizações Não-governamentais e de Beneficência  3 = Igrejas e comunidades religiosas  4 = DK NS  5 = R |
| **10. Na zona em que vive, saberia dizer se existem muitas, algumas, poucas ou nenhuma pessoa em situação de sem-abrigo?**  *[A zona em que vive= o seu bairro]* | 1 = Muitas pessoas  2 = Algumas pessoas  3 = Poucas pessoas  4 = Nenhuma (avance para Q12)  5 = NS  6 = R |
| **11. Em média, quantos sem-abrigo diferentes vê por semana?**  *[Se o entrevistado vê a mesma pessoas diversas vezes durante a semana, conta como apenas 1]* | 1 = Nenhuma  2 = 1 OU 2  3 = 3 A 10  4 = Mais de 10  5 = NS  6 = R |
| **12. As autoridades públicas deveriam considerar a situação das pessoas sem-abrigo como uma prioridade?** | 1= SIM  2= NÃO  3= NS  4= R |
| **13. Quando passa junto a um sem-abrigo é cauteloso?** | 1= Sempre  2= Às vezes  3= Raramente  4= Nunca  5= NS  6=R |
| **14.Over the past year, have you …**  *[read out items]*  **Durante o último ano…**  *[leia os itens]* | 1 = YES SIM  2 = NO NÃO  3 = DK NS  4 = R |
| 1. Deu dinheiro, comida ou roupa a pessoas em situação de sem-abrigo | 1 2 3 4 |
| 1. Deu dinheiro, comida ou roupas para organizações de beneficência ou organizações sem fins lucrativos para pessoas em situação de sem-abrigo. | 1 2 3 4 |
| 1. Fez algum voluntariado em organizações sem fins lucrativos ou de beneficência para pessoas em situação de sem-abrigo. | 1 2 3 4 |
| 1. Outros [especifique]: ___________________ | 1 2 3 4 |
| **15. Para reduzir a quantidade de pessoas em situação de sem-abrigo estaria disposto a** *[leia os itens]*? | 1 = SIM  2 = NÃO  3 = NS  4 = R |
| 1. Pagar mais impostos? | 1 2 3 4 |
| 1. Fazer voluntariado? | 1 2 3 4 |
| 1. Ter um abrigo para os de sem-abrigo perto da sua casa? | 1 2 3 4 |

**Module *Willingness to pay Módulo Disponibilidade para pagar***

*[Nota: Nunca leia as modalidades NS ou R]*

| [Guião para o Entrevistador]  Agora, gostaríamos de saber sua opinião sobre diferentes intervenções para proporcionar habitação a pessoas em situação de sem-abrigo.  Sabia que em Portugal:  Existem cerca de cinco mil pessoas sem abrigo; Cerca de 60% sofrem de adições (álcool ou drogas) e 30% sofrem de doença mental grave.  As soluções atuais são abrigos de emergência ou alojamentos de transição.  Uma pessoa em situação de sem-abrigo pode ficar até uma semana em abrigos de emergência. Depois, pode ter acesso a abrigos de transição, onde muitas vezes dorme em dormitórios, podendo ficar de 3 a 6 meses se obedecer a regras como toques de recolher e se não tiver problemas de saúde mental ou problemas de adicção de álcool na entrada. Assistentes sociais estão disponíveis. Casais e pessoas com animais de estimação não são permitidos. É solicitada uma contribuição financeira com base nos rendimentos.  Na próxima secção, apresentaremos um programa habitacional inovador para pessoas em situação de sem abrigo.  Propomos que nos diga quanto estaria disposto(a) a pagar por este programa. Não queremos que estime o custo total do programa, mas o valor / utilidade que coloca no programa. É um exercício hipotético para fins de investigação. Não haverá aumento de impostos!  Estaria disposto a pagar muito, pouco ou nada.  Não há respostas certas ou erradas. | | |
| --- | --- | --- |
| **Este programa inovador é chamado Casas Primeiro, também conhecido por “Housing First”.**  Destina-se a pessoas em situação de sem-abrigo com problemas de saúde mental ou física (isto é, doença mental, adicção a álcool ou drogas, deficiência).  Para ter acesso a esse programa, a pessoa em situação de sem-abrigo não precisa se submeter a qualquer tratamento para problemas de saúde mental ou problemas de adicção.  Têm acesso a alojamento individual e beneficia de assistência médica e social adaptada às suas necessidades (7 dias por semana).  As pessoas pagam parte do aluguer, mas em caso de rendimento insuficiente, os subsídios estão disponíveis.  Um programa deste género está a decorrer em Portugal e mostra que após dois anos, a grande maioria das pessoas em situação de sem-abrigo (cerca de 86%) permanece na sua habitação. | | |
| **16. Vou propor montantes em euros. Por favor, diga me o que estaria disposto a pagar anualmente através dos impostos para este programa.**  *[Entrevistador: se a resposta espontânea for "Zero euro" ou não quiser pagar, relate 1 = SIM aqui.*  *Em seguida, vá para Q16.2]*    *[Entrevistador: após a primeira RECUSA ou "NÃO SABE" parar e continuar com a próxima pergunta Q16.1].*  **Estaria disposto(a) a pagar ...........................**    *[Se o entrevistado aceitar a oferta mais alta (€ 400), vá para a pergunta Q16.1]* | 1 = SIM  2 = NÃO  3= NS  4 =R  0€ == > 1  €10? …… 1 2 3 4 *[83cts/mês]*  €25? …….1 2 3 4 *[2€/ mês]*  €50? …....1 2 3 4 *[4€// mês]*  €100? …...1 2 3 4 *[8€// mês]*  €200? …...1 2 3 4 *[17€// mês]*  €400? …...1 2 3 4 *[33€// mês]* |  |
| 1. **Qual seria o máximo que você estaria disposto à pagar em cada ano através de impostos para este programa?**   *[Lembre o entrevistado o último valor que aceitaram (e o montante que recusaram, quando aplicável). O objetivo é fazer com que o entrevistado indique um máximo entre esses 2 valores].*  *Ex: se sim a 25 € e não 50 €, pergunte: entre 25 e 50 € o que seria o montante máximo...* | /-----------/ € |  |
| *16.2 [Se o entrevistado respondeu 0 €]***: Por favor, poderia dizer-me o principal motivo pelo qual se recusou? É porque...** | 1 = O programa não irá funcionar  2 = Outros programas são mais importantes/ têm prioridade  3 = Eu não quero pagar mais impostos *(vá para Q18)*  4 = Eu não posso pagar mais impostos *(vá para Q18)*  5 = Outro:__________________  6= NS  7 =R |  |
| 1. **Até agora falei sobre um programa para pessoas em situação de sem-abrigo com doenças mentais ou problemas de adição. Você estaria disposto a pagar (mais, o mesmo, ou menos) em cada ano através de impostos se este programa fosse aberto A TODAS as pessoas em situação de sem-abrigo?** | 1= Mais *(avance para Q17.1)*  2= Mesmo *(avance para Q18)*  3= Menos *(avance para Q17.1)*  4= NS  5 = R |  |
| 17.1 **Especifique o valor máximo que estaria disposto a pagar todos os anos através de impostos para este programa?** | /----------/ € |  |
| 1. **Quão confiante está da sua resposta?** | 1= Certeza absoluta  2= Relativamente certo(a)  3= Mais ou menos certo(a)  4= Relativamente inseguro  5 =Absolutamente inseguro |  |
| 1. **Para análise de dados, precisamos saber se seus rendimentos são tributáveis.** | 1 = SIM  2= NÃO  3= NS  4 =R |  |

*[GUIÃO DO ENTREVISTADOR].* **Obrigada pelas suas respostas. Agora, gostaria de lhe fazer algumas perguntas acerca das condições de vida das pessoas em situação de sem abrigo em Portugal.**

| **20.Nos últimos 3 anos, diria que o número de pessoas em situação de sem-abrigo tem…** | 1 = Aumentado fortemente  2 = Aumentado um pouco  3 = Diminuído um pouco  4 = Diminuído fortemente  5 = Permaneceu o mesmo  6 = NS  7=R |
| --- | --- |
| **21. Vou ler-lhe várias afirmações a respeito das pessoas em situação de sem-abrigo.**  **[Leia os itens] Por favor, diga-me se…** | 1 = Concorda plenamente  2 = Concorda em parte  3 = Discorda em parte  4 = Discordo plenamente  5= NS  6 =R |
| 1. As pessoas em situação de sem-abrigo são vítimas de assaltos (violência, roubo, ameaças e ataques). | 1 2 3 4 5 6 |
| 1. São discriminados na contratação para um emprego. | 1 2 3 4 5 6 |
| 1. Comem pelo menos duas refeições por dia. | 1 2 3 4 5 6 |
| 1. São capazes de manter contato com a família e amigos. | 1 2 3 4 5 6 |
| 1. Têm uma expectativa de vida mais curta do que a população em geral. | 1 2 3 4 5 6 |
| 21.6 Muitos permanecem em situação de sem-abrigo por opção. | 1 2 3 4 5 6 |
| 21.7 A maioria tem capacidades para o trabalho. | 1 2 3 4 5 6 |
| 21.8 Poderiam cuidar (manter limpa, decorar) uma casa se tivessem uma. | 1 2 3 4 5 6 |
| 21.9 Tem acesso a trabalho remunerado ou não remunerado (voluntariado, estágio, etc.). | 1 2 3 4 5 6 |
| 21.10 A sua principal fonte de rendimento provém de benefícios sociais | 1 2 3 4 5 6 |
| 21.11 Passam grande parte do seu tempo sozinhos, isolados de qualquer rede social | 1 2 3 4 5 6 |
| **22. Na sua opinião, os serviços prestados............. respondem às necessidades das pessoas em situação de sem-abrigo:** | 1 = Concordo plenamente  2 = Concordo em parte  3 = Discordo em parte  4 = Discordo plenamente  5= NS  6=R |
| - 1. Nos hospitais e emergências | 1 2 3 4 5 6 |
| 22.2 Pelos clínicos gerais e especialistas em ambulatório. | 12 3 4 5 6 |
| 22.3 Nos abrigos de emergência  [*Investigador se questionado: um lugar onde as pessoas podem ficar numa duração máxima de estadia 7 dias]* | 1 2 3 4 5 6 |
| 22.4 Nos abrigos de transição  *[Investigador se questionado: um lugar onde as pessoas podem ficar numa duração máxima de estadia: 3 a 6 meses]* | 1 2 3 4 5 6 |
| 22.5 O programa Housing First, responde melhor as necessidades das pessoas quando comparado aos serviços de abrigo? | 1 2 3 4 5 6 |
| **23. Na sua opinião, quais são as TRÊS razões que melhor explicam o motivo das pessoas ficarem sem-abrigo?**    *[Nota: espere pela resposta espontânea e marque aquela que mais se aproxima da resposta].* | 1 = perda de emprego / período de desemprego  2 = rendimento insuficiente / não pode pagar um aluguer  3 = casa destruída por uma catástrofe (incêndios, inundações, etc.)  4 = sobre-endividado  5 = doente ou incapacitado  6 = dependência (álcool, drogas ou outros tipos de dependência)  7 = separação, divórcio ou perda de membro da família  8 = problemas de saúde mental  9 = falta de acesso a benefícios sociais ou serviços de apoio  10 = imigração ilegal  11 = escolha própria  12 = Outro 1: _____________________________________  13 = Outro 2: _____________________________________  14 = Outro 3: ____________________________________  15 = Nenhum (ESPONTÂNEO)  16 = NS  17 = R |
| **24. Na sua opinião, quem deve ser o principal responsável por proporcionar ABRIGO de EMERGÊNCIA para pessoas em situação de sem abrigo?** | 1 = Governo  2 = Organizações Não Governamentais / Instituições de Beneficência  3 = Igrejas e comunidades religiosas  4 = As próprias pessoas em situação de sem-abrigo  5 = NS  6 = R |
| **25. Na sua opinião, quem deve ser o principal responsável por proporcionar HABITAÇÃO DE LONGO PRAZO para pessoas em situação de sem-abrigo?** | 1 = Governo  2 = Organizações Não Governamentais / Instituições de Beneficência  3 = Igrejas e comunidades religiosas  4 = As próprias pessoas em situação de sem-abrigo  5 = NS  6 = R |
| **26. A que grupo de pessoas em situação de sem-abrigo deve ser dada prioridade para um programa de habitação a longo prazo?**  *[Pergunta aberta, as modalidades não devem ser lidas em voz alta.*  *Aguarde a resposta e verifique a resposta mais próxima ou, se não estiver listada, escreva a resposta em "outro"]* | 1 = Famílias  2 = Mulheres  3 = Jovens [<30 anos sem filhos]  4 = Pessoas com transtornos mentais  5 = Empregados  6 = Idosos  7 = Pessoas com deficiência  8 = Desempregados  9 = Pessoas com problemas de dependência de álcool ou drogas  10 = Nenhum   1. = Outro _________________________________   12=NS  13=R |
| **27. Em geral, acha que o governo gasta (demais, o suficiente ou muito pouco) no bem-estar social ...** | 1 = Demasiado  2 = Suficiente  3 = Muito pouco  4 = NS  5 = R |
| **28. Acha que o governo gasta (demais) ou suficiente ou muito pouco) ...... para ajudar as pessoas em situação de sem-abrigo?** | 1 = Demais  2 = Suficiente  3 = Muito pouco (avance para Q30)  4= NS  5= R (avance para Q30) |
| **29. Poderia ser mais específico?**  *[Nota: espere resposta espontânea e marque aquela que mais se aproxima da resposta]*  **É porque ...** | 1 = Os Programas para pessoas em situação de sem-abrigo não são uma prioridade  2 = Desperdício de fundos porque as políticas são ineficientes.  3 = Desperdício de fundos porque as pessoas em situação de sem-abrigo não o merecem  4 = O governo gasta uma quantidade "adequado" para os sem-abrigo  5 = Outro  6=NS  7=R |

**[GUIÃO PARA O ENTREVISTADOR] CARACTERÍSTICAS SOCIO-DEMOGRÁFICAS.**

**Para terminar vou fazer algumas perguntas para definir o seu perfil sócio-demográfico.**

| ***30.*** *[Para o entrevistador SOMENTE, não pergunte ao entrevistado!]* | 1= masculino  2 = feminino |
| --- | --- |
| ***31. Em que ano nasceu?***  *[Código com 4 dígitos. Por exemplo: 1953 anos é codificado 1953, 90 é codificado como 1990.*  *[Se houver alguma HESITAÇÃO, pergunte a idade]* | Ano: /---/---/---/---/ |
| 31.1 Qual é a sua idade? | Idade: Anos |
| **32. Qual é a sua nacionalidade?** *[escreva o país]* | /_______________________/ |
| **33.Qual foi o grau mais alto que alcançou em termos de educação?**  *Entrevistador: A resposta espontânea é esperada, não forneça a escala das modalidades...* | 1 = Ensino Superior (Mestrado e Doutoramento, etc. 4 anos ou mais ou pós-secundário)  2 = Ensino Superior (até 3 anos de estudos pós-secundários)  3 = Ensino superior (até 2 anos de estudos pós-secundários)  4 = Diploma do Secundário  5 = Ensino Médio  6 = Escola secundária  7 = Escola primária  8 = Não frequentou a escola  9 = Outras qualificações  10 = NS  11 = R |
| **34.No momento, qual é a sua situação de trabalho?**  *[Somente uma resposta é esperada]* | 1 = Trabalho a tempo inteiro (35 horas ou mais a semana)  2 = Trabalho a tempo parcial (8 a 35 horas por semana)  3 = Trabalho ocasionalmente a tempo parcial (menos de 8 horas)  4 = Estudante em tempo integral  5 = Aposentado  6 = Desempregado  7 = Não trabalha por outras razões  8 = NS  9 = R |
| **35. Qual é o rendimento bruto anual na sua casa antes de quaisquer descontos (antes de pagar a renda ou outros impostos, seguro de saúde, etc?)**  *[Entrevistador: espere uma resposta espontânea e marque a resposta apropriada; Se a pessoa se recusar a fornecer o montante exacto, deve ser proposta a seguinte escala anual bruta de renda familiar:]*  *[Entrevistador: se o entrevistado pedir informações adicionais, diga:]* **Deve incluir todas as fontes de rendimento, incluindo salários, pensões, benefícios sociais, juros sobre poupanças e anuidades.** | /----------------------------------------/€  *EUROS*  1= ‘menos que 5 000€’  2= entre 5 000 & 10 000’€  3= entre 10 000 & 15 000’€  4= entre 15 000 & 20 000’€  5= entre 20 000 & 30 000’€  6= entre 30 000 & 40 000’€  7= entre 40 000 & 50 000’€  8= entre 50 000 & 60 000’€  9= entre 60 000 & 70 000’€  10= ’mais que 70 000 €  11 = NS   1. = R |
| **36. Qual é o seu estado civil? Está?** | 1 = casado  2 = viúvo  3 = separado / divorciado  4 = Em união de facto (união contratual fora do casamento)  5 = solteiro |
| **37. Incluindo a si mesmo(a), quantas pessoas vivem na sua casa?** | /-------------------/ |
| **38. Quantos filhos tem?** | /------------------/ |
| **39. Quantos filhos dependentes (ligados ao rendimento familiar tributável)?** | **/------------------/** |
| **40.Qual é o seu código postal?**  *[Se o entrevistado não sabe ou não deseja dar a informação, peça a divisão administrativa mais próxima distrito / concelho ...]* | /-----------------/ |
| 1. Distrito |  |
| 1. Quer dizer que você vive ... | 1 = em áreas urbanas  2 = em áreas semi-urbanas  3 = em áreas rurais  4 = NS  5 = R |
| **41. Quantos telefones fixos operacionais tem na sua casa? (**não incluir números de telefone atribuídos que não são utilizados, p.e. para Internet ou negócios)? | /------------------/ |
| **42. Quantos telemóveis tem na sua casa** (não incluindo telefones de serviço)? | /------------------/ |
| 42.1 Tem telemóvel? | - 1. = Sim   2. = Não   3. = R |

**FIM DA ENTREVISTA**

**Completamos a nossa entrevista!**

**Em nome do ISPA – IU em Portugal e da Universidade de Aix’ em Marselha agradecemos a sua participação**

**Tem alguma pergunta?** *[Se sim, o entrevistador deve fazer todos os esforços para tentar responder.]*

*[Se o entrevistador não pode responder ou não tem certeza]* **Sinto muito. Eu não tenho essa informação. No entanto, pode enviar um e-mail para HOME_EU@ispa.pt**

*[Instrução para entrevistador: POR FAVOR anote a pergunta para a próxima sessão de formação.]*

**Questões: ------------------------------------------------ -------------------------------------------------- -------------------------------------------------- -------------------------------------------------- --------------------------------------------------**

**Se tiver alguma questão ou comentário mais tarde pode contactar o Investigador Principal em Portugal, o Prof. José Ornelas no ISPA – IU em Lisboa**

**O telefone é: +351 21 881 17 00**.

**Um relatório síntese deste questionário europeu uma vez terminado será disponibilizado no site com o seguinte endereço:** [**www.HOME.EU.ORG**](http://www.HOME.EU.ORG).

**Relembramos que a sua participação é anónima. Agradecemos novamente. Tenha um bom dia**

| ***43.*** *PERGUNTA PARA O ENTREVISTADOR: O entrevistado respondeu à maioria das perguntas de maneira razoável? Houve algum problema que possa tornar os dados obtidos inválidos ou questionáveis?* | 1 = SEM PROBLEMAS  2 = POTENCIAIS PROBLEMAS (descreva abaixo): _________________________________________________  _________________________________________________  3 = PROBLEMAS SÉRIOS (descreva abaixo):  _________________________________________________  _________________________________________________ |  |
| --- | --- | --- |

*NOTA PARA O ENTREVISTADOR: FAVOR VOLTAR AO INÍCO E REVER O QUESTIONÁRIO PARA ASSEGURAR QUE OS ITENS ESTÃO COMPLETOS E MARCADOS CORRECTAMENTE. SE NECESSÁRIO, LIGAR AO ENTREVISTADO PARA CLARIFICAR QUALQUER PROBLEMA.*

| MEDBORGARUNDERSÖKNING OM  ALLMÄNHETENS ÅSIKTER OCH FÖRVÄNTNINGAR ANGÅENDE  HEMLÖSHET |
| --- |
| IDENTIFIKATIONS NUMMER: /__/__/__/__/__/  DATUM: (____/____/____)  INTERVJU START TID : (____:____) SLUTTID: (____:____)  INTERVJUARE : _________________________ INTERVJUARKOD : __________ |

1. ***INTRODUKTION***

*[INTERVJUARENS MANUS]*

**Hej, Jag heter ----------------------------------. Jag ringer på** *uppdrag Karolinska Institutet* **Ditt hushåll har slumpmässigt valts ut att delta i en Europeisk undersökning om svenskarnas åsikter om hemlöshet.**

**Är du 18 år eller äldre?**

*[Om ja, gå vidare till del II TILLGÄNGLIG FÖR UNDERSÖKNINGEN]*

*[Om inte, fråga]:* ***Kan jag få tala med någon I hushållet som är 18 år eller äldre nu?***

1. *[Om ja, fortsätt på följande sätt när den personen kommer till telefon]:* **Hej, mitt namn är ----------------------------------. Jag ringer från** *(Karolinska Institutet***~~.~~ Ert hushåll har blivit slumpmässigt utvalt för att delta I en Europeisk undersökning om ALLMÄNHETENS ÅSIKTER OM HEMLÖSHET.** *[Och gå sedan vidare till del II TILLGÄNGLIG FÖR UNDERSÖKNINGEN]*
2. *[Om inte någon vuxen person är hemma fråga*] **När vore en lämplig tidpunkt att återkomma?**

[LOG FÖR ÅTERKOPPLING]: Datum: _______ Tidpunkt: ________NAMN ________ **Tack, vi ringer tillbaks då.**

1. [om de inte vet:]: **Tack, vi provar vid något annat tillfälle.**
2. ***Tillgänglig för undersökningen***

**Kan vi prata ca 20 minuter?**

1. [*Om ja]:* **Tack!** *(och fortsätt till sektion V* *PÅBÖRJANDET AV INTERVJUEN)*
2. *[Om inte, fråga*:] **När vore en lämplig tid att ringa tillbaka?**

[*LOG FÖR ÅTERKOPPLING*:] Datum:__________ Tid: _______________ **Tack, vi ringer tillbaks senare.**

- - *[om inte eller vet inte, säg*:] **Vi skulle verkligen vilja ha dina åsikter I den här frågan. Är du säker på att du inte kan nu eller vid ett senare tillfälle?**
    - *[Om svaret blir ja].* **Tack så mycket!** *(och gå vidare till sektion V PÅBÖRJANDET AV INTERVJUEN]*
    - [*Om svaret blir ja men vid ett senare tillfälle notera tidpunkt och dag:]*

Datum:_______________ Tid: _______________ **Tack så mycket, vi hör av oss senare.**

- - - *[Om nej, gå till sektion VÄGRAR DELTA].*

1. ***VÄGRAR DELTA***
   1. **Vi förstår att du inte vill delta, skulle du kunna hjälpa oss förstå varför du inte vill delta, skulle du ändå kunna svara på tre frågor: hur gammal du är, din högsta utbildning och din arbetsstatus**

1 = Ja

2 = Nej

- 1. **Födelseår: ____________________**
  2. **Högsta utbildningsnivå :**

1 = Eftergymnasial utbildning (doktorerat, eller liknande, upp till 4 års eftergymnasial utbildning eller högre)

2 = Kandidatexamen eller Högskoleutbildning motsvarande upp till 3 års eftergymnasial utbildning

3 = Eftergymnasial utbildning (upp till 2 års eftergymnasial utbildning)

4 = Gymnasiet

5 = Högstadiet

6 = mellanstadiet

7 = lågstadiet

8 = har inte gått I skolan

9 = Andra kvalifikationer/utländsk examen

10= DK (Vet inte)

11= NA (Svarar inte)

- 1. **Sysselsättningsstatus :**

1 = Arbetar heltid (35 timmar eller mer I veckan)

2 = Arbetar deltid (8 till 35 timmar i veckan)

3 = Arbetar deltid då och då (mindre än 8 timmar)

4 = Heltidsstuderande

5 = Pensionär

6 = Arbetslös

7 = Arbetar inte av andra skäl

8 = DK (Vet inte)

9 = NA (Svarar inte)

- 1. **Hur manga bor I ert hushåll, inkluderande dig? ____**
  2. **Hur många barn under 18 år bor I hushållet? ______**

**Tack för din hjälp och ber om ursäkt för att jag stört dig.**

1. ***RESPONDENTEN pratar INTE SVENSKA TILLFREDSSTÄLLANDE***

*[Observera: om du kommit I Kontakt med någon som pratar ett främmande spark och vars svenska uppenbart inte är tillräcklig för att genomföra intervjun), säg:*

***Jag är ledsen men jag pratar bara svenska****.* ***Tack för att jag fick störa, adjö.***

1. ***PÅBÖRJANDET AV INTERVJUEN***

*[INTERVJUARENS MANUS]*

**Denna telefonundersökning är en del av en större undersökning i 8 europeiska länder. Målet är att mäta medborgarnas åsikter i frågan om hemlöshet och förstå deras förväntningar i den här frågan. Observera att under den här intervjun kommer bara din åsikt om situationen i Sverige att tas upp. Dina svar kommer att hållas strikt anonym. Jag kommer inte att be dig om ditt namn eller ditt förnamn. Om du inte vill svara på några frågor, kan du helt enkelt säga det.**

**Säger du ja till att delta i denna undersökning som mäter medborgarnas åsikter om hemlöshet?** JA

NEJ *(gå till sektion III VÄGRAN)*

*[Om ja]* **OK då startar vi!**

*[INTERVJUARENS MANUS]* **Jag börjar med några allmänna frågor om hemlösa. Först av allt, ska du veta att vi definierar hemlöshet som den om sover på gatan, i en bil, eller bor i en nödsituation eller tillfälligt skydd (eller med andrahandskontrakt tillhandahållet av socialtjänsten) .**

| *[OBS:* ***Läs aldrig upp alternativen*** *VI eller SI möjligheterna]* | VI= vet inte  SI= svarar inte |
| --- | --- |
|  |  |
| 1. **Så, har du någon gång varit hemlös?**   *[OBS :även en gång räknas som ja!]* | 1 = ja  2 = nej *(fortsätt till 4)*  3 = SI (*Fortsätt till 4*) |
| 1. *[Om ja till fråga 1]:***När var det?** *[läs upp alternativen]* | 1 = under de senast 12 månaderna  2 = 1-2 år sedan  3 = 3-4 år sedan  4 = 4-5 år sedan  5 = Mer än 5 år sedan  6 = VI  7 = SI |
| 1. *[Om ja till fråga till 1]:* **Hur länge totalt har du varit hemlös under ditt liv?** *[läs upp alternativen]* | 1 = mindre än 1 vecka  2 = mindre än en månad  3 = mindre än ett år  4 = mindre än två år  5 = mindre än fyra år  6 = Mer än 4 år  7 = VI  8= SI |
| 1. **Har någon av dina familjemedlemmar, vänner eller bekantar någon gång varit hemlösa?** | 1= Ja  2= Nej *(fortsätt till 6)*  3 = SI (*fortsätt till 6*)  4 = VI (*fortsätt till 6*) |
| 1. *[Om familj eller vänner någon gång varit hemlös =ja]* **Var det …** | 1 = Förälder *[far, mor]*  2 = Barn  3 = Broder/syster  4 = Man/fru/ partner  5 = Vänner  6 = Andra släktingar  7 = Bekanta  8 = VI  9 = SI |
| 1. **Vet du hur många människor som uppskattas vara hemlösa i Sverige?**   *[respondenten ska ge en siffra, inte procent]* | /_______________/ |
| 1. **Hur manga av de hemlösa, utifrån vad du tror, är det som ...**   *[respondenten ska svara i procent]* |  |
| 1. Har psykiska störningar? | _______% |
| 1. Har beroendeproblematik (alkohol, droger)? | _______% |
| 1. ***V*em finansierar största delen av den sociala insatser för hemlösa***?*   *[Endast ett svarsalternativ]* | 1 = Staten  2 = Frivillighetsorganisationer  3 = Kyrkor och religiösa föreningar  4 = VI  5 = SI |
|  |  |
| 1. **Vem finansierar största delen av vårdomsorgen för hemlösa?**   *[Endast ett svarsalternativ]* | 1 = Staten  2 = Frivillighetsorganisationer  3 = Kyrkor och religiösa föreningar  4 = VI  5 = SI |
| 1. **I ditt område där du bor, skulle du saga att det är många, några, få eller inga hemlösa?**   *[I ditt grannskap]* | 1 = Många  2 = Några  3 = Få  4 = Inga (*fortsätt till fråga 12)*  5 = VI  6 = SI |
| 1. **På ett ungefär, hur många olika hemlösa ser du på en vecka?**   *(om respondenten ser samma hemlös person fler gånger per vecka räknas det som en person]* | 1 = Inga  2 = 1 till 2  3 = 3 till 10  4 = mer än10  5 = VI  6 = SI |
| 1. **Borde offentliga myndigheter se på hemlöshet som ett prioriterat område?** | 1 = Ja  2 = Nej  3 = VI  4 = SI |
| 1. **När du passerar en hemlös person är du då på din vakt?** | 1= Ofta  2= Ibland  3= Sällan  4= Aldrig  5= VI  6 =SI |
| 1. **Under de senaste åren, har du…..?**   *[läs upp alternativen]* | 1 = Ja  2 = Nej  3 = VI  4 = SI |
| 1. Gett pengar, mat eller kläder till en hemlös | 1 2 3 4 |
| 1. Gett pengar, mat eller kläder till en hjälporganisation för hemlösa | 1 2 3 4 |
| 1. Arbetat som volontär för en hjälporganisation som jobbar för hemlösa. | 1 2 3 4 |
| 1. Annat*[specifiera]*: ___________________ | 1 2 3 4 |
| 1. **För att minska hemlösheten, skulle du vara beredd att** *läs upp alternativen]***?** | 1 = Ja  2 = Nej  3 = VI  4 = SI |
| 1. Betala mer skatt? | 1 2 3 4 |
| 1. Volontärarbeta? | 1 2 3 4 |
| 1. Acceptera ett hemlöshetshärbärge I ditt grannskap? | 1 2 3 4 |

| *(INTERVJUARENS MANUS*)  Nu vill vi veta vad du tycker om olika insatser för att hysa hemlösa.  Vet du att i Sverige:  uppskattas cirka 34 000 människor vara hemlösa; Mellan 25 – 50 % av dem har missbruksproblem och cirka 35 % har psykiska problem  I dagsläget är de vanligaste insatserna för hemlösa olika typer av boendeinsatser, från härbärgen till tillfälliga boenden via socialtjänsten.  En hemlös person kan stanna i upp till en vecka på härbärgen där man ofta sover i sovsalar. Efter flera månader av väntan, kan han/hon få tillgång till olika former av mer långsiktiga boendeinsatser. Beroende på hur man sköter sig kan man stanna där tre till sex månader om uppsatta regler efterlevs, som t.ex. att ta tag i sina övriga problem som missbruk och psykiska problem. Socialarbetare finns oftast tillgängliga. Par och personer med husdjur är inte tillåtna. Den vanligaste inkomstkällan är ekonomiskt bidrag från socialtjänsten.  I följande del presenteras ett innovativt nytt program för hemlösa personer.  Syftet är att ta reda på hur mycket du skulle vara beredd att betala för ett sådant program. Det är inte meningen att du ska uppskatta kostnaden för att genomföra ett sådant program utan bara saga något om det vore värdefullt eller ej, helt hypotetiskt och endast för forskningen skull. Det kommer inte att resultera i några skattehöjningar!  Du kan vara villig att betala mycket, lite eller ingenting alls för att genomföra ett sådant förändrat arbetssätt med hemlöshetsproblematiken.  Det finns inget rätt eller fel svar. | | |
| --- | --- | --- |
| **Detta innovative nya arbetssätt/program kallas *Housing First*/Bostad Först.**  Det riktar sig till hemlösa med psykiska eller fysiska hälsoproblem (alltså, psykiska sjukdomar, missbruksproblem eller funktionshinder).  För att kvalificera sig för denna typ av program behöver inte den hemlöse genomgå någon behandling för sina psykiska eller missbruksproblem.  De får individuella boendelösningar och ekonomiska bidrag utifrån behov.  Meningen är att de om möjligt ska betala sin hyra men om inkomst saknas kan de få försörjningsstöd.  Liknande program testas i ett antal kommuner i Sverige för några år tillbaka och har testats i andra europeiska länder som Frankrike och Nederländerna och har visat att majoriteten av de tidigare hemlösa kvarstår i huset efter två år. Samma sak har visats i de kommuner i Sverige där programmet testas. | | |
| 1. **Jag kommer att föreslå en summa i kronor. Var snäll och tala om för mig hur mycket du skulle acceptera att betala via skatt per år för ett sådant program.**   *[Intervjuare: Om det spontana svaret är ”noll kronor” eller vill inte betala, rapporterar 1 =JA här.*  *Gå sedan till 16.2]*  *[Intervjuare: efter det första SI eller “VI”* ***stoppa*** *och fortsätt med nästa fråga 16.1].*  **Skulle du vara villig att betala ………………………**  *[om respondenten sager det högsta budet (4000:-), gå vidare till fråga16.1].* | 1 = JA  2 = NEJ  3= VI  4 =SI  250:-? …….1 2 3 4 *[21:-/mån]*  500:-? …....1 2 3 4 *[42:-/mån]*  1000:-? …...1 2 3 4 *[84:-/mån]*  2000:-? …...1 2 3 4 *[160:-/mån]*  4000:-? …...1 2 3 4 *[325:-/mån]* |  |
| 1. **Vad skulle kunna vara det mesta du skulle kunna tänka dig att betala genom skattepåslag varje år för denna typ av program?**   *Påminn respondenten om den senaste summan de kunde acceptera (samt den summa som de in kunde tänka säg att betala. Målet är att få respondenten att välja ett högsta värde mellan de två).*  *T.ex.: om Ja till 250:- och inte till 500:- fråga: Mellan 250 och 500:- vad skulle var den maximala summan…* | /-----------/ :- |  |
| 1. *[Om respondenten svarade 0 kronor)* **Skulle du kunna berätta vad som är huvudskälet till varför du inte är beredd att betala något….Det är pga.…..** | 1 = Programmet kommer inte att fungera  2 = Andra program är viktigare/har högre prioritet  3 = Jag vill inte betala mer skatt  *(gå till 18)*  4 = Jag har inte råd att betala mer skatt *(gå till 18)*  5 = Annat: -------------------------------  6= VI  7 =SI |  |
| 1. **Hitintills har jag pratat om ett program för hemlösa med psykiska eller missbruksproblem. Skulle du vara villig att betala (mer, samma eller mindre) på årsbasis genom skatt om programmet var tillgängligt för alla hemlösa.** | 1= Mer *(gå till 17.1)*  2= Samma *(gå till 18)*  3= Mindre *(gå till 17.1)*  4= VI  5 = SI |  |
| 1. **Specifisera det maximal belopp du skulle vara villig att betala på årsbasis genom skatt för programmet?** | /----------/ :- |  |
| 1. **Hur säker är du på ditt svar?** | 1= mycket säker  2= ganska säker  3= mer eller mindre säker  4= relativt osäker  5 = mycket osäker |  |
| 1. **För dataanalysen behöver vi veta om dina inkomster är beskattningsbara ?** | 1 = Ja  2= Nej  3= vet ej  4 =svarar inte |  |

*[INTERVJUARENS MANUS].* **Tack för dina svar. Nu skulle jag vilja fråga dig om hur livssituationen är för hemlösa i Sverige.**

| 1. **Under de senaste 3 åren, skulle du säga att antalet hemlösa har…** | 1 = Ökat kraftigt  2 = Ökat lite  3 = minskat lite  4 = Kraftigt minskat  5 = Oförändrat  6 = VI  7 = SI |
| --- | --- |
| 1. **Jag kommer nu läsa upp ett antal påståenden om hemlösa. (***Läs dem***). Berätta om du……** | 1 = Håller med  2 = Håller delvis med  3 = Håller delvis inte med  4 = Håller inte alls med  5= VI  6 =SI |
| 1. Hemlösa människor är offer för övergrepp (våld, rån, hot och attacker). | 1 2 3 4 5 6 |
| 1. De diskrimineras vid anställningar | 1 2 3 4 5 6 |
| 1. De äter minst två mål mat om dagen. | 1 2 3 4 5 6 |
| 1. De har möjligheten att hålla Kontakt med släktingar och vänner. | 1 2 3 4 5 6 |
| 1. De har en kortare livslängd än den allmänna befolkningen | 1 2 3 4 5 6 |
| 1. Många är hemlösa av eget val. | 1 2 3 4 5 6 |
| 1. De flesta är kvalificerade för arbete | 1 2 3 4 5 6 |
| 1. De skulle kunna sköta ett hem om de hade ett. | 1 2 3 4 5 6 |
| 1. De har tillgång till betalt eller obetalt arbete (volontärjobb, etc.). | 1 2 3 4 5 6 |
| 1. Deras huvudsakliga inkomstkälla är försörjningsstöd. | 1 2 3 4 5 6 |
| 1. De spenderar mesta delen av sin tid utanför något socialt nätverk. | 1 2 3 4 5 6 |
| 1. **Utifrån din åsikt, den service som finns tillgänglig ……motsvarar behov hemlösa har:** | 1 = Håller med  2 = Håller delvis med  3 = Håller delvis inte med  4 = Håller inte alls med  5= VI  6 =SI |
| 1. På sjukhus och akuten | 1 2 3 4 5 6 |
| 1. På vårdcentraler och i öppenvård | 1 2 3 4 5 6 |
| 1. På härbärgen (max 7 dygn) | 1 2 3 4 5 6 |
| 1. Inom tillfälliga boendeinsatser (max 3 – 6 månader) | 1 2 3 4 5 6 |
| 1. Inom Bostad Först programmen tillgodoser man bättre hemlösas behov i jämförelse med tillfälliga boendeinsatser? | 1 2 3 4 5 6 |
| 1. **Enligt din åsikt, vilka är de tre huvudskälen till att människor blir hemlösa?**   *[OBS: vänta på spontana svar och pricka sedan av dem].* | 1 = förlorat arbetet/arbetslös  2 = för dålig inkomst/har inte råd att betala hyran  3 = pga. naturkatastrof  4 = överskuldsatt  5 = sjuk eller funktionshindrad  6 = missbruk/beroende  7 = förändrad familjesituation/skillsmässa/dödsfall  8 = psykiska problem  9 = ingen tillgång till försörjningsstöd eller social service  10 = illegal invandring  11 = eget val  12 = annat 1:___________________________________  13 = annat 2:___________________________________  14 = annat 3:___________________________________  15 = inget *(SPONTANT)*  16= VI  17 =SI |
| 1. **Enligt din åsikt, vem bör ha huvudansvaret för att tillgodose att det finns härbärgen för hemlösa?** | 1 = Staten  2 = Icke-statliga organisationer/NGO:s  3 = Kyrkor och religiösa föreningar  4 = De hemlösa själva  5= VI  6 = SI |
| 1. **Enligt din åsikt, vem bör ha huvudansvaret för att se till att det finns långsiktiga boendelösningar för hemlösa?** | 1 = Staten  2 = Icke-statliga organisationer/NGO:s  3 = Kyrkor och religiösa föreningar  4 = De hemlösa själva  5= VI  6 = SI |
| 1. **Vilken/a hemlösa grupper ska prioriteras för en långsiktig boendelösning?**   *[Öppen fråga, läs inte upp de olika förslagen utan pricka av de mest närliggande]* | 1 = Familjer  2 = Kvinnor  3 = Unga [<30 år utan barn]  4 = Människor med psykisk sjukdom  5 = Anställda  6 = Äldre  7 = Människor med funktionshinder  8 = Arbetslösa  9 = Människor med missbruksproblem  10 = Ingen  11 = Andra ___________________________________  12=VI  13=SI |
| 1. **Generellt, tycker du att staten lägger för mycket, lagom eller för lite på social välfärd…..** | 1 = För mycket  2 = Lagom  3 = För lite  4 = VI  5 = SI |
| 1. **Generellt, tycker du att staten lägger för mycket, lagom eller för lite på att hjälpa hemlösa?** | 1 = För mycket  2 = Lagom  3 = För lite (*gå till 30*)  4 = VI  5 = SI (*gå till 30*) |
| 1. **Kan ni förtydliga lite?**   *[OBS: vänta på spontana svar och pricka sedan av)*  **Är det för att…** | 1 = Hemlöshetsprogram är inte ett prioriterat område  2 = Det är bortkastade pengar då det är innefektivt  3 = Bortkastade pengar pga. hemlösa inte förtjänar det  4 = Regeringen satsar en lämplig summa pengar för att hjälpa hemlösa  5= Andra ___________________________________  6=VI  7 = SI |

***[INTERVjuarens MANUS]* Sociodemografiska bakgrundsinformation.**

**Slutligen vill jag ställa några sociodemografiska bakgrundsfrågor.**

| 1. *[För intervjuaren ENDAST, fråga inte respondenten!]* | 1= man  2=kvinna |
| --- | --- |
| 1. **Vilket år är du född?**   *[Koda med* ***4 siffror****. T.ex.: 1953 [Om det är någon tvekan fortsätt till nästa fråga)* | År: /---/---/---/---/ |
| - 1. Hur gammal är du? | Ålder: /----------/ år |
| 1. **Vilken nationallitet har du?** *[Land]* | /_______________________/ |
| 1. **Vilken är din högsta utbildning?**   *(Interviewer: Spontana svar förväntas, läs inte upp svaren…)* | 1 = Eftergymnasial utbildning (doktorerat, eller liknande, upp till 4 års eftergymnasial utbildning eller högre)  2 = Kandidatexamen eller Högskoleutbildning motsvarande upp till 3 års eftergymnasial utbildning  3 = Eftergymnasial utbildning (upp till 2 års eftergymnasial utbildning)  4 = Gymnasiet  5 = Högstadiet  6 = mellanstadiet  7 = lågstadiet  8 = har inte gått I skolan  9 = Andra kvalifikationer/utländsk examen  10= DK (Vet inte)  11= NA (Svarar inte) |
| 1. **För tillfället, hur ser din arbetssituation ut?**   *[Endast ett svarsalternativ]* | 1 = Arbetar heltid (35 timmar eller mer i veckan)  2 = Arbetar deltid (8 till 35 timmar i veckan)  3 = Arbetar deltid då och då (mindre än 8 timmar)  4 = Heltidsstuderande  5 = Pensionär  6 = Arbetslös  7 = Arbetar inte av andra skäl  8 = DK (Vet inte)  9 = NA (Svarar inte) |
| 1. **Vad är ditt hushålls bruttoinkomst/år?**   *[Intervjuare: vänta på ett spontant svar och pricka sedan av lämpligt alternativ]*  *[Intervjuare: om respondenten frågar efter mer information, specificera:]* **Du ska inkludera alla inkomstkällor.** | /----------------------------------------/kronor  1= ‘mindre än 50 000:-  2= mellan 50 000 & 100 000:-  3= mellan 100 000 & 150 000:-  4= mellan 150 000 & 200 000:-  5= mellan 200 000 & 300 000:-  6= mellan 300 000 & 400 000:-  7= mellan 400 000 & 500 000:-  8= mellan 500 000 & 600 000:-  9= mellan 600 000 & 700 000:-  10= mer än 700 000 :-  11 = VI  12 = SI |
| 1. **Vad är ditt civilstånd? Är du?** | 1 = gift  2 = änka/änkling  3 = separerad/skild  4 = Sambo  5 = ensamstående |
| 1. **Inklusive dig, hur manga bor I ditt hushåll?** | /-------------------/ |
| 1. **Hur många barn har du?** | /------------------/ |
| 1. **Hur många beroende barn (kopplat till hushållets taxerade inkomst) finns det?** | **/------------------/** |
| 1. **Vilket är ditt postnummer?**   *[Om respondenten inte vill svara på det fråga efter närmaste län/kommun, etc.]* | /-----------------/ |
| 1. Närmast större geografisk ort/län? |  |
| 1. **Skulle du säga att du bor… ?** | 1 = i ett storstadsområde  2 = i ett småstadsområde  3 = på landsbygden  4 = SI  5 = VI |
| 1. **Hur många fasta telefonnummer har hushållet?** | /------------------/ |
| 1. **Hur manga mobiltelefoner finns det I hushållet**? | /------------------/ |
| 1. Har du en mobiltelefon? | 1 = Ja  2 = Nej  3 = SI |

**NU ÄR INTERVJUN KLAR**

**Karolinska institutet tackar för ditt deltagande.**

**Har du några frågor?** *[Om ja, försök verkligen att besvara dem].*

*[Om du inte kan besvara dem]* ***Jag är ledsen men jag har inte den informationen. Men det du kan göra är att skicka ett mail till:*** mats.blid@ki.se

*[instruktion till intervjuaren: Var snäll och notera frågan inför nästa träningssession.]*

*Frågan var: ------------------------------------------------------------------------------------------------------------------------------------------------------------------------------------------------------------------------------------------------------------------*

**Och om det skulle vara så att det dyker upp någon fråga eller kommentar senare kan du ringa projektledaren (Håkan Källmén),** **på KI**.

**Telefonnumret är 0703683037**

**A kortfattad rapport om resultaten av undersökningen kommer att finnas tillgänglig på följande adress:**

**home-eu.org**

**Kom ihåg, ditt deltagande är anonymt och ännu en gång, tack för ditt deltagande.**

| 1. *FRÅGOR OM INTERVJUN: Svarade respondenten på de flesta frågorna på ett tillförlitligt sätt?*   *Var det några problem som skulle göra att inhämtad information inte är pålitlige?* | 1 = INGA PROBLEM  2 = EVENTUELLA PROBLEM (beskriv nedan): _________________________________________________  _________________________________________________  3 = ALLVARLIGA PROBLEM (beskriv nedan): _________________________________________________  _________________________________________________ |  |
| --- | --- | --- |

| ANKIETA  OPINIA PUBLICZNA NA TEMAT BEZDOMNOŚCI I PREFERENCJI |
| --- |
| NUMER IDENTYFIKACYJNY: /__/__/__/__/__/  DATA: (____/____/____)  CZAS ROZPOCZĘCIA ANKIETY : (____:____) CZAS ZAKOŃCZENIA: (____:____)  PROWADZĄCY : _________________________ KOD PROWADZĄCEGO : __________ |

1. ***WSTĘP***

*[SCENARIUSZ DLA PROWADZĄCEGO]*

**Witam, nazywam się ----------------------------------. Dzien dobry/dobry wieczor, nazywam sie .... i prowadze ankiete dla Unii Europejskiej, wraz z Uniwersytetem z Marsylii na temat PROBLEMU BEZDOMNOSCI w Polsce. Rozmowa jest na moj koszt. Czy ma Pan/Pani troche czasu aby odpowiedziec na kilka pytan?**

**Czy jest Pan/Pani pełnoletni/a?**

*[Jeśli tak , przejdź do części II MOŻLIWOŚĆ WZIĘCIA UDZIAŁU W ANKIECIE]*

*[Jeśli nie, zapytaj]:* **Czy mogę rozmawiać z kimś pełnoletnim znajdującym się teraz w domu?**

1. *[Jeśli tak, kiedy osoba podejdzie do telefonu, kontynuuj]:* **Dzien dobry/dobry wieczor, nazywam sie .... i prowadze ankiete dla Unii Europejskiej, wraz z Uniwersytetem z Marsylii na temat PROBLEMU BEZDOMNOSCI w Polsce. Rozmowa jest na moj koszt. Czy ma Pan/Pani troche czasu aby odpowiedziec na kilka pytan?**

*[następnie przejdź do części II MOŻLIWOŚĆ WZIĘCIA UDZIAŁU W ANKIECIE]*

1. *[Jeśli nie, osoba pełnoletnia jest nieobecna:]* **Kiedy i w jakich godzinach będzie można porozmawiać z osobą pełnoletnią?**

[ZAPISZ ABY ODDZWONIĆ]: Data: _______ Godzina: ________IMIĘ (tylko pierwsze): ________ **Dziękuję, oddzwonię w podanym przez Pana/Panią terminie.**

1. [Jeśli nie wiadomo czy można lub nie:]: **Dziękuję, spróbuję skontaktować się z Państwem ponownie w innym terminie;**
2. ***Możliwość wzięcia udziału w ankiecie***

**Czy mogę Panu/Pani zająć około 20 minut?**

1. [*Jeśli tak]:* **Dziękuję!** *(przejdź do sekcji V ROZPOCZĘCIE BADANIA)*
2. *[Jeśli nie, zapytaj*:] **Kiedy możemy zadzwonić ponownie w odpowiednim dla Pana/Pani czasie?**

[*ZAPISZ ABY ODDZWONIĆ*:] Data:__________ Godzina: _______________ **Dziękuję, odzwonie do Pana/Pani w podanym terminie.**

- - *[Jeśli nie lub nie wie, powiedz*:]  **Pana/Pani opinia na ten temat jest dla nas bardzo istotna. Jest Pan/Pani pewien/pewna, że nie możemy porozmawiać?**
    - *[Jeśli odpowiedź brzmi tak,możemy].* **Dziękuję!** *(przejdź do sekcji V ROZPOCZĘCIE BADANIA]*
    - [*Jeśli odpowiedź brzmi tak ale w innym terminie, zapisz datę i godzinę]*

Data:_______________ Godzina: _______________ **Dziękuję, oddzwonie do Pana/Pani.**

- - - *[Jeśli nie, przejdź do sekcji III ODMOWA].*

1. ***ODMOWA***
   1. **Rozumiem, że nie chce Pan/Pani brać udziału w badaniu; ale czy mogłby Pan/mogłaby Pani podać swój wiek, wykształcenie i status zatrudnienia abyśmy mogli przeprowadzić analizę odmowy w badaniu?**

1 = Tak

2 = Nie

- 1. **Data urodzenia: ____________________**
  2. **Najwyższy poziom wykształcenia:**

1 = Wyższe (magister, doktor, itp../ przynajmniej 4 lata studiów wyższych)

2 = Licencjackie lub inżynierskie (przynajmniej 3 lata studiów wyższych)

3 = Pomaturalne (powyżej 2 lat edukacji po maturze)

4 = Średnie (maturalne, liceum i technikum)

5 = Zawodowe

6 = Gimnazjalne

7 = Podstawowe

8 = Brak wykształcenia

9 = Inne kwalifikacje (kursy, szkolenia)

10 = Nie wiem

11 = Brak odpowiedzi

- 1. **Status zatrudnienia :**

1 = Praca na pełen etat (40 godzin tygodniowo lub więcej)

2 = Praca na część etatu (8 do 35 godzin tygodniowo)

3 = Praca dorywcza

4 = Głównie studiuję

5 = Emeryt

6 = Bezrobotny

7 = Nie pracujący z innych powodów

8 = Nie wiem

9 = Brak odpowiedzi

- 1. **Ile osób mieszka w Pana/Pani gospodarstwie domowym wliczając Pana/Panią? ____**
  2. **Ile dzieci poniżej 18 roku życia mieszka w Pana/Pani gospodarstwie domowym?______**

**Dziękuję za pomoc i przepraszam za kłopot.**

1. ***Gdy rozmówca nie odpowiada zrozumiale***

*[Pamiętaj: jeśli osoba mówi w obcym języku ( lub język którym się posługuje) jest niezrozumiały dla Prowadzącego aby przeprowadzić badanie), powiedz:****]***

***Przepraszam, mówię tylko w języku polskim****.* ***Dziękuję za poświęcony czas. Do widzenia.***

1. ***ROZPOCZĘCIE BADANIA***

*[SCENARIUSZ DLA PROWADZĄCEGO]*

**Ankieta ta jest częścią badania przeprowadzanego w 8 europejskich krajach. Jej celem jest sprawdzenie opinii obywateli na temat problemu bezdomności oraz zrozumienie ich oczekiwań dotyczących rozwiązania tego problemu. W ankiecie tej, będziemy brać pod uwagę Pana/Pani opinię wyłącznie na temat bezdomności w Polsce. Pana/Pani odpowiedzi, jak i tożsamość pozostaną anonimowe. Jeżeli nie życzy sobie Pan/Pani odpowiadać na niektóre pytania, możemy je pominąć.**

**Czy zgadza się Pan/Pani na wzięcie udziału w sondażu służącym badaniu opinii obywateli na temat bezdomności?** TAK

NIE *(przejdź do sekcji III ODMOWA)*

*[Jesli tak]***Dobrze, więc możemy zaczynać.**

*[SCENARIUSZ DLA PROWADZĄCEGO]***Rozpocznę od zadania Panu/Pani ogólnych pytań dotyczących bezdomności. Przypomnę Panu/Pani że bezdomność definiujemy poprzez spanie na ulicy, w samochodzie lub przez przebywanie w schroniskach lub ośrodkach pomocy dla bezdomnych.**

| *[Pamiętaj :* ***Nigdy*** *nie odczytuj na głos opcji odpowiedzi NW lub OO]* | NW= Nie wiem  OO = Odmawiam odpowiedzi |
| --- | --- |
|  |  |
| 1. **Czy był/a Pan/Pani kiedykolwiek bezdomna?**   *[Pamiętaj: nawet jeśli tylko przez jedną noc, liczy się jako TAK]* | 1 = TAK  2 = NIE *(przejdź do pyt.4)*  3 = OO (*przejdź do pyt.4*) |
| 1. *[Jeśli odpowiedź na pyt.1 brzmi TAK]:* **Kiedy taka sytuacja miała miejsce?** *[przeczytaj opcje odpowiedzi]* | 1 = W ciągu ostatnich 12 miesięcy  2 = 1-2 lata temu  3 = 3-4 lata temu  4 = 4-5 lata temu  5 = Więcej niż 5 lat temu  6 = NW  7 = OO |
| 1. *[Jeśli odpowiedź na pyt.1 brzmi TAK]:***Przez jak długi okres czasu był/a Pan/Pani bezdomna?***[przeczytaj opcje odpowiedzi]* | 1 = Mniej niż tydzień  2 = Mniej niż miesiąc  3 = Mniej niż rok  4 = Mniej niż dwa lata  5 = Mniej niż cztery lata  6 = Więcej niż pięć lat  7 = NW  8= OO |
| 1. **Czy ktoś z Pana/Pani rodziny, przyjaciół lub znajomych był kiedykolwiek bezdomny?** | 1 = TAK  2 = NIE *(Przejdź do pyt.6)*  3 = NW (*Przejdź do pyt. 6)*  4 = OO (*Przedź do pyt. 6)* |
| 1. *[Jeśli ktoś z rodziny lub znajomych był bezdomny = TAK]* **Czy był/była to …** | 1 = Rodzic*[ojciec, matka]*  2 = Dziecko  3 = Brat/siostra  4 = Mąż/Żona/Partner  5 = Przyjaciel  6 = Krewny  7 = Znajomy  8 = NW  9 = OO |
| 1. **Czy potrafi Pan/Pani podać w przybliżeniu liczbę bezdomnych w Polsce?**   *[rozmówca powinien podać konkretną liczbę a nie wartość procentową]* | /_______________/ |
| 1. **W Pana/Pani opinii, jaki jest procent bezdomnych z…**   *[rozmówca powinien podać wartość procentową]* |  |
| 1. Zaburzeniami psychicznymi? | _______% |
| 1. Problemem uzależnienia (narkotyki, alkohol)? | _______% |
| 1. **Z jakich środków w większości są finansowane ośrodki pomocowe dla osób bezdomnych w Polsce***?*   *[Oczekuje się tylko jednej odpowiedzi]* | 1 = Rządowych  2 = Organizacji Pozarządowych/ Fundacji  3 = Organizacji kościelnych i wspólnot religijnych  4 = NW  5 = OO |
| 1. **Z jakich środków w większości finansowana jest opieka zdrowotna dla osób bezdomnych?**   *[Oczekuje się tylko jednej odpowiedzi]* | 1 = Rządowych  2 = Organizacji Pozarządowych/ Fundacji  3 = Organizacji kościelnych i wspólnot religijnych  4 = NW  5 = OO |
| 1. **Czy według Pana/Pani opinii, w okolicy w której Pan/Pani mieszka, spotyka się wielu, kilku bądź nie spotyka się w ogóle osób bezdomnych?**   *[W okolicy w kórej Pan/Pani mieszka= w Pana/Pani sąsiedztwie]* | 1 = Wiele osób  2 = Kilka osób  3 = Mało osób  4 = Żadnych (*Przejdź do pyt.12)*  5 = NW  6 = OO |
| 1. **Ile różnych osób bezdomnych spotyka Pan/Pani średnio w ciągu tygodnia?**   *[Jeśli rozmówca spotyka tę samą osobę kilka razy w tygodniu, liczymy ją jako 1 osobę]* | 1 = Żadnej  2 = 1 lub 2  3 = od 3 do 10  4 = Więcej niż 10 osób  5 = NW  6 = OO |
| 1. **Czy polskie władze powinny traktować bezdomność jako priorytet?** | 1 = TAK  2 = NIE  3 = NW  4 = OO |
| 1. **Czy mijając na ulicy osobę bezdomną zachowuje Pan/Pani ostrożność?** | 1= Często  2= Czasami  3= Rzadko  4= Nigdy  5= NW  6 =OO |
| 1. **Czy w ciągu ostatniego roku…**   *[przeczytaj opcje odpowiedzi]* | 1 = TAK  2 = NIE  3 = NW  4 = OO |
| 1. Podarował/a Pan/Pani pieniądze, żywność lub ubrania osobie bezdomnej | 1 2 3 4 |
| 1. Podarował/a Pan/Pani pieniądze, żywność lub ubrania organizacji non-profit bądź fundacji pomagającej osobom bezdomnym | 1 2 3 4 |
| 1. .Pracował/a Pan/Pani jako wolontariusz/wolontariuszka dla organizacji non-profit bądź fundacji wspierającej osoby bezdomne | 1 2 3 4 |
| 1. Udzielił/a Pan/Pani wsparcia w inny sposób*[poproś o sprecyzowanie]*: ___________________ | 12 3 4 |
| 1. **Czy aby zmniejszyć problem bezdomności wyraziłby/ wyraziłaby Pan/Pani chęć***[przeczytaj opcje odpowiedzi]***?** | 1 = TAK  2 = NIE  3 = NW  4 = OO |
| 1. Płacenia wyższych podatków? | 1 2 3 4 |
| 1. Brania udziału w akcjach charytatywnych? | 1 2 3 4 |
| 1. Umieszczenia w Pana/Pani sąsiedztwie schroniska dla osób bezdomnych? | 1 2 3 4 |

**Moduł *Wyrażenie chęci wsparcia finansowego***

*[Pamiętaj:* ***Nigdy*** *nie odczytuj na głos opcji NW lub OO]*

| [Scenariusz dla prowadzącego]  Teraz chcielibyśmy poznać Pana/Pani opinię na temat różnych sposobów zapobiegania bezdomności. Czy wie Pan/Pani, że w Polsce:  36 200 Polaków jest bezdomnych (w tym osoby, które śpią na ulicy oraz mieszkańcy ośrodków dla osób bezdomnych); Ponad 30% z nich cierpi z powodu uzależnień (od alkoholu lub narkotyków) a ponad 12% z powodu złego stanu zdrowia oraz różnych zaburzeń psychicznych?  Obecnie, rozwiązaniem dla osób bezdomnych jest umieszczenie ich w awaryjnych noclegowniach i tymczasowym zakwaterowaniu dla osób bezdomnych (takich jak hostel, dom dla bezdomnych). Schroniska dostarczają zróżnicowanych warunków mieszkaniowych przez wiele miesięcy; Mieszkańcy schronisk muszą dzielić z innymi piętrowe łóżko, szafę, stół i umywalkę. Przestrzeganie trzeźwości oraz brak problemów ze zdrowiem psychicznym są powszechnie stawianymi warunkami korzystania ze schroniska. Osoby bezdomne mogą być kierowane do tymczasowego zakwaterowania na wniosek komisji selekcyjnej. Schroniska zatrudniają pracowników socjalnych, którzy wspierają bezdomnych, świadczą usługi doradcze w zakresie leczenia uzależnień lub pomagają rozwiązać inne problemy zgłaszane przez bezdomnych.  W kolejnej części chcielibyśmy zaprezentować innowacyjny program rozwiązywania problemu bezdomności.  Pana/Pani zadaniem będzie teraz zaproponowanie kwoty jaką Pan/Pani byłby/byłaby skłonny/skłonna wpłacić na ten program. Nie chcę, aby oszacował/a Pan/Pani rzeczywisty koszt programu, ale powiedział/a jaką przydatność miałby ten program dla rozwiązania problemu bezdomności. Pana/Pani odpowiedź będzie czysto hipotetyczna i użyta tylko w celach badawczych. Pana/Pani opinia nie wpłynie na podniesienie podatku!  Może Pan/Pani wyrazić chęć wsparcia dużą kwotą, małą lub nie wyrażać chęci wsparcia.  W tej ankiecie nie ma złych ani dobrych odpowiedzi. | | |
| --- | --- | --- |
| **Ten innowacyjny program nosi nazwę “Najpierw mieszkanie” (*Housing first*).**  Jest on skierowany do osób bezdomnych dotkniętych problemami zdrowotnymi, psychicznymi lub fizycznymi (zaburzenia psychiczne, uzależnienie od alkoholu lub narkotyków, niepełnosprawność).  Aby wziąć udział w programie osoba bezdomna nie jest zobowiazana do podjecia leczenia.  Osoba bezdomna objęta programem ma indywidualne mieszkanie i zapewnioną opiekę zdrowotną oraz społeczną dostosowaną do własnych potrzeb (do 7 dni w tygodniu).  Spłacają one część czynszu, ale w przypadku niewystarczających dochodów dostępne są dotacje.  Podobny program jest obecnie testowany w kilku krajach europejskich i pokazuje, że po dwóch latach zdecydowana większość osób bezdomnych (ponad 80%) włączonych do tego programu pozostaje w swoich mieszkaniach. | | |
| 1. **Zaproponuję Panu/Pani teraz kwoty . Proszę powiedzieć mi, ile byłby/byłaby Pan/Pani skłonny/a zapłacić każdego roku w ramach podatku na ten program.**   *[Dla Prowadzącego: jeśli odpowiedź brzmi “zero”lub rozmówca nie chce płacić,zaznacz 1 = TAK .*  *Następnie przejdź do pytania 16.2]*  *[Dla Prowadzącego: po pierwszej ODMOWIE lub “NIE WIEM” przedź do pytania 16.1].*  **Czy byłby/aby Pan/Pani skłonny/a zapłacić.. ………………………**  *[Jeśli rozmówca akceptuje najwyższą kwotę przejdź do pytania 16.1].* | 1 = TAK  2 = NIE  3= NW  4 =OO  0zł == >1  *(Proszę ustalić stawki według kraju)*  40zł? …… 1 2 3 4 *[3 zł/mies.]*  100zł?…….1 2 3 4 *[8 zł/mie.]*  200zł? …....1 2 3 4 *[17 zł/ mies.]*  400zł? …...1 2 3 4 *[33 zł/mies.]*  800zł? …...1 2 3 4 *[67 zł/mies]*  1600zł? …...1 2 3 4 *[133 zł/mies]* |  |
| - 1. **Jaką najwyższą kwotę byłby Pan/Pani skłonny/a zapłacić rocznie na ten program?**   *Przypomnij rozmówcy ostatnią zaakceptowaną kwotę (oraz kwotę którą odrzucił ,jeśli to możliwe). Celem jest otrzymanie od rozmówcy informacji na temat kwoty pośredniej pomiędzy podanymi dwoma wartościami ]. Np:jeśli tak dla 100zł i nie dla 200zł, zapytaj: Jaką maksymalną kwotę pomiędzy 100zł a 200zł byłby/byłaby Pan/Pani zapłacić ...* | /-----------/ zł |  |
| *16.2.[Jeśli rozmówca odpowiedział 0zł]* **Czy może Pan/Pani podać powód dlaczego odmówiłby/odmówiłaby Pan/Pani? Czy to dlatego, że**… | 1 = Program nie zadziała  2 = Inne programy wsparcia są ważniejsze/bardziej potrzebne  3 = Nie chcę płącić wyższych podatków  *(przejdź do pytania 18)*  4 = Nie stać mnie na płacenie wyższych podatków *(przejdź do pytania 18)*  5 = Inny powód: ---------------------------  6= NW  7 =OO |  |
| 1. **Na razie opowiedziałem/opowiedziałam Panu/Pani o programie na rzecz bezdomnych z zaburzeniami psychicznymi i nałogami. Czy byłby/byłaby Pan/Pani skłonny/a zapłacić (więcej, tyle samo, mniej) każdego roku w ramach podatku, jeśli ten program byłby skierowany do wszystkich bezdomnych?** | 1= Większą*(przejdź do pytania 17.1)*  2= Tą samą*(przejdź do pytania 18)*  3= Mniejszą*(przejdź do pytania 17.1)*  4= NW  5 = OO |  |
| 1. **Jaką maksymalną sumę byłby/aby skłonny Pan/Pani przeznaczyć każdego roku w ramach podatku na ten program?** | /----------/ zł |  |
| 1. **Czy jest Pan/Pani pewien/pewna swoich odpowiedzi?** | 1= zdecydowanie tak  2= raczej tak  3= trudno powiedzieć  4= raczej nie  5 = zdecydowanie nie |  |
| 1. **Czy Pana/Pani dochody są opodatkowane? Prosimy o odpowiedź tylko na potrzeby badania.** | 1 = TAK  2= NIE  3= NW  4 =OO |  |

*[SCENARIUSZ DLA PROWADZĄCEGO].***Dziękuję za odpowiedź. Teraz zadam Panu/Pani kilka pytań dotyczących warunków życia osób bezdomnych w Polsce***.*

| 1. **Czy według Pana/Pani liczba bezdomnych w ciągu ostatnich trzech lat …** | 1 = Znacznie się powiększyła  2 = Nieznacznie się powiększyła  3 = Nieznacznie zmalała  4 = Znacznie zmalała  5 = Niezmieniła się  6 = NW  7 = OO |
| --- | --- |
| 1. **Przeczytam Panu/Pani kilka opinii na temat osób bezdomnych** *[Przeczytaj opcje odpowiedzi]* **Proszę powiedzieć czy ….** | 1 = ZDECYDOWANIE SIĘ ZGADZA  2 = Raczej się ZGADZA  3 = Raczej się NIE ZGADZA  4 = ZDECYDOWANIE SIĘ NIE ZGADZA  5= NW  6 = OO |
| 1. Osoby bezdomne są ofiarami prześladowań (takich jak agresji, kradzieży, gróźb i ataków). | 1 2 3 4 5 6 |
| 1. Są dyskryminowane przy zatrudnieniu | 1 2 3 4 5 6 |
| 1. Jedzą przynajmniej dwa posiłki dziennie. | 1 2 3 4 5 6 |
| 1. Są w stanie kontaktować się z rodziną i przyjaciółmi. | 1 2 3 4 5 6 |
| 1. Mają mniejsze oczekiwania co do długości swojego życia niż ogół społeczeństwa. | 1 2 3 4 5 6 |
| 1. Wielu z nich jest bezdomnymi z wyboru | 1 2 3 4 5 6 |
| 1. Większość z nich posiada umiejętności zawodowe | 1 2 3 4 5 6 |
| 1. Dbaliby o dom (o czystość, urządzenie) gdyby go mieli | 1 2 3 4 5 6 |
| 1. Mają możliwość podjęcia płatnej lub bezpłatnej pracy (takiej jak wolontariat, praktyki itp.). | 1 2 3 4 5 6 |
| 1. Ich głównym źródłem dochodu są zasiłki socjalne | 1 2 3 4 5 6 |
| 1. Większość czasu spędzają samotnie, z dala od społeczeństwa | 1 2 3 4 5 6 |
| 1. **Według Pana/Pani czy usługi świadczone przez ….. zaspokajają w pełni potrzeby osób bezdomnych?** | 1 = ZDECYDOWANIE SIĘ ZGADZA  2 = Raczej się ZGADZA  3 = Raczej się NIE ZGADZA  4 = ZDECYDOWANIE SIĘ NIE ZGADZA  5= NW  6 = OO |
| 1. przez szpitale i pogotowia ratunkowe | 1 2 3 4 5 6 |
| 1. przez lekarzy ogólnych i specjalistów | 1 2 3 4 5 6 |
| 1. przez noclegownie   *[Dla Prowadzącego : maksymalna długość pobytu ; 7 dni]* | 1 2 3 4 5 6 |
| 1. W schroniskach   *[Dla Prowadzącego; maksymalna długość pobytu 3-6 miesięcy]* | 1 2 3 4 5 6 |
| 1. Czy program Najpierw mieszkanie lepiej spełnia potrzeby osób bezdomnych w porównaniu z noclegowniami i schroniskami? | 1 2 3 4 5 6 |
| 1. **Według Pana/Pani jakie są TRZY główne powody bezdomności ?**   *[Pamiętaj: poczekaj na odpowiedź i zaznacz tą zbliżoną do odpowiedzi rozmówcy ].* | 1 = utrata pracy/ długi okres poszukiwania zatrudnienia  2 = niskie dochody/brak możliowści opłacenia czynszu  3 = dom został zniszczony przez katastrofę naturalną (pożar, powódź, itp.)  4 = zadłużenie  5 = choroba lub niepełnosprawność  6 = uzależnienie (od alkoholu, narkotyków lub innych używek)  7 = rozstanie, rozwód, utrata bliskiej osoby  8 = zaburzenia psychiczne  9 = brak dostępu do zasiłku społecznego lub pomocy społecznej  10 = nielegalna imigracja  11 = własny wybór  12 = Inny 1:___________________________________  13 = Inny 2:___________________________________  14 = Inny 3:___________________________________  15 = Żaden *(ODPOWIEDŹ ODRUCHOWA)*  16= NW  17 =OO |
| 1. **Według Pana/Pani, kto powinien być w szczególności odpowiedzialny za zapewnienie SCHRONIENIA AWARYJNEGO dla osób bezdomnych?** | 1 = Państwo  2 = Organizacje pozarządowe/Fundacje  3 = Organizacje kościelne i wpólnoty religijne  4 = Sami bezdomni  5= NW  6 = OO |
| 1. **Według Pana/Pani, kto powinien być w szczególności odpowiedzialny za zapewnienie MIESZKANIA DŁUGOTERMINOWEGO dla osób bezdomnych?** | 1 = Państwo  2 = Organizacje pozarządowe/Fundacje  3 = Organizacje kościelne i wspólnoty religijne  4 = Sami bezdomni  5 = NW  6 = OO |
| 1. **Która grupa bezdomnych powinna w pierwszej kolejności zostać objęta programem długoterminowego zakwaterowania?**   *[Pytanie otwarte, nie należy przedstawiać możliwych odpowiedzi*  *Poczekaj na odpowiedź i zaznacz tą najbardziej zbliżoną do rozmówcy lub gdy taka nie istnieje zaznacz "inna"]* | 1 = Rodziny  2 = Kobiety  3 = Młodzi [poniżej 30 roku życia, bezdzietni]  4 = Osoby z zaburzeniami psychicznymi  5 = Pracujący  6 = Seniorzy  7 = Osoby niepełnosprawne  8 = Bezrobotni  9 = Osoby z problemem uzależnienia od alkoholu lub narkotyków  10 = Żadna  11 = Inna___________________________________  12=NW  13=OO |
| 1. **Czy uważa Pan/Pani że Państwo przeznacza (zbyt dużo, wystarczająco dużo, za mało) środków na zasiłki socjalne?** | 1 = Za dużo  2 = Wystarczająco dużo  3 = Za mało  4 = NW  5 = OO |
| 1. **Czy uważa Pan/Pani, że Państwo przeznacza (za dużo, wystarczająco dużo, za mało) środków na pomoc bezdomnym?** | 1 = Za dużo  2 = Wystraczająco dużo  3 = Za mało *(przejdź do pytania 30)*  4 = NW  5 = OO *(przejdź do pytania 30)* |
| 1. **Czy mógłby/mogłaby Pan/Pani uściślić swoją odpowiedź?**   *[Pamiętaj: poczekaj na odpowiedź i zaznacz najbardziej zbliżoną]*  **Czy to dlatego że…** | 1 = Programy pomocy bezdomnym nie są priorytetem  2 = Jest to strata środków ponieważ polityka pomocy jest nieefektywna  3 = Jest to strata środków ponieważ osoby bezdomne na nie nie zasługują  4= Nasz rząd wydatkuje odpowiednią kwotę na pomoc bezdomnym  5= Inne  6 = NW  7 =OO |

***[scenariusz prowadzącego]* cechy społeczno-demograficzne.**

**Na koniec zadam Panu/Pani kilka pytań aby określić Pana/Pani profil społeczno-demograficzny.**

| 1. *[TYLKO dla prowadzącego nie pytaj rozmówcy!* | 1= mężczyzna  2= kobieta |
| --- | --- |
| 1. **Proszę podać datę urodzenia?**   *[Zapisz używając* ***4 cyfr*** *Na przykład: 1953 rok zapisujemy 1953, rok 90 zapisujemy 1990.*  *[Jeśli rozmówca się waha, przejdź do pytania o wiek]* | Rok: /---/---/---/---/ |
| - 1. Ile maPan/Pani lat? | Wiek: /----------/ lat |
| 1. **Jakiej jest Pan/Pani narodowości?***[zapisz kraj]* | /_______________________/ |
| 1. **Jakie posiada Pan/Pani wykształcenie?**   *Dla Prowadzącego: poczekaj na odpowiedź, nie przedstawiaj możliwych odpowiedzi…* | 1 = Wyższe (magister, doktor, itp../ przynajmniej 4 lata studiów wyższych)  2 = Licencjackie lub inżynierskie (przynajmniej 3 lata studiów wyższych)  3 = Pomaturalne (powyżej 2 lat edukacji po maturze)  4 = Średnie (maturalne, liceum i technikum)  5 = Zawodowe  6 = Gimnazjalne  7 = Podstawowe  8 = Brak wykształcenia  9 = Inne kwalifikacje (kursy,szkolenia)  10 = Nie wiem  11= Brak odpowiedzi |
| 1. **Jaki jest Pana/Pani obecny status zatrudnienia?**   *[Oczekuje się tylko jednej odpowiedzi]* | 1 = Praca na pełen etat (35 godzin tygodniowo lub więcej)  2 = Praca na część etatu (8 do 35 godzin tygodniowo)  3 = Praca dorywcza  4 = Głównie studiuję  5 = Emeryt  6 = Bezrobotny  7 = Nie pracujący z innych powodów  8 = Nie wiem  9 = Brak odpowiedzi |
| 1. **Jaki jest całościowy roczny przychód brutto wszystkich osób w Pana/Pani gospodarstwie domowym (wliczając podatek dochodowy, składki ZUS)?**   *[Prowadzący: poczekaj na odpowiedź i zaznacz odpowiednią, w przypadku odmowy podania konkretnego przychodu, zaproponuj kwotę]*  *[Prowadzący: jeśli rozmówca poprosi o dodatkowe informacje, sprecyzuj:]***Proszę wziąć pod uwagę wszystkie źródła dochodu takie jak: pensja, emerytura, zasiłki socjalne, lokaty, renta zdrowotna.** | /----------------------------------------/  *(Proszę o podanie kwoty według kraju)*  1= mniej niż 20000. zł  2= od 20000 do 40000 zł  3= od 40000 do 60000zł  4= od 60000 do 80000 zł  5= od 80000 do 120000 zł  6= od 120000 do 160000 zł  7= od 160000 do 200000 zł  8= od 200000 do 240000 zł  9= od 240000 do 280000 zł  10= więcej niż 280000 zł  11 = NW  12 = OO |
| 1. **Jaki jest Pana/Pani stan cywilny? Jest Pan/Pani...?** | 1 = żonaty/zamężna  2 = wdowcem/wdową  3 = w separacji/rozwiedziony/rozwiedziona  4 = w związku (niezalegalizowanym)  5 = wolny/wolna |
| 1. **Ile osób mieszka w Pana/Pani gospodarstwie domowym wliczając Pana/Panią?** | /-------------------/ |
| 1. **Ile ma Pan/Pani dzieci?** | /------------------/ |
| 1. **Ile ma Pan/Pani dzieci na utrzymaniu (jest ono wliczone w roczny przychód gospodarstwa)?** | **/------------------/** |
| 1. **Jaki jest Pana/Pani kod pocztowy?**   *[Jeśli rozmówca nie wie lub nie chce podać tej informacji, zapytaj o najbliższą jednostkę podziału administracyjnego taką jak np. gmina, powiat, województwo)* | /-----------------/ |
| 1. Jednostka podziału administracyjnego | /-----------------/ |
| 1. Czy możesz powiedzieć, że mieszkasz…… ? | 1 = na terenie miejskim  2 = na terenie podmiejskim  3 = na terenie wiejskim  4 = NW  5 = OO |
| 1. **Ile numerów telefonów stacjonarnych znajduje się w Pana/Pani gospodarstwie?** (wykluczając numery nie używane lub służbowe)**?** | /------------------/ |
| 1. **Ile osób w Pana/Pani gospodarstwie posiada numery komórkowe**  (wykluczając numery służbowe)? | /------------------/ |
| 1. Czy posiada Pan/Pani telefon komórkowy? | 1 = Tak  2 = Nie  3 = OO |

**ZAKOŃCZENIE BADANIA**

**To już koniec naszego badania!**

(wypełnić nazwą instytucji przeprowadzającej badanie) **dziękuje za Pana/Pani udział.**

**Czy ma Pan/Pani jakieś pytania?** *[Jeśli tak, prowadzący powinien starać się odpowiedzieć jak najdokładniej].*

*[Jeśli prowadzący nie potrafi odpowiedziećlub nie jest pewien]* ***Przykro mi ale nie mogę udzielić Panu/Pani informacji na ten temat. Proszę skontaktować się z nami poprzez adres e-mail (****podać adres e-mai do kontaktu):* [***HOME_EU@ispa.pt***](mailto:HOME_EU@ispa.pt)

*[Instrukcja dla prowadzącego: PROSZĘ zapisać pytanie, zostanie ono wykorzystane przy następnym szkoleniu*

*Pytania: ------------------------------------------------------------------------------------------------------------------------------------------------------------------------------------------------------------------------------------------------------------------*

**Jeśli ma Pan/Pani jeszcze jakieś pytania lub uwagi, proszę skontaktować się z kierownikiem projektu, Dr Anna BOKSZCZANIN**, **z** Instytut Psychologii, Uniwersytet Opolski , 45-052 Opole.

**Numer telefonu to:** . +48 77 4527370

**Krótki raport z wynikami badania będzie dostępny po jego zakończeniu na:** [**www.HOME-EU.ORG**](http://www.HOME-EU.ORG).

**Przypominam, że ankieta jest anonimowa. Jeszcze raz dziękuję, życzę miłego dnia.**

| 1. *PYTANIE DO PROWADZĄCEGO: Czy rozmówca odpowiadał na pytania w sposób racjonalny? Czy podczas rozmowy zaistniały problemy poprzez które zebrane informacje mogą być niejasne lub nieważne ?* | 1 = BRAK PROBLEMÓW  2 = POTENCJALNE PROBLEMY (opisz poniżej): _________________________________________________  _________________________________________________  3 = POWAŻNE PROBLEMY (opisz poniżej): _________________________________________________  _________________________________________________ |  |
| --- | --- | --- |

*INFORMACJA DLA PROWADZĄCEGO: PROSZĘ WRÓCIĆ DO PIERWSZEJ STRONY KWESTIONARIUSZA I UPEWNIĆ SIĘ ŻE WSZYSTKIE ELEMENTY ANKIETY SĄ WYPEŁNIONE POPRAWNIE. JEŚLI TO KONIECZNE, NALEŻY PONOWNIE SKONTAKTOWAĆ SIĘ Z ROZMÓWCĄ ABY ROZWIĄZAĆ PROBLEM.*
